# Synthesis and Antibacterial Activity of Alkylamine-Linked Pleuromutilin Derivatives

**DOI:** 10.3390/antibiotics13111018

**Published:** 2024-10-29

**Authors:** Kerrin Hainsworth, Melissa M. Cadelis, Florent Rouvier, Jean Michel Brunel, Brent R. Copp

**Affiliations:** 1 School of Chemical Sciences, The University of Auckland, Private Bag 92019, Auckland 1142, New Zealandm.cadelis@auckland.ac.nz (M.M.C.); 2 School of Medical Sciences, The University of Auckland, Private Bag 92019, Auckland 1142, New Zealand; 3 Membranes et Cibles Thérapeutiques, SSA, INSERM, Aix-Marseille Universite, 27 bd Jean Moulin, 13385 Marseille, Francejean-michel.brunel@inserm.fr (J.M.B.)

**Keywords:** pleuromutilin, potentiation, adjuvant, antimicrobial, antibiotics, antifungal agents, structure–activity relationships

## Abstract

In an effort to expand the spectrum of the antibacterial activity of pleuromutilin, a series of amine- and polyamine-linked analogues were prepared and evaluated for activities against a panel of microorganisms. Simple C-22-substituted amino esters or diamines **16**, **17**, **18**, and **22**, as well as two unusual amine-linked bis-pleuromutilin examples **20** and **23**, displayed variable levels of activity towards *Staphylococcus aureus* ATCC 25923 and methicillin-resistant *S. aureus*, but with no detectable activities towards Gram-negative bacteria. Fortunately, the incorporation of a longer-chain triamine or polyamine (spermine) at C-22 did afford analogues (**30**, **31**) that exhibited activity towards both *S. aureus* ATCC 25923 and *Escherichia coli* ATCC 25922 with MIC 6.1–13.4 µM. Spermine–pleuromutilin analogue **31** was also able to enhance the action of doxycycline towards *Pseudomonas aeruginosa* ATCC 27853 by eight-fold, highlighting it as a useful scaffold for the development of new antibacterial pleuromutilin analogues that exhibit a broader spectrum of activity.

## 1. Introduction

There has been a rapid rise in antimicrobial resistance (AMR), threatening the efficacy of antibiotics and leading to a global health crisis [1,2]. Resistant pathogens are responsible for killing over 1 million people each year, with that number expected to increase to 10 million by 2050 [3,4]. This crisis can be attributed to the extensive overuse and misuse of antibiotics, as well as a decline in new drug development [2]. Thus, new antibacterial therapies, particularly those with unique mechanisms of action and therefore reduced cross-resistance, are urgently needed.

**Figure 1:**
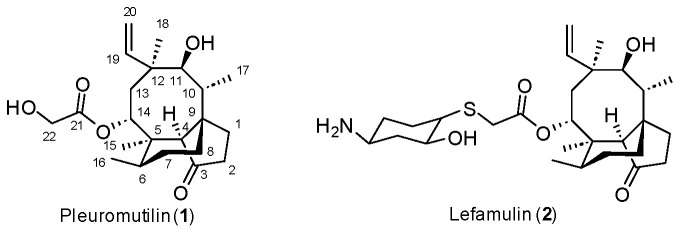
Structures of pleuromutilin **1** and semisynthetic derivative lefamulin (**2**).

Pleuromutilin (**1**) (Figure 1) is a diterpenoid natural product antibiotic that was first isolated from *Pleurotus mutilis* (now *Clitopilus scyphoides*) in the 1950s [5]. Pleuromutilin consists of a 5-6-8 tricyclic mutilin core and a C-14 glycolate side chain [6]. Derivatives of pleuromutilin are readily accessible by modification of the side chain, a process that has been used to discover new more bioactive variants [7], including lefamulin (**2**) (Figure 1), valnemulin, retapamulin, and tiamulin [8,9,10].

Pleuromutilin and its derivatives exhibit antibacterial activity against Gram-positive and mycoplasma bacteria through the inhibition of protein synthesis by binding to the peptidyl transferase centre (PTC) in the 50S bacterial ribosomal subunit [11,12]. The tricyclic core binds within a hydrophobic pocket near the A-Site, while the C-14 side chain extends towards the P-site, sterically hindering peptide bond formation [6]. Due to this unusual mechanism of action and unique binding site, pleuromutilins lack shared resistance mechanisms with other classes of antibiotics, which, when combined with a very low spontaneous mutation frequency, makes them an attractive class of antibiotic for further improvements to combat AMR. [6,13] While the same PTC target is present in Gram-negative bacteria, pleuromutilins suffer from a lack of cell entry or accumulation, making them ineffective against Gram-negative bacteria. A further limiting factor is that they are substrates for a variety of bacterial efflux pumps, and so pleuromutilins can be classified as Gram-positive only antibiotics [6]. Two recent publications suggest that increasing the presence of primary amines and positive charges within small-molecule antibiotics can lead to an increased uptake and accumulation of Gram-negative bacteria, potentially turning lipophilic antibiotics that are Gram-positive only into more broad-spectrum variants [14,15].

There is extensive research concerning the structure–activity relationship (SAR) of pleuromutilins, with the overall conclusion being that C-14 extensions containing thioethers and tertiary amine moieties can often display improved antibacterial activity [7,16]. Semisynthetic analogues that contain C-22 thioether linkages but also incorporate a diaminoalkane substitution at C-19 do show enhanced activity towards the Gram-negative bacterium *Escherichia coli*, suggesting that a judicious placement of an amino functionality on the pleuromutilin scaffold can lead to a broader spectrum of antibacterial activity [7]. At odds with the requirement of C-22 thioether functionality for optimal activity are a number of reports describing C-22 piperazine-linked analogues that exhibit strong in vitro growth inhibition of the Gram-positive bacteria *Staphylococcus aureus* and Methicillin-resistant *S. aureus* (MRSA), as well as in vivo efficacy towards MRSA infection [17,18]. As noted by several groups, the inclusion of a piperazine linker in pleuromutilin also provides the opportunity to append other bioactive functionalities, including cinnamates, [19], amino acids [20], and even oxazolidinone antibiotics [21].

We have recently reported that α,ω-disubstituted polyamines bearing two pleuromutilin end groups exhibit anti-*Staphylococcus* activities, with one example containing a 3-10-3 polyamine core and also exhibiting activity towards *E. coli* [22]. Encouraged by this result and the promising results of piperazine-linked pleuromutilins, we have synthesized a series of semisynthetic derivatives of pleuromutilin that explore several fundamental aspects of variation at C-22, including (1) the direct comparison of biological activities between analogues with thioether and amine moieties, including those with functionality to allow for the preparation of chain-extended variants, (2) the effect of incorporating both primary and secondary amines into the C-22 extension, and (3) the effect of adding lipophilic head groups to the C-22 extension of polyamine–pleuromutilin conjugates. All derivatives were evaluated for antimicrobial activities against a set of Gram-positive and Gram-negative bacteria and for cytotoxicity and human red blood cell hemolytic properties.

## 2. Materials and Methods

### 2.1. Chemistry Synthesis General Methods

Mass spectra were recorded using a MicrOTOF-QII mass spectrometer (Bruker Daltonics, Bremen, Germany) coupled with a KD Scientific syringe pump. Infrared spectra were obtained as dry solids or dry films and recorded on a Perkin Elmer Spectrum 100 Fourier Transform infrared spectrometer equipped with a universal ATR accessory. Melting points were obtained on an electrothermal melting point apparatus and are uncorrected. NMR spectra were recorded using a Bruker Avance 400 MHz spectrometer operating at 400.13 MHz for ^1^H nuclei and 100.62 MHz for ^13^C nuclei. Proto-deutero solvent signals were used as internal references (DMSO-*d*_6_: *δ*_H_ 2.50, *δ*_C_ 39.52; CDCl_3_: *δ*_H_ 7.26, *δ*_C_ 77.16; CD_3_OD: *δ*_H_ 3.31, *δ*_C_ 49.00). Assignments are based on 1- and 2-dimensional NMR experiments and analogue comparisons. Standard Bruker pulse sequences were utilized. Flash column chromatography was carried out with Davisil silica gel (40–63 µm) (Merck), diol-bonded silica gel (40–63 µm) (Merck), or LiChroPrep RP-8 (40–63 µm) (Merck). Analytical thin-layer chromatography (TLC) was carried out on 0.2 mm thick plates of DC-plastikfolien Kieselgel 60 F_254_ (Merck) or DC-Kieselgel 60 RP-18 F_254_S (Merck) plates. Optical rotations were recorded on an Autopol IV polarimeter (Rudolph Research Analytical) using a 1 dm cell. Pleuromutilin was purchased from AK Scientific (Union City, CA, USA) and used as supplied. The protected polyamines *tert*-butyl (3-aminopropyl)(7-((*tert*-butoxycarbonyl)amino)heptyl)carbamate (**25**), *tert*-butyl (3-aminopropyl)(10-((*tert*-butoxycarbonyl)amino)decyl)carbamate (**26**) [23], and *tert*-butyl (4-((3-aminopropyl)(*tert*-butoxycarbonyl)amino)butyl)(3-((*tert*-butoxycarbonyl)amino)propyl)carbamate (**27**) [24] and the pleuromutilin analogue tiamulin (**10**) [22] were synthesized using previously published protocols. 

### 2.2. Synthesis of Compounds

General procedure A—nucleophilic substitution

KI (1.1–2 eq.) was added to a solution of 22-OTs pleuromutilin (**3**) in anhydrous MeCN, and the mixture was stirred under reflux and N_2_ atmosphere for 30 min. Then, the appropriate thiol or amine nucleophile (0.5–1.5 eq.) and DIPEA (6–9 eq.) were added and the reaction was stirred for a further 2–4 h. The solvent was removed under reduced pressure and the residue was extracted with CH_2_Cl_2_, washed with sat. aq. NaHCO_3_ and H_2_O, dried over MgSO_4_, and concentrated under reduced pressure. 

General procedure B—Boc deprotection

A solution of *tert*-butyl-carbamate intermediate in anhydrous CH_2_Cl_2_ (2 mL) and TFA (0.2 mL) was stirred at rt under N_2_ atmosphere for 2 h then concentrated under reduced pressure.

General procedure C—peptide coupling

Variant 1: A solution of EDC.HCl (1.3 eq.), DMAP (1.5 eq.), and carboxylic acid (1.0 eq.) in anhydrous CH_2_Cl_2_ was stirred at 0 °C under N_2_ atmosphere for 30 min, after which amine (1.2 eq.) was added and the mixture was stirred for a further 18 h at rt. The solution was diluted with CH_2_Cl_2_ before being washed with sat. aq. NaHCO_3_ and H_2_O. The organic layer was dried over MgSO_4,_ and the solvent was removed under reduced pressure.

Variant 2: HBTU (1.2 eq.) was added to a stirred solution of amine (1.1 eq.), carboxylic acid (1.0 eq.), HOBt (3.6 eq.), and DIPEA (6–9 eq.) in anhydrous DMF and the reaction mixture was stirred under N_2_ atmosphere at rt for 2 h before the solvent was removed under vacuum.

#### 2.2.1. Pleuromutilin 22-*O*-tosylate (**3**)

A solution of pleuromutilin (0.50 g, 1.3 mmol), *p*-toluenesulfonylchloride (0.30 g, 1.6 mmol), and DMAP (0.48 g, 3.9 mmol) in anhydrous CH_2_Cl_2_ was stirred at 0 °C for 4 h under N_2_ atmosphere. The reaction was quenched with 1 N HCl and extracted twice with EtOAc. The combined organic layers were then washed with sat. aq. NaHCO_3_, dried with MgSO_4_, and concentrated under reduced pressure. The crude product was purified by diol-bonded silica gel flash column chromatography (20–80% EtOAc/petroleum ether) to afford **3** as a white foam (0.39 g, 57%). ^1^H and ^13^C NMR data agreed with those reported in the literature [25].

#### 2.2.2. Methyl 3-((2-(((3a*R*,4*R*,7*S*,8*S*,9*R*,9a*S*,12*R*)-8-hydroxy-4,7,9,12-tetramethyl-3-oxo-7-vinyldecahydro-4,9a-propanocyclopenta[8]annulen-5-yl)oxy)-2-oxoethyl)thio) propanoate (**8**)

Following general procedure A, pleuromutilin 22-*O*-tosylate (**3**) (73 mg, 0.14 mmol) was preincubated with KI (46 mg, 0.28 mmol) followed by reaction with methyl thioglycolate (**4**) (0.013 mL, 0.15 mmol) and DIPEA (0.15 mL, 0.84 mmol) in anhydrous MeCN to give the crude product as a colourless oil. Purification was achieved by silica gel flash column chromatography (10%–50% EtOAc/hexane) to afford **8** as a colourless oil (22 mg, 34%). R*_f_* = 0.62 (1:1, EtOAc:hexane); ${\left[\alpha \right]}_{D}^{24.2}=+$38.1 (*c* = 0.106, CH_2_Cl_2_); IR (ATR) ν_max_ 3552, 2928, 1727, 1456, 1276, 1114 cm^−1^; ^1^H NMR (CDCl_3_, 400 MHz) *δ* 6.47 (1H, dd, *J* = 17.4, 11.0 Hz, H-19), 5.75 (1H, d, *J* = 8.5 Hz, H-14), 5.34 (1H, dd, *J* = 11.0, 1.5 Hz, H_2_-20a), 5.20 (1H, dd, *J* = 17.4, 1.5 Hz, H_2_-20b), 3.73 (3H, s, OMe), 3.38–3.35 (1H, m, H-11), 3.35 (2H, s, H_2_-23), 3.30 (2H, s, H_2_-22), 2.37–2.30 (1H, m, H-10), 2.27–2.14 (2H, m, H_2_-2), 2.11–2.05 (1H, m, H_2_-13a), 2.09 (1H, br s, H-4), 1.79–1.74 (1H, m, H_2_-8a), 1.68–1.60 (2H, m, H_2_-1a, H-6), 1.56–1.52 (1H, m, H_2_-7a), 1.48–1.46 (1H, m, H_2_-1b), 1.45 (3H, s, H_3_-15), 1.39–1.31 (2H, m, H_2_-7b, H_2_-13b), 1.17 (3H, s, H_3_-18), 1.13–1.08 (1H, m, H_2_-8b), 0.88 (3H, d, *J* = 7.0 Hz, H_3_-17), 0.73 (3H, d, *J* = 6.9 Hz, H_3_-16), OH signal not observed; ^13^C NMR (CDCl_3_, 100 MHz) *δ* 217.1 (C-3), 170.3 (C-24), 168.4 (C-21), 139.2 (C-19), 117.3 (C-20), 74.7 (C-11), 69.7 (C-14), 58.3 (C-4), 52.6 (OMe), 45.6 (C-9), 44.9 (C-13), 44.1 (C-12), 41.9 (C-5), 36.8 (C-6), 36.1 (C-10), 34.6 (C-2), 34.3 (C-22), 33.3 (C-23), 30.5 (C-8), 27.0 (C-7), 26.5 (C-18), 24.9 (C-1), 16.9 (C-16), 15.0 (C-15), 11.6 (C-17); (+)-HRESIMS *m/z* 489.2296 [M+Na]^+^ (calcd for C_25_H_38_O_6_SNa, 489.2281).

#### 2.2.3. Methyl 3-((2-(((3a*R*,4*R*,7*S*,8*S*,9*R*,9a*S*,12*R*)-8-hydroxy-4,7,9,12-tetramethyl-3-oxo-7-vinyldecahydro-4,9a-propanocyclopenta[8]annulen-5-yl)oxy)-2-oxoethyl)thio) propanoate (**9**)

Following general procedure A, 22-OTs pleuromutilin (**3**) (54 mg, 0.10 mmol) was preincubated with KI (34 mg, 0.20 mmol) followed by reaction with methyl-3-mercaptopropionate (**5**) (0.012 mL, 0.11 mmol) and DIPEA (0.11 mL, 0.61 mmol) in anhydrous MeCN to give the crude product as a colourless oil. Purification was achieved by silica gel flash column chromatography (10–50% EtOAc/hexane) to afford **9** as a colourless oil (29 mg, 60%). R*_f_* = 0.67 (1:1, EtOAc:hexane); ${\left[\alpha \right]}_{D}^{24.0}=+$39.0 (*c* = 0.146, CH_2_Cl_2_); IR (ATR) ν_max_ 3457, 2928, 1725, 1456, 1278, 1150, 1115 cm^−1^; ^1^H NMR (CDCl_3_, 400 MHz) *δ* 6.46 (1H, dd, *J* = 17.4, 11.0 Hz, H-19), 5.75 (1H, d, *J* = 8.4 Hz, H-14), 5.34 (1H, dd, *J* = 17.4, 1.5 Hz, H_2_-20a), 5.20 (1H, dd, *J* = 17.4, 1.5 Hz, H_2_-20b), 3.68 (3H, s, OMe), 3.16 (2H, s, H_2_-22), 3.37–3.34 (1H, m, H-11), 2.87 (2H, t, *J* = 7.3 Hz, H_2_-23), 2.62 (2H, t, *J* = 7.2 Hz, H_2_-24), 2.38–2.31 (1H, m, H-10), 2.25–2.16 (2H, m, H_2_-2), 2.11–2.05 (1H, m, H_2_-13a), 2.10 (1H, br s, H-4), 1.80–1.74 (1H, m, H_2_-8a), 1.69–1.63 (2H, m, H_2_-1a, H-6), 1.60–1.53 (1H, m, H_2_-7a), 1.48–1.45 (1H, m, H_2_-1b), 1.45 (3H, s, H_3_-15), 1.38–1.31 (2H, m, H_2_-7b, H_2_-13b), 1.17 (3H, s, H_3_-18), 1.15–1.09 (1H, m, H_2_-8b), 0.88 (3H, d, *J* = 7.0 Hz, H_3_-17), 0.73 (3H, d, *J* = 6.9 Hz, H_3_-16), OH signal not observed; ^13^C NMR (CDCl_3_, 100 MHz) *δ* 217.1 (C-3), 172.1 (C-25), 168.8 (C-21), 139.1 (C-19), 117.3 (C-20), 74.8 (C-11), 69.5 (C-14), 58.3 (C-4), 51.9 (OMe), 45.6 (C-9), 44.9 (C-13), 44.0 (C-12), 41.9 (C-5), 36.9 (C-6), 36.1 (C-10), 34.6 (C-2), 34.5 (C-24), 34.2 (C-22), 30.6 (C-8), 27.6 (C-23), 27.0 (C-7), 26.5 (C-18), 25.0 (C-1), 16.9 (C-16), 15.0 (C-15), 11.6 (C-17); (+)-HRESIMS *m/z* 503.2428 [M+Na]^+^ (calcd for C_26_H_40_O_6_SNa, 503.2438).

#### 2.2.4. (3a*R*,4*R*,5*R*,7*S*,8*S*,9*R*,9a*S*,12*R*)-8-Hydroxy-4,7,9,12-tetramethyl-3-oxo-7-vinyldecahydro-4,9a-propanocyclopenta[8]annulen-5-yl 2-((2-aminoethyl)thio)acetate (**11**)

Following general procedure A, pleuromutilin 22-*O*-tosylate (**3**) (130 mg, 0.24 mmol) was preincubated with KI (46 mg, 0.27 mmol) followed by reaction with 2-aminoethane thiol (**7**) hydrochloride (31 mg, 0.27 mmol) and DIPEA (0.25 mL, 1.5 mmol) in anhydrous MeCN to give the crude product as a yellow oil. Purification was achieved by diol-bonded silica gel flash column chromatography (75–100% EtOAc/hexane followed by 100% MeOH) to afford **11** as a colourless oil (52 mg, 50%). R*_f_* = 0.31 (1:9, MeOH:CH_2_Cl_2_); ${\left[\alpha \right]}_{D}^{23.2}=+$93.1 (*c* = 0.153, CH_2_Cl_2_); IR (ATR) ν_max_ 3363, 2932, 2865, 1717, 1604, 1455, 1279, 1116 cm^−1^; ^1^H NMR (CDCl_3_, 400 MHz) *δ* 6.48 (1H, dd, *J* = 17.4, 11.0 Hz, H-19), 5.75 (1H, d, *J* = 8.5 Hz, H-14), 5.35 (1H, dd, *J* = 11.0, 1.5 Hz, H_2_-20a), 5.21 (1H, dd, *J* = 17.4, 1.5 Hz, H_2_-20b), 3.36 (1H, d, *J* = 6.6 Hz, H-11), 3.14 (2H, s, H_2_-22), 2.89 (2H, t, *J* = 6.2 Hz, H_2_-24), 2.70 (2H, t, *J* = 6.2 Hz, H_2_-23), 2.38–2.31 (1H, m, H-10), 2.28–2.16 (2H, m, H_2_-2), 2.10 (1H, br s, H-4), 2.12–2.06 (1H, m, H_2_-13a), 1.79–1.74 (1H, m, H_2_-8a), 1.69–1.61 (2H, m, H_2_-1a, H-6), 1.58–1.53 (1H, m, H_2_-7a), 1.51–1.46 (1H, m, H_2_-1b), 1.45 (3H, s, H_3_-15), 1.42–1.35 (2H, m, H_2_-7b, H_2_-13b), 1.17 (3H, s, H_3_-18), 1.16–1.09 (1H, m, H_2_-8b), 0.88 (3H, d, *J* = 7.1 Hz, H_3_-17), 0.74 (3H, d, *J* = 7.0 Hz, H_3_-16), NH_2_ and OH signals not observed; ^13^C NMR (CDCl_3_, 100 MHz) *δ* 217.1 (C-3), 169.0 (C-21), 139.2 (C-19), 117.1 (C-20), 74.6 (C-11), 69.4 (C-14), 58.3 (C-4), 45.5 (C-9), 44.9 (C-13), 44.0 (C-12), 41.8 (C-5), 40.4 (C-24), 36.8 (C-6), 36.4 (C-23), 36.0 (C-10), 34.5 (C-2), 34.0 (C-22) 30.4 (C-8), 26.8 (C-7), 26.5 (C-18), 24.8 (C-1), 16.8 (C-16), 14.9 (C-15), 11.5 (C-17); (+)-HRESIMS *m*/*z* 460.2493 [M+Na]^+^ (calcd for C_24_H_39_NO_4_SNa, 460.2492).

#### 2.2.5. (3a*R*,4*R*,5*R*,7*S*,8*S*,9*R*,9a*S*,12*R*)-8-Hydroxy-4,7,9,12-tetramethyl-3-oxo-7-vinyl decahydro-4,9a-propanocyclopenta[8]annulen-5-yl (2-methoxy-2-oxoethyl)glycinate (**16**)

Following general procedure A, pleuromutilin 22-*O*-tosylate (**3**) (70 mg, 0.13 mmol) was preincubated with KI (24 mg, 0.14 mmol) followed by reaction with methyl glycinate (**12**) hydrochloride (25 mg, 0.20 mmol) and DIPEA (0.21 mL, 1.2 mmol) in anhydrous MeCN to give the crude product as a pale yellow oil. Purification was achieved by silica gel flash column chromatography 20%–60% EtOAc/hexane) to afford **16** as a colourless oil (12 mg, 20%). R*_f_* = 0.36 (1:1, EtOAc:hexane); ${\left[\alpha \right]}_{D}^{24.6}=+$4.8 (*c* = 0.172, CH_2_Cl_2_); IR (ATR) ν_max_ 3457, 2926, 1729, 1455, 1374, 1195 cm^−1^; ^1^H NMR (CDCl_3_, 400 MHz) *δ* 6.49 (1H, dd, *J* = 17.4, 11.0 Hz, H-19), 5.78 (1H, d, *J* = 8.5 Hz, H-14), 5.33 (1H, dd, *J* = 11.0, 1.4 Hz, H_2_-20a), 5.19 (1H, dd, *J* = 17.4, 1.5 Hz, H_2_-20b), 3.71 (3H, s, OMe), 3.43 (2H, q, *J* = 17.2 Hz, H_2_-24), 3.35 (2H, ABq, Δδ_AB_ = 0.08, *J*_AB_ = 17.5 Hz, H_2_-22), 3.34 (1H, br s, H-11), 2.37–2.30 (1H, m, H-10), 2.24–2.17 (2H, m, H_2_-2), 2.09 (1H, br s, H-4), 2.09–2.03 (1H, m, H_2_-13a), 1.79–1.73 (1H, m, H_2_-8a), 1.68–1.60 (2H, m, H_2_-1a, H-6), 1.59–1.44 (2H, m, H_2_-1b, H_2_-7a), 1.42 (3H, s, H_3_-15), 1.41–1.33 (1H, m, H_2_-7b), 1.31–1.27 (1H, m, H_2_-13b), 1.15 (3H, s, H_3_-18), 1.12–1.08 (1H, m, H_2_-8b), 0.87 (3H, d, *J* = 7.2 Hz, H_3_-17), 0.70 (3H, d, *J* = 6.9 Hz, H_3_-16), NH and OH signals not observed; ^13^C NMR (CDCl_3_, 100 MHz) *δ* 217.1 (C-3), 172.2 (C-25), 170.7 (C-21), 139.1 (C-19), 117.3 (C-20), 74.7 (C-11), 68.8 (C-14), 58.3 (C-4), 52.0 (OMe), 51.0 (C-22), 50.1 (C-24), 45.6 (C-9), 45.0 (C-13), 44.1 (C-12), 41.9 (C-5), 36.8 (C-6), 36.1 (C-10), 34.5 (C-2), 30.5 (C-8), 26.9 (C-7), 26.4 (C-18), 24.9 (C-1), 16.8 (C-16), 14.9 (C-15), 11.6 (C-17); (+)-HRESIMS *m/z* 472.2671 [M+Na]^+^ (calcd for C_25_H_39_NO_6_Na, 472.2670).

#### 2.2.6. Methyl 3-((2-(((3a*R*,4*R*,5*R*,7*S*,8*S*,9*R*,9a*S*,12*R*)-8-hydroxy-4,7,9,12-tetramethyl-3-oxo-7-vinyldecahydro-4,9a-propanocyclopenta[8]annulen-5-yl)oxy)-2-oxoethyl)amino) propanoate (**17**)

Following general procedure A, pleuromutilin 22-*O*-tosylate (**3**) (20 mg, 0.040 mmol) was preincubated with KI (8 mg, 0.044 mmol) followed by reaction with methyl-3-aminopropanoate (**13**) hydrochloride (8 mg, 0.060 mmol) and DIPEA (0.063 mL, 0.36 mmol) in anhydrous MeCN to give the crude products as pale orange oils. Purification was achieved by silica gel flash column chromatography (20%–60% EtOAc/hexane) to afford **17** as a colourless oil (6.5 mg, 16%). R*_f_* = 0.33 (1:1, EtOAc:hexane); ${\left[\alpha \right]}_{D}^{24.4}=+$32.0 (*c* = 0.194, CH_2_Cl_2_); IR (ATR) ν_max_ 3546, 2930, 2865, 1727, 1455 cm^−1^; ^1^H NMR (CDCl_3_, 400 MHz) *δ* 6.50 (1H, dd, *J* = 17.4, 11.0 Hz, H-19), 5.78 (1H, d, *J* = 8.5 Hz, H-14), 5.34 (1H, dd, *J* = 11.0, 1.5 Hz, H_2_-20a), 5.19 (1H, dd, *J* = 17.4, 1.6 Hz, H_2_-20b), 3.67 (3H, s, OMe), 3.35 (1H, d, *J* = 6.5 Hz, H-11), 3.30 (2H, ABq, Δδ_AB_ = 0.08, *J*_AB_ = 17.5 Hz, H_2_-22), 2.94–2.78 (2H, m, H_2_-23), 2.49 (2H, t, *J* = 6.6 Hz, H_2_-25), 2.38–2.31 (1H, m, H-10), 2.26–2.13 (2H, m, H_2_-2), 2.10 (1H, br s, H-4), 2.09–2.03 (1H, m, H_2_-13a), 1.79–1.73 (1H, m, H_2_-8a), 1.68–1.59 (2H, m, H_2_-1a, H-6), 1.56–1.52 (1H, m, H_2_-7a), 1.50–1.45 (1H, m, H_2_-1b), 1.43 (3H, s, H_3_-15), 1.38–1.27 (1H, m, H_2_-7b, H_2_-13b), 1.15 (3H, s, H_3_-18), 1.13–1.08 (1H, m, H_2_-8b), 0.87 (3H, d, *J* = 7.2 Hz, H_3_-17), 0.71 (3H, d, *J* = 6.9 Hz, H_3_-16), NH and OH signals not observed; ^13^C NMR (CDCl_3_, 100 MHz) *δ* 217.2 (C-3), 173.0 (C-26), 171.2 (C-21), 139.2 (C-19), 117.3 (C-20), 74.7 (C-11), 68.8 (C-14), 58.3 (C-4), 51.8 (OMe), 51.7 (C-22), 45.6 (C-9), 45.1 (C-13), 44.9 (C-23), 44.1 (C-12), 41.9 (C-5), 36.8 (C-6), 36.2 (C-10), 34.7 (C-25), 34.6 (C-2), 30.6 (C-8), 27.0 (C-7), 26.4 (C-18), 25.0 (C-1), 16.8 (C-16), 15.0 (C-15), 11.6 (C-17); (+)-HRESIMS *m*/*z* 464.2995 [M+H]^+^ (calcd for C_26_H_42_NO_6_, 464.3007). 

#### 2.2.7. (3a*R*,4*R*,5*R*,7*S*,8*S*,9*R*,9a*S*,12*R*)-8-Hydroxy-4,7,9,12-tetramethyl-3-oxo-7-vinyl decahydro-4,9a-propanocyclopenta[8]annulen-5-yl (2-(diethylamino)ethyl)glycinate (**18**)

Following general procedure A pleuromutilin 22-*O*-tosylate (**3**) (140 mg, 0.26 mmol) was preincubated with KI (48 mg, 0.29 mmol) followed by reaction with *N*,*N*-diethylethylenediamine (**14**) (0.055 mL, 0.39 mmol) and DIPEA (0.37 mL, 2.1 mmol) in anhydrous MeCN to give the crude product as an orange oil. Purification was achieved by diol-bonded silica gel column chromatography (75–100% EtOAc/hexane followed by 100% MeOH) followed by silica gel flash column chromatography (5–10% MeOH/CH_2_Cl_2_) to afford **18** as a colourless oil (68 mg, 54%). R*_f_* = 0.49 (1:9, MeOH:CH_2_Cl_2_); ${\left[\alpha \right]}_{D}^{23.8}=+$60.3 (*c* = 0.152, CH_2_Cl_2_); IR (ATR) ν_max_ 3543, 2932, 1728, 1456, 1384, 1279 cm^−1^; ^1^H NMR (CDCl_3_, 400 MHz) *δ* 6.52 (1H, dd, *J* = 17.4, 11.0 Hz, H-19), 5.79 (1H, d, *J* = 8.5 Hz, H-14), 5.33 (1H, dd, *J* = 11.0, 1.5 Hz, H_2_-20a), 5.19 (1H, dd, *J* = 17.4, 1.5 Hz, H_2_-20b), 3.35 (1H, d, *J* = 6.4 Hz, H-11), 3.31 (2H, ABq, Δδ_AB_ = 0.07, *J*_AB_ = 17.5 Hz, H_2_-22), 2.71–2.57 (2H, m, H_2_-24), 2.57–2.51 (4H, m, H_4_-26), 2.54 (4H, q, *J* = 7.2 Hz, H_2_-25) 2.39–2.32 (1H, m, H-10), 2.25–2.16 (2H, m, H_2_-2), 2.10 (1H, br s, H-4), 2.10–2.04 (1H, m, H_2_-13a), 1.79–1.75 (1H, m, H_2_-8a), 1.69–1.60 (2H, m, H_2_-1a, H-6), 1.57–1.54 (1H, m, H_2_-7a), 1.48–1.43 (1H, m, H_2_-1b), 1.45 (3H, s, H_3_-15), 1.38–1.27 (2H, m, H_2_-7b, H_2_-13b), 1.16 (3H, s, H_3_-18), 1.13–1.09 (1H, m, H_2_-8b), 1.01 (3H, t, *J* = 7.1 Hz, H_6_-27), 0.87 (3H, d, *J* = 7.0 Hz, H_3_-17), 0.72 (3H, d, *J* = 6.9 Hz, H_3_-16), NH and OH signal not observed; ^13^C NMR (CDCl_3_, 100 MHz) *δ* 217.2 (C-3), 171.4 (C-21), 139.2 (C-19), 117.3 (C-20), 74.7 (C-11), 68.5 (C-14), 58.3 (C-4), 52.6 (C-26), 51.9 (C-22), 47.1 (C-24, C-25), 45.6 (C-9), 45.1 (C-13), 44.1 (C-12), 41.9 (C-5), 36.9 (C-6), 36.1 (C-10), 34.6 (C-2), 30.6 (C-8), 27.0 (C-7), 26.4 (C-18), 25.0 (C-1), 16.8 (C-16), 15.0 (C-15), 11.7 (C-17), 11.6 (C-27); (+)-HRESIMS *m*/*z* 477.3672 [M+H]^+^ (calcd for C_28_H_49_N_2_O_4_, 477.3687). 

#### 2.2.8. (3a*R*,4*R*,5*R*,7*S*,8*S*,9*R*,9a*S*,12*R*)-8-Hydroxy-4,7,9,12-tetramethyl-3-oxo-7-vinyl decahydro-4,9a-propanocyclopenta[8]annulen-5-yl (2-((tert-butoxycarbonyl)amino) ethyl)glycinate (**19**)

Following general procedure A, pleuromutilin 22-*O*-tosylate (**3**) (70 mg, 0.13 mmol) was preincubated with KI (24 mg, 0.14 mmol) followed by reaction with *tert*-butyl-*N*-(2-aminoethyl)carbamate (**15**) (0.031 mL, 0.20 mmol) and DIPEA (0.18 mL, 1.1 mmol) to give the crude products as an orange oil. Purification was achieved by silica gel flash column chromatography (10%–50% EtOAc/hexane) to afford **19** as a colourless oil (19 mg, 28%). R*_f_* = 0.20 (1:1, EtOAc:hexane); ${\left[\alpha \right]}_{D}^{20.3}$ = +132.6 (*c* = 0.121, CH_2_Cl_2_); IR (ATR) ν_max_ 3364, 2933, 1728, 1696, 1513, 1455, 1196, 1154, 1116, 1017, 912, 729, 646 cm^−1^; ^1^H NMR (CDCl_3_, 400 MHz) *δ* 6.51 (1H, dd, *J* = 17.4, 11.0 Hz, H-19), 5.78 (1H, d, *J* = 8.5 Hz, H-14), 5.35 (1H, dd, *J* = 11.0, 1.4 Hz, H_2_-20a), 5.20 (1H, dd, *J* = 17.4, 1.5 Hz, H_2_-20b), 3.36 (1H, d, *J* = 7.6 Hz, H-11), 3.30 (2H, ABq, Δδ_AB_ = 0.08, *J*_AB_ = 17.6 Hz, H_2_-22), 3.19 (2H, q, *J* = 5.5 Hz, H_2_-25), 2.79–2.64 (2H, m, H_2_-24), 2.39–2.32 (1H, m, H-10), 2.26–2.19 (2H, m, H_2_-2), 2.11–2.05 (1H, m, H_2_-13a), 2.09 (1H, br s, H-4), 1.80–1.75 (1H, m, H_2_-8a), 1.70–1.64 (2H, m, H_2_-1a, H-6), 1.56–1.47 (1H, m, H_2_-7a), 1.44 (4H, s, H_2_-1b, H_3_-15), 1.39–1.36 (9H, m, H_3_-29), 1.31–1.25 (2H, m, H_2_-7b, H_2_-13b), 1.17 (3H, s, H_3_-18), 1.14–1.10 (1H, m, H_2_-8b), 0.88 (3H, d, *J* = 7.0 Hz, H_3_-17), 0.71 (3H, d, *J* = 7.0 Hz, H_3_-16), NH and OH signals not observed; ^13^C NMR (CDCl_3_, 100 MHz) *δ* 217.2 (C-3), 171.4 (C-21), 156.2 (C-27), 139.2 (C-19), 117.4 (C-20a), 77.4 (C-28), 74.8 (C-11), 68.9 (C-14), 58.3 (C-4), 51.3 (C-22), 49.0 (C-24), 45.6 (C-9), 45.1 (C-13a), 44.1 (C-12), 41.9 (C-5), 40.2 (C-25), 36.8 (C-6), 36.2 (C-10), 34.6 (C-2), 30.6 (C-8a), 28.6 (C-29), 27.0 (C-7a), 26.4 (C-18), 25.0 (C-1), 16.9 (C-16), 15.0 (C-15), 11.7 (C-17); (+)-HRESIMS *m*/*z* 543.3408 [M+Na]^+^ (calcd for C_29_H_48_N_2_NaO_6_, 543.3405).

#### 2.2.9. Bis((3a*R*,4*R*,5*R*,7*S*,8*S*,9*R*,9a*S*,12*R*)-8-hydroxy-4,7,9,12-tetramethyl-3-oxo-7-vinyl decahydro-4,9a-propanocyclopenta[8]annulen-5-yl) 2,2′-((3-methoxy-3-oxopropyl) azanediyl)diacetate (**20**)

Following general procedure A, pleuromutilin 22-*O*-tosylate (**3**) (70 mg, 0.13 mmol) was preincubated with KI (24 mg, 0.14 mmol) followed by reaction with methyl-3-aminopropanoate (**13**) hydrochloride (8.0 mg, 0.059 mmol) and DIPEA (0.16 mL, 0.92 mmol) in anhydrous MeCN to give the crude product as a dark orange oil. Purification was achieved by silica gel flash column chromatography eluting with 10%–50% EtOAc/hexane, affording **20** as a colourless oil (10 mg, 19%). R*_f_* = 0.49 (100% EtOAc); ${\left[\alpha \right]}_{D}^{23.6}=+$24.6 (*c* = 0.122, CH_2_Cl_2_); IR (ATR) ν_max_ 2926, 1734, 1195 cm^−1^; ^1^H NMR (CDCl_3_, 400 MHz) *δ* 6.48 (2H, dd, *J* = 17.4, 11.0 Hz, H-19), 5.76 (2H, d, *J* = 8.4 Hz, H-14), 5.34 (2H, dd, *J* = 11.0, 1.4 Hz, H_2_-20a), 5.19 (2H, dd, *J* = 17.4, 1.5 Hz, H_2_-20b), 3.65 (3H, s, OMe), 3.35 (2H, dd, *J* = 10.0, 6.6 Hz, H-11), 3.45 (4H, s, H_2_-22), 3.02 (2H, t, *J* = 7.2 Hz, H_2_-24), 2.47 (2H, t, *J* = 7.2 Hz, H_2_-25), 2.38–2.31 (2H, m, H-10), 2.27–2.19 (4H, m, H_2_-2), 2.08 (2H, br s, H-4), 2.08–2.03 (2H, m, H_2_-13a), 1.79–1.75 (2H, m, H_2_-8a), 1.69–1.61 (4H, m, H_2_-1a, H-6), 1.54–1.51 (2H, m, H_2_-7a), 1.48 (2H, br s, H_2_-1b), 1.42 (6H, s, H_3_-15), 1.38–1.30 (4H, m, H_2_-7b, H_2_-13b), 1.16 (6H, s, H_3_-18), 1.13–1.08 (2H, m, H_2_-8b), 0.88 (6H, d, *J* = 7.0 Hz, H_3_-17), 0.69 (6H, d, *J* = 6.9 Hz, H_3_-16), OH signal not observed; ^13^C NMR (CDCl_3_, 100 MHz) *δ* 217.2 (C-3), 172.6 (C-26), 170.2 (C-21), 139.3 (C-19), 117.3 (C-20), 74.8 (C-11), 68.5 (C-14), 58.4 (C-4), 51.8 (OMe), 55.9 (C-22), 50.5 (C-24), 45.6 (C-9), 45.3 (C-13), 44.1 (C-12), 41.9 (C-5), 36.9 (C-6), 36.2 (C-10), 34.6 (C-2), 33.8 (C-25), 30.6 (C-8), 27.0 (C-7), 26.6 (C-18), 25.0 (C-1), 16.8 (C-16), 15.1 (C-15), 11.6 (C-17); (+)-HRESIMS *m*/*z* 824.5205 [M+H]^+^ (calcd for C_48_H_73_NO_10_, 824.5234).

#### 2.2.10. (3a*R*,4*R*,5*R*,7*S*,8*S*,9*R*,9a*S*,12*R*)-8-hydroxy-4,7,9,12-tetramethyl-3-oxo-7-vinyldecahydro-4,9a-propanocyclopenta[8]annulen-5-yl (2-(l4-azaneyl)ethyl)glycinate, 2,2,2-trifluoroacetate (**22**)

Following general procedure B, Boc-protected analogue **19** was stirred in a solution of CH_2_Cl_2_ (2 mL) and TFA (0.2 mL) to give the crude product as a colourless oil. Purification was achieved by C_8_ reversed-phase flash column chromatography eluting with 0–100% MeOH/H_2_O (0.05% TFA) to give the mono-TFA salt of **22** as a colourless oil (9 mg, 84%). R*_f_* = 0.54 (RP-18, 7:3, MeOH:10% *aq*. HCl); ${\left[\alpha \right]}_{D}^{23.9}=+$5.1 (*c* = 0.178, MeOH); IR (ATR) ν_max_ 3404, 2927, 1731, 1677, 1426, 1202, 1134, 1025 cm^−1^; ^1^H NMR (CD_3_OD, 400 MHz) *δ* 6.34–6.27 (1H, m, H-19), 5.85 (1H, d, *J* = 8.3 Hz, H-14), 5.19 (1H, dd, *J* = 6.3, 1.5 Hz, H_2_-20a), 5.15 (1H, s, H_2_-20b), 4.02 (2H, ABq, Δδ_AB_ = 0.11, *J*_AB_ = 17.0 Hz, H_2_-22), 3.51 (1H, d, *J* = 6.1 Hz, H-11), 3.42–3.33 (4H, m, H_2_-24, H_2_-25), 2.40 (1H, br s, H-4), 2.32–2.11 (4H, m, H_2_-2, H-10, H_2_-13a), 1.84–1.80 (1H, m, H_2_-8a), 1.72–1.64 (2H, m, H_2_-1a, H-6), 1.60–1.49 (1H, m, H_2_-7a), 1.49–1.44 (1H, m, H_2_-1b), 1.46 (3H, s, H_3_-15), 1.44–1.36 (2H, m, H_2_-7b, H_2_-13b), 1.20–1.12 (1H, m, H_2_-8b), 1.16 (3H, s, H_3_-18), 0.94 (3H, d, *J* = 7.0 Hz, H_3_-17), 0.75 (3H, d, *J* = 7.0 Hz, H_3_-16); Exchangeable ^1^H signals observed: ^1^H NMR (DMSO-*d*_6_, 400 MHz) *δ* 9.32 (1H, br s, NH-23), 7.91 (3H, br s, NH_3_-26), 4.60 (1H, br s, OH-27); ^13^C NMR (CD_3_OD, 100 MHz) *δ* 219.3 (C-3), 167.6 (C-21), 141.1 (C-19), 116.8 (C-20), 75.3 (C-11), 73.0 (C-14), 59.1 (C-4), 49.6 (C-22), 45.6 (C-24), 46.8 (C-9), 45.7 (C-13), 45.4 (C-5), 43.2 (C-12), 38.0 (C-6), 37.8 (C-10), 37.2 (C-25), 35.3 (C-2), 31.5 (C-8), 28.2 (C-18), 28.0 (C-7), 25.8 (C-1), 17.1 (C-16), 15.2 (C-15), 11.8 (C-17); (+)-HRESIMS *m*/*z* 421.3051 [M+H]^+^ (calcd for C_24_H_41_N_2_O_4_, 421.3061).

#### 2.2.11. Bis(2-(((3a*R*,4*R*,5*R*,7*S*,8*S*,9*R*,9a*S*,12*R*)-8-hydroxy-4,7,9,12-tetramethyl-3-oxo-7-vinyl decahydro-4,9a-propanocyclopenta[8]annulen-5-yl)oxy)-2-oxoethyl)amino)ethan-1-aminium 2,2,2-trifluoroacetate salt (**23**)

Following general procedure A, pleuromutilin 22-*O*-tosylate (**3**) (130 mg, 0.24 mmol) was preincubated with KI (45 mg, 0.26 mmol) followed by reaction with *tert*-butyl-*N*-(2-aminoethyl)carbamate (**15**) (0.019 mL, 0.12 mmol) and DIPEA (0.38 mL, 2.2 mmol) in anhydrous MeCN to give the crude product as a dark red oil. Purification was achieved by silica gel flash column chromatography (10%–100% EtOAc/hexane) to give **21** as a pale-yellow oil (22 mg, 20%). Following general procedure B, **21** (10 mg, 0.02 mmol) was stirred in a solution of CH_2_Cl_2_ (2 mL) and TFA (0.2 mL) to give the TFA salt of **23** as an orange foam (20 mg, 91%). R*_f_* = 0.63 (RP-18, 7:3, MeOH:10% *aq*. HCl); ${\left[\alpha \right]}_{D}^{24.3}=+$6.5 (*c* = 0.101, MeOH); IR (ATR) ν_max_ 3348, 2932, 1729, 1673, 1546, 1453, 1423, 1203 cm^−1^; ^1^H NMR (CD_3_OD, 400 MHz) *δ* 6.35 (2H, dd, *J* = 17.4, 11.3 Hz, H-19), 5.79 (2H, d, *J* = 8.3 Hz, H-14), 5.21–5.20 (2H, m, H_2_-20a), 5.17 (2H, dd, *J* = 9.5, 1.5 Hz, H_2_-20b), 3.50 (2H, d, *J* = 6.0 Hz, H-11), 3.46 (2H, ABq, Δδ_AB_ = 0.06, *J*_AB_ = 18.2 Hz, H_2_-22), 3.02–2.92 (4H, m, H_2_-24, H_2_-25), 2.37 (2H, br s, H-4), 2.35–2.24 (2H, m, H-10), 2.19–2.10 (6H, m, H_2_-2, H_2_-13a), 1.84–1.80 (2H, m, H_2_-8a), 1.74–1.62 (4H, m, H_2_-1a, H-6), 1.60–1.52 (2H, m, H_2_-7a), 1.49–1.46 (2H, m, H_2_-1b), 1.42 (6H, s, H_3_-15), 1.40–1.28 (4H, m, H_2_-7b, H_2_-13b), 1.20–1.13 (2H, m, H_2_-8b), 1.16 (6H, s, H_3_-18), 0.93 (6H, d, *J* = 7.0 Hz, H_3_-17), 0.71 (6H, d, *J* = 6.6 Hz, H_3_-16); Exchangeable ^1^H signal observed: ^1^H NMR (DMSO-*d*_6_, 400 MHz) *δ* 7.60 (3H, br s, NH_3_-26); ^13^C NMR (CD_3_OD, 100 MHz) *δ* 219.5 (C-3), 172.5 (C-21), 141.4 (C-19), 116.6 (C-20), 75.4 (C-11), 71.2 (C-14), 59.2 (C-4), 57.8 (C-22), 53.5 (C-24), 46.8 (C-9), 46.1 (C-13), 45.4 (C-5), 43.1 (C-12), 38.9 (C-25), 38.0 (C-6), 37.8 (C-10), 35.3 (C-2), 31.5 (C-8), 28.3 (C-18), 28.1 (C-7), 25.8 (C-1), 17.1 (C-16), 15.3 (C-15), 11.8 (C-17); (+)-HRESIMS *m*/*z* 781.5362 [M+H]^+^ (calcd for C_46_H_73_N_2_O_8_, 781.5361).

#### 2.2.12. *N*^1^-(2-(((3a*R*,4*R*,5*R*,7*S*,8*S*,9*R*,9a*S*,12*R*)-8-Hydroxy-4,7,9,12-tetramethyl-3-oxo-7-vinyldecahydro-4,9a-propanocyclopenta[8]annulen-5-yl)oxy)-2-oxoethyl)hexane-1,6-diaminium 2,2,2-trifluoroacetate (**28**)

Following general procedure A, pleuromutilin 22-*O*-tosylate (**3**) (200 mg, 0.38 mmol) was preincubated with KI (68 mg, 0.41 mmol) followed by reaction with *tert*-butyl (6-aminohexyl)carbamate (**24**) (0.12 mL, 0.56 mmol) and DIPEA (0.52 mL, 3.0 mmol). Purification was achieved by silica gel flash column chromatography (20–70% EtOAc/hexane) to afford a Boc-protected intermediate as a pale-yellow foam (106 mg, 59%). Following general procedure B, a sub-sample of this intermediate (100 mg, 0.17 mmol) was reacted with TFA (0.2 mL) and CH_2_Cl_2_ (2 mL) to afford the di-TFA salt of **28** as an orange foam (120 mg, 95%). R*_f_* = 0.82 (RP-18, 9:1, MeOH:10% *aq*. HCl); ${\left[\alpha \right]}_{D}^{24.1}$ = +5.1 (*c* = 0.175, MeOH); IR (ATR) ν_max_ 2936, 1733, 1669, 1424, 1236, 1198, 1176, 1128 cm^−1^; ^1^H NMR (CD_3_OD, 400 MHz) *δ* 6.33 (1H, dd, *J* = 18.0, 10.6 Hz, H-19), 5.85 (1H, d, *J* = 8.4 Hz, H-14), 5.19 (2H, dd, *J* = 5.4, 1.5 Hz, H_2_-20a), 5.15 (1H, s, H_2_-20b), 3.91 (2H, ABq, Δ*δ*_AB_ = 0.12, *J*_AB_ = 17.2 Hz, H_2_-22), 3.51 (1H, br s, H_1_-11), 3.05–3.01 (2H, m, H_2_-24), 2.92 (2H, t, *J* = 7.6 Hz, H_2_-29), 2.40 (1H, br s, H-4), 2.32–2.11 (4H, m, H_2_-2, H-10, H_2_-13a), 1.84–1.80 (1H, m, H_2_-8a), 1.74–1.60 (7H, m, H_2_-1a, H-6, H_2_-7a, H_2_-27, H_2_-28), 1.54–1.49 (4H, m, H_2_-25, H_2_-26), 1.44–1.40 (3H, m, H_2_-1b, H_2_-7b, H_2_-13b), 1.46 (3H, s, H_3_-15), 1.20–1.12 (1H, m, H_2_-8b), 1.16 (3H, s, H_3_-18), 0.94 (3H, d, *J* = 7.0 Hz, H_3_-17), 0.74 (3H, d, *J* = 6.9 Hz, H_3_-16); Exchangeable ^1^H signals observed: ^1^H NMR (DMSO-*d*_6_, 400 MHz) *δ* 8.99 (2H, br s, NH_2_-23), 7.64 (3H, br s, NH_3_-30); ^13^C NMR (CD_3_OD, 100 MHz) *δ* 219.3 (C-3), 166.8 (C-21), 141.1 (C-19), 116.7 (C-20), 75.3 (C-11), 73.0 (C-14), 59.0 (C-4), 48.9 (C-22^a^), 48.5 (C-23^a^), 46.7 (C-9), 45.7 (C-13), 45.3 (C-5), 43.1 (C-12), 40.5 (C-28), 37.9 (C-10), 37.7 (C-6), 35.2 (C-2), 31.4 (C-8), 28.2 (C-25^b^), 28.1 (C-26^b^), 28.0 (C-18), 27.0 (C-24^c^), 26.9 (C-27^c^), 26.7 (C-7), 25.8 (C-1), 17.0 (C-16), 15.2 (C-15), 11.8 (C-17); (+)-HRESIMS *m*/*z* 477.3684 [M+H]^+^ (calcd for C_28_H_49_N_2_O_4_, 477.3687).^a,b,c^ Interchangeable assignments that could not be distinguished. 

#### 2.2.13. *N*^1^-(3-((2-(((3a*R*,4*R*,5*R*,7*S*,8*S*,9*R*,9a*S*,12*R*)-8-hydroxy-4,7,9,12-tetramethyl-3-oxo-7-vinyldecahydro-4,9a-propanocyclopenta[8]annulen-5-yl)oxy)-2-oxoethyl) ammonio)propyl)heptane-1,7-diaminium 2,2,2-trifluoroacetate (**29**)

Following general procedure A, pleuromutilin 22-*O*-tosylate (**3**) (100 mg, 0.19 mmol) was preincubated with KI (34 mg, 0.21 mmol) and then reacted with *tert*-butyl (3-aminopropyl)(7-((*tert*-butoxycarbonyl)amino)heptyl)carbamate (**25**) (110 mg, 0.29 mmol) and DIPEA (0.26 mL, 1.5 mmol). Purification was achieved by silica gel flash column chromatography (20–50% EtOAc/hexane) to afford a Boc-protected intermediate as an orange oil (67 mg, 47%). Following general procedure B, all of this intermediate (67 mg, 0.12 mmol) was reacted with TFA (0.2 mL) and CH_2_Cl_2_ (2 mL). Purification was achieved by C_8_ reversed-phase flash column chromatography 0%–100% MeOH/H_2_O (0.05% TFA) to afford the tri-TFA salt of **29** as an orange foam (90 mg, 92%). R*_f_* = 0.74 (RP-18, 7:3, MeOH:10% *aq*. HCl); ${\left[\alpha \right]}_{D}^{24.3}$ = +52.3 (*c* = 0.122, MeOH); IR (ATR) ν_max_ 3442, 2994, 2942, 2865, 1733, 1672, 1463, 1428, 1234, 1200, 1132, 837, 799, 722 cm^−1^; ^1^H NMR (CD_3_OD, 400 MHz) *δ* 6.31 (1H, dd, *J* = 18.1, 10.7 Hz, H-19), 5.84 (1H, d, *J* = 8.4 Hz, H-14), 5.19 (1H, dd, *J* = 6.1, 1.5 Hz, H_2_-20a), 5.15 (1H, s, H_2_-20b), 3.94 (2H, ABq, Δδ_AB_ = 0.14, *J*_AB_ = 17.1 Hz, H_2_-22), 3.51 (1H, d, *J* = 6.0 Hz, H-11), 3.19–3.09 (4H, m, H_2_-24, H_2_-26), 3.01 (2H, t, *J* = 7.9 Hz, H_2_-28), 2.91 (2H, t, *J* = 7.6 Hz, H_2_-34), 2.39 (1H, s, H-4), 2.32–2.27 (2H, m, H_2_-2), 2.24–2.20 (1H, m, H_2_-13a), 2.20–2.11 (3H, m, H-10, H_2_-25), 1.84–1.80 (1H, m, H_2_-8a), 1.74–1.64 (7H, m, H-1a, H-6, H_2_-7b, H_2_-29, H_2_-33), 1.46 (3H, s, H_3_-15), 1.43–1.37 (9H, m, H-1b, H-7a, H-13b, H_2_-30, H_2_-31, H_2_-32), 1.20–1.12 (1H, m, H-8b), 1.16 (3H, s, H_3_-18), 0.94 (3H, d, *J* = 7.0 Hz, H_3_-17), 0.75 (3H, d, *J* = 7.0 Hz, H_3_-16); Exchangeable ^1^H signals observed: ^1^H NMR (DMSO-*d*_6_, 400 MHz) *δ* 9.33 (2H, br s, NH_2_-23), 8.67 (2H, br s, NH_2_-27), 7.74 (3H, br s, NH_3_-35); ^13^C NMR (CD_3_OD, 100 MHz) *δ* 219.3 (C-3), 166.6 (C-21), 141.0 (C-19), 116.8 (C-20), 75.3 (C-11), 73.1 (C-14), 59.0 (C-4), 49.2 (C-22, C-28), 46.7 (C-9), 45.7 (C-24^a^), 45.6 (C-26^a^), 45.4 (C-13), 43.1 (C-5), 40.7 (C-12), 40.6 (C-34), 37.9 (C-10), 37.7 (C-6), 35.2 (C-2), 31.4 (C-8), 29.5 (C-30^b^), 28.3 (C-32^b^), 28.1 (C-18), 28.0 (C-7), 27.2 (C-29^c^), 27.1 (C-33^c^), 27.0 (C-32^b^), 25.8 (C-1), 23.9 (C-25), 17.0 (C-16), 15.1 (C-15), 11.8 (C-17); (+)-HRESIMS *m*/*z* 548.4423 [M+H]^+^ (calcd for C_32_H_58_N_3_O_4_, 548.4422). ^a,b,c^ Interchangeable assignments that could not be distinguished. 

#### 2.2.14. (3a*R*,4*R*,5*R*,7*S*,8*S*,9*R*,9a*S*,12*R*)-8-hydroxy-4,7,9,12-tetramethyl-3-oxo-7-vinyldecahydro-4,9a-propanocyclopenta[8]annulen-5-yl (3-((7-aminoheptyl)amino)propyl)glycinate (**30**)

Following general procedure A, pleuromutilin 22-*O*-tosylate (**3**) (380 mg, 0.71 mmol) was preincubated with KI (130 mg, 0.78 mmol) and then reacted with *tert*-butyl (3-aminopropyl)(10-((*tert*-butoxycarbonyl)amino)decyl)carbamate (**26**) (460 mg, 1.07 mmol) and DIPEA (1.1 mL, 6.4 mmol). Purification was achieved by silica gel flash column chromatography (50–80% EtOAc/hexane) to afford a Boc-protected intermediate as a yellow oil (300 mg, 54%). Following general procedure B, a sub-sample of this intermediate (130 mg, 0.16 mmol) was reacted with TFA (0.2 mL) and CH_2_Cl_2_ (2 mL) to afford the tri-TFA salt of **30** as an orange crystalline solid (155 mg, 90%). R*_f_* = 0.77 (RP-18, 9:1, MeOH:10% *aq*. HCl); ${\left[\alpha \right]}_{D}^{18.2}$ = +173.3 (*c* = 0.100, MeOH); m.p 157–160 °C; IR (ATR) ν_max_ 3389, 2931, 2861, 1751, 1724, 1673, 1470, 1196, 1171, 1127 cm^−1^; ^1^H NMR (CD_3_OD, 400 MHz) *δ* 6.32 (1H, dd, *J* = 17.1, 11.6 Hz, H-19), 5.85 (1H, d, *J* = 8.4 Hz, H-14), 5.19 (1H, d, *J* = 5.1 Hz, H_2_-20a), 5.15 (1H, s, H_2_-20b), 3.95 (2H, ABq, Δδ_AB_ = 0.14, *J*_AB_ = 17.0 Hz, H_2_-22), 3.51 (1H, d, *J* = 6.0 Hz, H-11), 3.16–3.15 (2H, m, H_2_-26), 3.11 (2H, t, *J* = 7.7 Hz, H_2_-24), 3.00 (2H, t, *J* = 7.7 Hz, H_2_-28), 2.91 (2H, t, *J* = 7.6 Hz, H_2_-37), 2.40 (1H, br s, H-4), 2.32–2.30 (1H, m, H-10), 2.27–2.20 (2H, m, H_2_-2), 2.20–2.11 (3H, m, H_2_-13a, H_2_-25), 1.84–1.80 (1H, m, H_2_-8a), 1.71–1.63 (7H, m, H_2_-1a, H-6, H_2_-7b, H_2_-30, H_2_-36), 1.56–1.53 (2H, m, H_2_-1b, H_2_-13b), 1.46 (3H, s, H_3_-15), 1.41–1.36 (13H, m, H_2_-7a, H_2_-29, H_2_-31, H_2_-32, H_2_-33, H_2_-34, H_2_-35), 1.20–1.12 (1H, m, H_2_-8b), 1.16 (3H, s, H_3_-18), 0.94 (3H, d, *J* = 7.0 Hz, H_3_-17), 0.75 (3H, d, *J* = 7.0 Hz, H_3_-16); Exchangeable ^1^H signals observed: ^1^H NMR (DMSO-*d*_6_, 400 MHz) *δ* 9.38 (2H, br s, NH_2_-23), 8.72 (2H, br s, NH_2_-27), 7.78 (3H, br s, NH_3_-38); ^13^C NMR (CD_3_OD, 100 MHz) *δ* 219.3 (C-3), 166.6 (C-21), 141.0 (C-19), 116.8 (C-20), 75.3 (C-11), 73.1 (C-14), 59.1 (C-4), 49.0 (C-22, C-28), 46.7 (C-9), 45.7 (C-13), 45.6 (C-24, C-26), 45.4 (C-12), 43.2 (C-5), 40.7 (C-37), 37.9 (C-10), 37.8 (C-6), 35.2 (C-2), 31.4 (C-8), 30.30 (C-29^a^), 30.28 (C-31^a^), 30.12 (C-32^a^), 30.10 (C-33^a^), 28.5 (C-30^a^), 28.1 (C-18), 28.0 (C-36^a^), 27.5 (C-34^a^), 27.4 (C-35^a^), 27.2 (C-7), 25.8 (C-1), 23.9 (C-25), 17.0 (C-16), 15.1 (C-15), 11.8 (C-17); (+)-HRESIMS *m*/*z* 590.4889 [M+H]^+^ (calcd for C_35_H_64_N_3_O_4_, 590.4891). ^a^ Interchangeable assignments that could not be distinguished. 

#### 2.2.15. (3a*R*,4*R*,5*R*,7*S*,8*S*,9*R*,9a*S*,12*R*)-8-hydroxy-4,7,9,12-tetramethyl-3-oxo-7-vinyldecahydro-4,9a-propanocyclopenta[8]annulen-5-yl (3-((4-((3-aminopropyl)amino)butyl)amino)propyl)glycinate (**31**)

Following general procedure A, pleuromutilin 22-*O*-tosylate (**3**) (219 mg, 0.41 mmol) was preincubated with KI (75 mg, 0.45 mmol) and then reacted with *tert*-butyl (4-((3-aminopropyl)(tert-butoxycarbonyl)amino)butyl)(3-((tert-butoxycarbonyl)amino)propyl)carbamate (**27**) (310 mg, 0.62 mmol) and DIPEA (0.72 mL, 4.1 mmol). Purification was achieved by silica gel flash column chromatography (20–10% EtOAc/petroleum ether) to afford a Boc-protected intermediate as a white foam (236 mg, 67%). Following general procedure B, a sub-sample of this intermediate (127 mg, 0.15 mmol) was reacted with TFA (0.2 mL) and CH_2_Cl_2_ (2 mL) to afford the tetra-TFA salt of **31** as an orange gum (147 mg, 98%). R*_f_* = 0.84 (RP-18, 9:1, MeOH:10% *aq*. HCl); ${\left[\alpha \right]}_{D}^{20.2}$ = +0.89 (*c* = 0.100, MeOH); IR (ATR) ν_max_ 3448, 2962, 2864, 1779, 1736, 1670, 1477, 1196, 1165, 1127 cm^−1^; ^1^H NMR (CD_3_OD, 400 MHz) *δ* 6.32 (1H, dd, *J* = 18.1, 10.7 Hz, H-19), 5.85 (1H, d, *J* = 8.4 Hz, H-14), 5.19 (1H, dd, *J* = 6.3, 1.5 Hz, H_2_-20a), 5.15 (1H, s, H_2_-20b), 3.94 (2H, ABq, Δδ_AB_ = 0.14, *J*_AB_ = 17.1 Hz, H_2_-22), 3.51 (1H, d, *J* = 6.1 Hz, H-11), 3.17–3.11 (6H, m, H_2_-31, H_2_-33, H_2_-35), 3.09–3.04 (6H, m, H_2_-24, H_2_-26, H_2_-28), 2.40 (1H, br s, H-4), 2.32–2.27 (1H, m, H-10), 2.25–2.18 (2H, m, H_2_-2), 2.16–2.07 (5H, m, H_2_-13a, H_2_-25, H_2_-34), 1.84–1.79 (5H, m, H_2_-8a, H_2_-29, H_2_-30), 1.74–1.64 (2H, m, H_2_-1a, H-6), 1.57–1.53 (1H, m, H_2_-7b), 1.50–1.43 (1H, m, H_2_-1b), 1.46 (3H, s, H_3_-15), 1.41–1.36 (2H, m, H_2_-7a, H_2_-13b), 1.20–1.12 (1H, m, H_2_-8b), 1.16 (3H, s, H_3_-18), 0.94 (3H, d, *J* = 7.0 Hz, H_3_-17), 0.75 (3H, d, *J* = 7.0 Hz, H_3_-16); Exchangeable ^1^H signals observed: ^1^H NMR (DMSO-*d*_6_, 400 MHz) *δ* 9.33 (2H, br s, NH_2_-23), 8.79 (4H, br s, NH_2_-27, NH_2_-32), 7.90 (3H, br s, NH_3_-36);^13^C NMR (CD_3_OD, 100 MHz) *δ* 219.3 (C-3), 166.6 (C-21), 141.0 (C-19), 116.7 (C-20a), 75.3 (C-11), 73.1 (C-14), 59.0 (C-4), 49.0 (C-22), 48.1 (C-24^a^, C-26^a^), 46.7 (C-28^a^), 45.7 (C-9), 45.60 (C-31^a^), 45.55 (C-33^a^), 45.3 (C-12), 43.1 (C-5), 37.9 (C-10), 37.8 (C-6), 37.7 (C-35^a^), 35.2 (C-2), 31.4 (C-8a), 28.1 (C-7a), 28.0 (C-18), 25.7 (C-1a), 25.3 (C-25^b^), 24.1 (C-34^b^), 23.9 (C-29, C-30), 17.0 (C-16), 15.1 (C-15), 11.7 (C-17); (+)-HRESIMS *m*/*z* 563.4528 [M+H]^+^ (calcd for C_32_H_59_N_4_O_4_, 563.45308). ^a,b^ Interchangeable assignments that could not be distinguished. 

#### 2.2.16. 6-(3-Phenylpropanamido)hexane-1-aminium-2,2,2-trifluroacetate (**36**)

Following variant 1 of general procedure C, the reaction of 3-phenylpropanoic acid (**32**) (60 mg, 0.39 mmol), EDC·HCl (98 mg, 0.51 mmol), DMAP (68 mg, 0.56 mmol), and *tert*-butyl (6-aminohexyl)carbamate (**24**) (100 mg, 0.47 mmol). Purification was achieved by silica gel flash column chromatography (10% MeOH/CH_2_Cl_2_), affording a Boc-protected intermediate as an off-white solid (93 mg, 68%). Following general procedure B, a sub-sample of this intermediate (89 mg, 0.26 mmol) was reacted with TFA (0.2 mL) in CH_2_Cl_2_ (2 mL) to afford the mono-TFA salt of **36** as a white solid (89 mg, 94%). R*_f_* = 0.67 (RP-18, 7:3, MeOH:10% *aq*. HCl); m.p 108–110 °C; IR (ATR) ν_max_ 3292, 3062, 2934, 2863, 1686, 1659, 1629, 1202, 1170, 1134 cm^−1^; ^1^H NMR (CD_3_OD, 400 MHz) *δ* 7.27–7.23 (2H, m, H-2), 7.20–7.14 (3H, m, H-1, H-3), 3.12 (2H, dt, *J* = 7.0, 7.0 Hz, H_2_-9), 2.90 (2H, t, *J* = 7.6 Hz, H_2_-6), 2.90 (2H, dt, *J* = 7.6, 7.6 Hz, H_2_-14), 2.47 (2H, t, *J* = 7.6 Hz, H_2_-5), 1.66–1.59 (2H, m, H_2_-10), 1.47–1.32 (4H, m, H_2_-11, H_2_-13), 1.29–1.22 (2H, m, H_2_-12); Exchangeable ^1^H signals observed: ^1^H NMR (DMSO-*d*_6_, 400 MHz) *δ* 7.69 (1H, t, *J* = 5.4 Hz, NH-8), 7.64 (3H, br s, NH_3_-15); ^13^C NMR (CD_3_OD, 100 MHz) *δ* 175.1 (C-7), 142.1 (C-4), 129.42 (C-2^a^), 129.41 (C-3^a^), 127.2 (C-1), 40.6 (C-9), 40.0 (C-14), 38.9 (C-6), 32.9 (C-5), 30.1 (C-13), 28.4 (C-10), 27.2 (C-11), 27.0 (C-12); (+)-HRESIMS *m/z* 249.1961 [M+H]^+^ (calcd for C_15_H_25_N_2_O, 249.1961). ^a^ Interchangeable assignments that could not be distinguished.

#### 2.2.17. 6-(3,3-Diphenylpropanamido)hexan-1-aminium 2,2,2-trifluoroacetate (**37**)

Following variant 1 of general procedure C, 3,3-diphenylpropanoic acid (**33**) (130 mg, 0.50 mmol) was reacted with EDC·HCl (120 mg, 0.65 mmol), DMAP (92 mg, 0.75 mmol) and *tert*-butyl (6-aminohexyl)carbamate (**24**) (130 mg, 0.60 mmol). Purification was achieved by silica gel flash column chromatography (10% MeOH/CH_2_Cl_2_), affording a Boc-protected intermediate as an off-white solid (140 mg, 65%). Following general procedure B, a sub-sample of this intermediate (110 mg, 0.26 mmol) was reacted with TFA (0.2 mL) in CH_2_Cl_2_ (2 mL) to afford the mono-TFA salt of **37** as a colourless oil (101 mg, 89%). R*_f_* = 0.47 (RP-18, 7:3, MeOH:10% *aq*. HCl); IR (ATR) ν_max_ 3293, 3029, 2934, 2864, 1673, 1636, 1553, 1174, 1110, 1128 cm^−1^; ^1^H NMR (CD_3_OD, 400 MHz) *δ* 7.27–7.26 (8H, m, H-2, H-3), 7.19–7.14 (2H, m, H-1), 4.51 (1H, t, *J* = 8.2 Hz, H-5), 3.04 (2H, dt, *J* = 6.8, 6.8 Hz, H_2_-9), 2.90 (2H, d, *J* = 8.2 Hz, H_2_-6), 2.85 (2H, dt, *J* = 7.6, 7.6 Hz, H_2_-14), 1.56 (2H, qnt, *J* = 7.7 Hz, H_2_-13), 1.33–1.23 (4H, m, H_2_-10, H_2_-11), 1.12–1.05 (2H, m, H_2_-12); Exchangeable ^1^H signals observed: ^1^H NMR (DMSO-*d*_6_, 400 MHz) *δ* 7.82 (1H, t, *J* = 5.6 Hz, NH-8), 7.65 (3H, br s, NH_3_-15); ^13^C NMR (CD_3_OD, 100 MHz) *δ* 174.0 (C-7), 145.1 (C-4), 129.5 (C-2), 128.9 (C-3), 127.5 (C-1), 48.7 (C-5), 43.4 (C-6), 40.6 (C-14), 39.8 (C-9), 30.1 (C-10), 28.4 (C-13^a^), 26.92 (C-12^a^), 26.86 (C-11); (+)-HRESIMS *m/z* 325.2269 [M+H]^+^ (calcd for C_21_H_29_N_2_O, 325.2274). ^a^ Interchangeable assignments that could not be distinguished. 

#### 2.2.18. 6-(3,3,3-Triphenylpropanamido)hexane-1-aminium-2,2,2-trifluroacetate (**38**)

Following variant 1 of general procedure C, 3,3,3-triphenylpropanoic acid (**34**) (150 mg, 0.5 mmol) was reacted with EDC·HCl (120 mg, 0.65 mmol), DMAP (92 mg, 0.75 mmol) and *tert*-butyl (6-aminohexyl)carbamate (**24**) (130 mg, 0.60 mmol). Purification was achieved by silica gel column chromatography (10% MeOH/CH_2_Cl_2_), affording a Boc-protected intermediate as a white solid (200 mg, 80%). Following general procedure B, a sub-sample of this intermediate (170 mg, 0.34 mmol) was reacted with TFA (0.2 mL) in CH_2_Cl_2_ (2 mL) to afford the mono-TFA salt of **38** as a white solid (174 mg, 99%). R*_f_* = 0.21 (RP-18, 7:3, MeOH:10% *aq*. HCl); m.p 153–154 °C; IR (ATR) ν_max_ 3272, 3059, 2939, 2865, 1669, 1631, 1546, 1200, 1136, 1177 cm^−1^; ^1^H NMR (CD_3_OD, 400 MHz) *δ* 7.30–7.21 (12H, m, H-2, H-3), 7.19–7.15 (3H, m, H-1), 3.61 (2H, s, H_2_-6), 2.86 (2H, dt, *J* = 7.5, 7.5 Hz, H_2_-9), 2.85 (2H, dt, *J* = 5.6, 5.6 Hz, H_2_-14), 1.57 (2H, quin, *J* = 7.7 Hz, H_2_-13), 1.32–1.24 (2H, m, H_2_-11), 1.22–1.06 (4H, m, H_2_-10, H_2_-12); Exchangeable ^1^H signals observed: ^1^H NMR (DMSO-*d*_6_, 400 MHz) *δ* 7.63 (3H, br s, NH_3_-15); 7.49 (1H, t, *J* = 5.6 Hz, NH-8); ^13^C NMR (CD_3_OD, 100 MHz) *δ* 172.9 (C-7), 148.3 (C-4), 130.6 (C-2), 128.6 (C-3), 127.1 (C-1), 57.6 (C-5), 48.2 (C-6), 40.6 (C-14), 39.8 (C-9), 29.9 (C-10), 28.4 (C-13), 27.1 (C-12), 26.8 (C-11); (+)-HRESIMS *m*/*z* 401.2589 [M+H]^+^ (calcd for C_27_H_33_N_2_O, 401.2587).

#### 2.2.19. (3a*R*,4*R*,5*R*,7*S*,8*S*,9*R*,9a*S*,12*R*)-8-Hydroxy-4,7,9,12-tetramethyl-3-oxo-7-vinyldecahydro-4,9a-propanocyclopenta[8]annulen-5-yl (6-(3-phenylpropanamido)hexyl)glycinate (**39**)

Following general procedure A, pleuromutilin 22-*O*-tosylate (**3**) (60 mg, 0.11 mmol) was preincubated with KI (20 mg, 0.12 mmol) and then reacted with 6-(3-phenylpropanamido)hexane-1-aminium-2,2,2-trifluroacetate (**36**) (85 mg, 0.16 mmol) and DIPEA (0.17 mL, 0.96 mmol). Purification was achieved by silica gel column chromatography (0–10% MeOH/EtOAc) to afford **39** as a pale yellow oil (31 mg, 46%). R*_f_* = 0.38 (1:9, MeOH:EtOAc); ${\left[\alpha \right]}_{D}^{20.8}$ = +127.8 (*c* = 0.120, CH_2_Cl_2_); IR (ATR) ν_max_ 3111, 2927, 2859, 1730, 1646, 1542, 1455, 1260, 1095, 1023, 911, 799, 729, 699 cm^−1^; ^1^H NMR (CDCl_3_, 400 MHz) *δ* 7.29–7.25 (2H, m, H-36), 7.20–7.18 (3H, m, H-35, H-37), 6.49 (1H, dd, *J* = 17.4, 11.0 Hz, H-19), 5.79 (1H, d, *J* = 8.5 Hz, H-14), 5.43 (1H, t, *J* = 6.5 Hz, H-30), 5.34 (1H, dd, *J* = 11.0, 1.4 Hz, H_2_-20a), 5.20 (1H, dd, *J* = 17.4, 1.5 Hz, H_2_-20b), 3.36 (1H, d, *J* = 6.6 Hz, H-11), 3.35 (2H, ABq, Δδ_AB_ = 0.09, *J*_AB_ = 17.5 Hz, H_2_-22), 3.18 (2H, dt, *J* = 6.9, 6.9 Hz, H_2_-29), 2.95 (2H, t, *J* = 7.7 Hz, H_2_-33), 2.68–2.54 (2H, m, H_2_-24), 2.45 (2H, t, *J* = 7.7 Hz, H_2_-32), 2.37–2.30 (2H, m, H_2_-10), 2.27–2.19 (2H, m, H_2_-2), 2.10 (1H, br s, H-4), 2.10–2.01 (1H, m, H_2_-13a), 1.79–1.75 (1H, m, H_2_-8a), 1.69–1.61 (2H, m, H_2_-1a, H-6), 1.56–1.45 (4H, m, H_2_-1b, H_2_-7b, H_2_-25), 1.44 (3H, s, H_3_-15), 1.43–1.37 (2H, m, H_2_-28), 1.32–1.23 (6H, m, H_2_-7a, H_2_-13b, H_2_-26, H_2_-27), 1.16 (3H, s, H_3_-18), 1.13–1.09 (1H, m, H_2_-8b), 0.88 (3H, d, *J* = 7.0 Hz, H_3_-17), 0.71 (3H, d, *J* = 7.0 Hz, H_3_-16); ^13^C NMR (CDCl_3_, 100 MHz) *δ* 217.1 (C-3), 172.2 (C-31), 170.7 (C-21), 141.0 (C-34), 139.2 (C-19), 128.6 (C-35), 128.5 (C-36), 126.4 (C-37), 117.4 (C-20), 74.7 (C-11), 69.1 (C-14), 58.3 (C-4), 51.3 (C-22), 49.4 (C-24), 45.6 (C-9), 45.1 (C-13), 44.1 (C-12), 41.9 (C-5), 39.4 (C-29), 38.7 (C-32), 36.8 (C-6), 36.2 (C-10), 34.6 (C-2), 31.9 (C-33), 30.6 (C-8), 29.5 (C-28), 29.4 (C-25), 27.0 (C-7), 26.7 (C-26), 26.6 (C-18), 26.5 (C-27), 25.0 (C-1), 16.8 (C-16), 15.0 (C-15), 11.6 (C-17); (+)-HRESIMS *m*/*z* 609.4260 [M+H]^+^ (calcd for C_37_H_57_N_2_O_5_, 609.4262). 

#### 2.2.20. (3a*R*,4*R*,5*R*,7*S*,8*S*,9*R*,9a*S*,12*R*)-8-Hydroxy-4,7,9,12-tetramethyl-3-oxo-7-vinyldecahydro-4,9a-propanocyclopenta[8]annulen-5-yl (6-(3,3-diphenylpropanamido)hexyl)glycinate (**40**)

Following general procedure A, pleuromutilin 22-*O*-tosylate (**3**) (80 mg, 0.15 mmol) was preincubated with KI (28 mg, 0.17 mmol) and then reacted with 6-(3,3-diphenylpropanamido)hexane-1-aminium-2,2,2-trifluroacetate (**37**) (100 mg, 0.23 mmol) and DIPEA (0.24 mL, 1.4 mmol). Purification was achieved by silica gel column chromatography (70–100% EtOAc/hexane) to afford **40** as a colourless oil (58 mg, 56%). R*_f_* = 0.53 (100% EtOAc); ${\left[\alpha \right]}_{D}^{20.6}$ = +108.3 (*c* = 0.100, CH_2_Cl_2_); IR (ATR) ν_max_ 3318, 2932, 2861, 1729, 1646, 1549, 1453, 1215, 1152, 910, 728, 699 cm^−1^; ^1^H NMR (CDCl_3_, 400 MHz) *δ* 7.31–7.24 (8H, m, H-35, H-36), 7.22–7.18 (2H, m, H-37), 6.54 (1H, dd, *J* = 17.4, 11.0 Hz, H-19), 5.81 (1H, d, *J* = 8.4 Hz, H-14), 5.37 (1H, dd, *J* = 10.9, 1.4 Hz, H_2_-20a), 5.37 (1H, br s, H-30), 5.22 (1H, dd, *J* = 17.4, 1.5 Hz, H_2_-20b), 4.58 (1H, t, *J* = 7.8 Hz, H-33), 3.39 (1H, d, *J* = 6.4 Hz, H-11), 3.31 (2H, ABq, Δδ_AB_ = 0.08, *J*_AB_ = 17.5 Hz, H_2_-22), 3.09 (2H, dt, *J* = 6.6, 6.6 Hz, H_2_-29), 2.89 (2H, d, *J* = 7.8 Hz, H_2_-32), 2.62–2.48 (2H, m, H_2_-24), 2.42–2.35 (1H, m, H-10), 2.33–2.16 (2H, m, H_2_-2), 2.13 (1H, br s, H-4), 2.11–2.06 (1H, m, H_2_-13a), 1.82–1.78 (1H, m, H_2_-8a), 1.72–1.64 (2H, m, H_2_-1a, H-6), 1.61–1.51 (1H, m, H_2_-7b), 1.50–1.45 (1H, m, H_2_-1b), 1.48 (3H, s, H_3_-15), 1.45–1.24 (8H, m, H_2_-7a, H_2_-13b, H_2_-25, H_2_-27, H_2_-28), 1.22–1.15 (1H, m, H_2_-8b), 1.19 (3H, s, H_3_-18), 1.13–1.07 (2H, m, H_2_-26), 0.91 (3H, d, *J* = 7.0 Hz, H_3_-17), 0.75 (3H, d, *J* = 6.9 Hz, H_3_-16); ^13^C NMR (CDCl_3_, 100 MHz) *δ* 217.2 (C-3), 171.4 (C-21), 171.1 (C-31), 143.8 (C-34), 139.2 (C-19), 128.6 (C-36), 127.8 (C-35), 126.6 (C-37), 117.2 (C-20), 74.7 (C-11), 68.7 (C-14), 58.3 (C-4), 51.7 (C-22), 49.5 (C-24), 47.6 (C-33), 45.5 (C-9), 45.1 (C-13), 44.1 (C-12), 43.6 (C-32), 41.9 (C-5), 39.4 (C-29), 36.8 (C-6), 36.1 (C-10), 34.6 (C-2), 30.5 (C-8), 29.9 (C-25), 29.4 (C-28), 27.0 (C-7), 26.8 (C-26), 26.6 (C-18), 26.5 (C-27), 24.9 (C-1), 16.8 (C-16), 15.0 (C-15), 11.6 (C-17); (+)-HRESIMS *m*/*z* 685.4576 [M+H]^+^ (calcd for C_43_H_61_N_2_O_5_, 685.4575).

#### 2.2.21. (3a*R*,4*R*,5*R*,7*S*,8*S*,9*R*,9a*S*,12*R*)-8-Hydroxy-4,7,9,12-tetramethyl-3-oxo-7-vinyldecahydro-4,9a-propanocyclopenta[8]annulen-5-yl(6-(3,3,3-triphenylpropanamido)hexyl)glycinate (**41**)

Following general procedure A, pleuromutilin 22-*O*-tosylate (**3**) (100 mg, 0.19 mmol) was preincubated with KI (35 mg, 0.21 mmol) and then reacted with 6-(3,3,3-triphenylpropanamido)hexane-1-aminium-2,2,2-triflurocetate (**38**) (150 mg, 0.29 mmol) and DIPEA (0.30 mL, 1.7 mmol). Purification was achieved by silica gel column chromatography (50–100% EtOAc/hexane) to afford **41** as a colourless oil (45 mg, 40%). R*_f_* = 0.50 (100% EtOAc); ${\left[\alpha \right]}_{D}^{20.4}$ = +123.3 (*c* = 0.100, CH_2_Cl_2_); IR (ATR) ν_max_ 3331, 2931, 2861, 1729, 1647, 1447, 1215, 1152, 909, 728, 700 cm^−1^; ^1^H NMR (CDCl_3_, 400 MHz) *δ* 7.30–7.25 (12H, m, H-35, H-36), 7.22–7.18 (3H, m, H-37), 6.50 (1H, dd, *J* = 17.4, 11.0 Hz, H-19), 5.77 (1H, d, *J* = 8.5 Hz, H-14), 5.33 (1H, dd, *J* = 11.0, 1.4 Hz, H_2_-20a), 5.19 (1H, dd, *J* = 17.4, 1.4 Hz, H_2_-20b), 4.74 (1H, br t, *J* = 5.1 Hz, H-30), 3.54 (2H, s, H_2_-32), 3.35 (1H, d, *J* = 6.8 Hz, H-11), 3.27 (2H, ABq, Δδ_AB_ = 0.08, *J*_AB_ = 17.6 Hz, H_2_-22), 2.89 (2H, br dt, *J* = 6.4, 6.4 Hz, H_2_-29), 2.57–2.43 (2H, m, H_2_-24), 2.38–2.31 (1H, m, H-10), 2.29–2.13 (2H, m, H_2_-2), 2.09 (1H, s, H-4), 2.09–2.03 (1H, m, H_2_-13a), 1.79–1.74 (1H, m, H_2_-8b), 1.69–1.60 (2H, m, H_2_-1a, H-6), 1.60–1.53 (1H, m, H_2_-7b), 1.48–1.40 (1H, m, H_2_-1b), 1.44 (3H, s, H_3_-15), 1.38–1.24 (4H, m, H_2_-7a, H_2_-13b, H_2_-25), 1.18–1.12 (1H, m, H_2_-8b), 1.15 (3H, s, H_3_-18), 1.06–0.94 (6H, m, H_2_-26, H_2_-27, H_2_-28), 0.87 (3H, d, *J* = 7.0 Hz, H_3_-17), 0.71 (3H, d, *J* = 6.9 Hz, H_3_-16); ^13^C NMR (CDCl_3_, 100 MHz) *δ* 217.2 (C-3), 171.5 (C-21), 170.6 (C-31), 146.4 (C-34), 139.2 (C-19), 129.3 (C-35), 128.2 (C-36), 126.6 (C-37), 117.3 (C-20), 74.7 (C-11), 68.7 (C-14), 58.3 (C-4), 56.3 (C-33), 51.8 (C-22), 49.6 (C-24), 49.0 (C-32), 45.6 (C-9), 45.1 (C-13), 44.1 (C-12), 41.9 (C-5), 39.4 (C-29), 36.8 (C-6), 36.2 (C-10), 34.6 (C-2), 30.6 (C-8), 29.9 (C-28), 29.0 (C-27), 27.0 (C-7), 26.9 (C-26), 26.7 (C-25), 26.5 (C-18), 25.0 (C-1), 16.8 (C-16), 15.0 (C-15), 11.6 (C-17); (+)-HRESIMS *m*/*z* 761.4891 [M+H]^+^ (calcd for C_49_H_65_N_2_O_5_, 761.4888).

#### 2.2.22. *N*^1^-(2-(((3a*R*,4*R*,5*R*,7*S*,8*S*,9*R*,9a*S*,12*R*)-8-Hydroxy-4,7,9,12-tetramethyl-3-oxo-7-vinyldecahydro-4,9a-propanocyclopenta[8]annulen-5-yl)oxy)-2-oxoethyl)-*N*3-(7-(3-phenylpropanamido)heptyl)propane-1,3-diaminium 2,2,2-trifluoroacetate (**42**)

Following variant 2 of general procedure C, **29** (73 mg, 0.082 mmol) was reacted with 3-phenylpropanoic acid (**32**) (11 mg, 0.075 mmol), HOBt (36 mg, 0.27 mmol), HBTU (37 mg, 0.089 mmol), and DIPEA (0.13 mL, 0.75 mmol). Purification was achieved by C_8_ reversed-phase column chromatography 0%–100% MeOH/H_2_O (0.05% TFA) to afford the di-TFA salt of **42** as a beige crystalline solid (42 mg, 51%). R*_f_* = 0.76 (RP-18, 9:1, MeOH: 10% *aq*. HCl); ${\left[\alpha \right]}_{D}^{18.4}$ = +162.9 (*c* = 0.104, MeOH); m.p 140–142 °C; IR (ATR) ν_max_ 3390, 3279, 2931, 2861, 1751, 1724, 1673, 1596, 1197, 1171 cm^−1^; ^1^H NMR (CD_3_OD, 400 MHz) *δ* 7.27–7.23 (2H, m, H-41), 7.20–7.14 (3H, m, H-40, H-42), 6.31 (1H, dd, *J* = 18.0, 10.7 Hz, H-19), 5.84 (1H, d, *J* = 8.4 Hz, H-14), 5.19 (1H, dd, *J* = 5.9, 1.4 Hz, H_2_-20a), 5.16 (1H, s, H_2_-20b), 3.95 (2H, ABq, Δδ_AB_ = 014, *J*_AB_ = 17.1 Hz, H_2_-22), 3.51 (1H, d, *J* = 6.0 Hz, H-11), 3.20–3.15 (2H, m, H_2_-26), 3.11 (4H, dt, *J* = 7.0, 7.0 Hz, H_2_-24, H_2_-34), 3.00 (2H, t, *J* = 7.9 Hz, H_2_-28), 2.90 (2H, t, *J* = 7.6 Hz, H_2_-38), 2.46 (2H, t, *J* = 7.6 Hz, H_2_-37), 2.39 (1H, br s, H-4), 2.32–2.27 (2H, m, H-10, H_2_-13a), 2.24–2.11 (4H, m, H_2_-2, H_2_-25), 1.84–1.79 (1H, m, H_2_-8a), 1.71–1.64 (3H, m, H_2_-1a, H-6, H_2_-7b), 1.56–1.52 (2H, m, H_2_-29^a^), 1.49–1.44 (1H, m, H_2_-1b), 1.46 (3H, s, H_3_-15), 1.42–1.34 (8H, m, H_2_-7a, H_2_-13b, H_2_-30^a^, H_2_-31^a^, H_2_-33^a^), 1.28–1.25 (2H, m, H_2_-32^a^), 1.19–1.12 (1H, m, H_2_-8b), 1.16 (3H, s, H_3_-18), 0.94 (3H, d, *J* = 7.0 Hz, H_3_-17), 0.75 (3H, d, *J* = 7.0 Hz, H_3_-16); Exchangeable ^1^H signals observed: ^1^H NMR (DMSO-*d*_6_, 400 MHz) *δ* 9.20 (2H, br s, NH_2_-23), 8.48 (2H, br s, NH_2_-27), 5.16 (1H, t, *J* = 5.4 Hz, NH-35); ^13^C NMR (CD_3_OD, 100 MHz) *δ* 219.3 (C-3), 175.1 (C-36), 166.6 (C-21), 142.1 (C-39), 141.0 (C-19), 129.40 (C-40), 129.37 (C-41), 127.2 (C-42), 116.8 (C-20), 75.3 (C-11), 73.1 (C-14), 59.0 (C-4), 49.0 (C-22, C-28), 46.7 (C-9), 45.6 (C-13, C-24, C-26), 45.4 (C-12), 43.1 (C-5), 40.1 (C-34^b^), 38.9 (C-3), 37.9 (C-10), 37.7 (C-6), 35.2 (C-2), 32.9 (C-38), 31.4 (C-8), 30.1 (C-29^b^), 29.6 (C-30^b^), 28.1 (C-18), 28.0 (C-31^b^), 27.5 (C-32^b^), 27.3 (C-33^b^), 27.1 (C-7), 25.7 (C-1), 23.9 (C-25), 17.0 (C-16), 15.1 (C-15), 11.7 (C-17); (+)-HRESIMS *m*/*z* 680.4997 [M+H]^+^ (calcd for C_41_H_66_N_3_O_5_, 680.4997). ^a,b^ Interchangeable assignments that could not be distinguished.

#### 2.2.23. *N*^1^-(7-(3,3-diphenylpropanamido)heptyl)-*N*3-(2-(((3a*R*,4*R*,5*R*,7*S*,8*S*,9*R*,9a*S*,12*R*)-8-hydroxy-4,7,9,12-tetramethyl-3-oxo-7-vinyldecahydro-4,9a-propanocyclopenta[8]annulen-5-yl)oxy)-2-oxoethyl)propane-1,3-diaminium 2,2,2-trifluoroacetate (**43**)

Following variant 2 of general procedure C, **29** (58 mg, 0.065 mmol) was reacted with 3,3-diphenylpropanoic acid (**33**) (14 mg, 0.059 mmol), HOBt (28 mg, 0.21 mmol), HBTU (27 mg, 0.071 mmol), and DIPEA (0.10 mL, 0.59 mmol). Purification was achieved by C_8_ reversed-phase column chromatography 0%–100% MeOH/H_2_O (0.05% TFA) to afford the di-TFA salt of **43** as a pale yellow crystalline solid (48 mg, 75%). R*_f_* = 0.71 (RP-18, 9:1, MeOH:10% *aq*. HCl); ${\left[\alpha \right]}_{D}^{18.6}$ = +106.9 (*c* = 0.103, MeOH); m.p 98–101 °C; IR (ATR) ν_max_ 3389, 3259, 2931, 2861, 1751, 1724, 1673, 1417, 1197, 1171 cm^−1^; ^1^H NMR (CD_3_OD, 400 MHz) *δ* 7.26–7.25 (8H, m, H-40, H-41), 7.19–7.14 (2H, m, H-42), 6.32 (1H, dd, *J* = 17.2, 11.7 Hz, H-19), 5.85 (1H, d, *J* = 8.4 Hz, H-14), 5.19 (1H, dd, *J* = 6.8, 1.4 Hz, H_2_-20a), 5.16 (1H, s, H_2_-20b), 4.51 (1H, t, *J* = 8.2 Hz, H-38), 3.94 (2H, ABq, Δδ_AB_ = 0.13, *J*_AB_ = 17.1 Hz, H_2_-22), 3.51 (1H, d, *J* = 6.0 Hz, H-11), 3.20–3.09 (4H, m, H_2_-24, H_2_-26), 3.02 (2H, t, *J* = 6.9 Hz, H_2_-34), 2.98 (2H, t, *J* = 7.9 Hz, H_2_-28), 2.90 (2H, d, *J* = 8.1 Hz, H_2_-37), 2.40 (1H, br s, H-4), 2.33–2.27 (1H, m, H-10), 2.24–2.20 (2H, m, H_2_-2), 2.18–2.09 (3H, m, H_2_-13a, H_2_-25), 1.83–1.79 (1H, m, H_2_-8a), 1.74–1.61 (5H, m, H_2_-1a, H-6, H_2_-7b, H_2_-33^a^), 1.56–1.53 (1H, m, H_2_-1b), 1.46 (3H, s, H_3_-15), 1.43–1.39 (1H, m, H_2_-13b), 1.36–1.34 (1H, m, H_2_-7a), 1.33–1.22 (6H, m, H_2_-29^a^, H_2_-30^a^, H_2_-31^a^), 1.20–1.15 (1H, m, H_2_-8b), 1.16 (3H, s, H_3_-18), 1.13–1.07 (2H, m, H_2_-32^a^), 0.94 (3H, d, *J* = 7.0 Hz, H_3_-17), 0.75 (3H, d, *J* = 7.0 Hz, H_3_-16); Exchangeable ^1^H signals observed: ^1^H NMR (DMSO-*d*_6_, 400 MHz) *δ* 9.38 (2H, br s, NH-23), 8.68 (2H, br s, NH-27), 7.82 (1H, t, *J* = 5.6 Hz, NH-35); ^13^C NMR (CD_3_OD, 100 MHz) *δ* 219.3 (C-3), 173.9 (C-36), 166.7 (C-21), 145.1 (C-39), 141.0 (C-19), 129.5 (C-40^b^), 128.9 (C-41^b^), 127.4 (C-42), 116.8 (C-20), 75.3 (C-11), 73.1 (C-14), 59.0 (C-4), 49.1 (C-22, C-28, C-38), 46.7 (C-9), 45.6 (C-13, C-24, C-26), 45.4 (C-12), 43.4 (C-37), 43.2 (C-5), 40.0 (C-34), 37.9 (C-10), 37.8 (C-6), 35.2 (C-2), 31.4 (C-8), 30.1 (C-29^c^), 29.6 (C-30^c^), 28.2 (C-18), 28 (C-31^c^), 27.3 (C-32^c^, C-33^c^), 27.1 (C-7), 25.8 (C-1), 23.9 (C-25), 17.0 (C-16), 15.1 (C-15), 11.8 (C-17); (+)-HRESIMS *m*/*z* 778.5134 [M+Na]^+^ (calcd for C_47_H_69_N_3_O_5_Na, 778.5129). ^a,b,c^ Interchangeable assignments that could not be distinguished.

#### 2.2.24. *N*^1^-(2-(((3a*R*,4*R*,5*R*,7*S*,8*S*,9*R*,9a*S*,12*R*)-8-Hydroxy-4,7,9,12-tetramethyl-3-oxo-7-vinyldecahydro-4,9a-propanocyclopenta[8]annulen-5-yl)oxy)-2-oxoethyl)-*N*3-(7-(2,2,2-triphenylacetamido)heptyl)propane-1,3-diaminium 2,2,2-trifluoroacetate (**44**)

Following variant 2 of general procedure C, **29** (85 mg, 0.096 mmol) was reacted with 2,2,2-triphenylacetic acid (**35**) (26 mg, 0.090 mmol), HOBt (45 mg, 0.33 mmol), HBTU (38 mg, 0.10 mmol), and DIPEA (0.15 mL, 0.87 mmol). Purification was achieved by C_8_ reversed-phase column chromatography 0%–100% MeOH/H_2_O (0.05% TFA) to afford the di-TFA salt of **44** as a beige crystalline solid (47 mg, 46%). R*_f_* = 0.62 (RP-18, 9:1, MeOH:10% *aq*. HCl); ${\left[\alpha \right]}_{D}^{18.8}$ = +81.0 (*c* = 0.103, MeOH); m.p 197–199 °C; IR (ATR) ν_max_ 3440, 3237, 2926, 2862, 1735, 1707, 1668, 1645, 1200, 1127 cm^−1^; ^1^H NMR (CD_3_OD, 400 MHz) *δ* 7.32–7.23 (15H, m, H-39, H-40, H-41), 6.32 (1H, dd, *J* = 17.2, 11.5 Hz, H-19), 5.85 (1H, d, *J* = 8.4 Hz, H-14), 5.19 (1H, dd, *J* = 7.0, 1.4 Hz, H_2_-20a), 5.16 (1H, s, H_2_-20b), 3.93 (2H, ABq, Δδ_AB_ = 0.13, *J*_AB_ = 17.2 Hz, H_2_-22), 3.51 (1H, d, *J* = 6.1 Hz, H-11), 3.25 (2H, t, *J* = 7.0 Hz, H_2_-34), 3.18–3.07 (4H, m, H_2_-26, H_2_-28), 2.99 (2H, dt, *J* = 7.8, 7.8 Hz, H_2_-24), 2.40 (1H, br s, H-4), 2.34–2.27 (1H, m, H-10), 2.24–2.20 (1H, m, H_2_-13a), 2.19–2.15 (2H, m, H_2_-2), 2.13–2.07 (2H, m, H_2_-25), 1.84–1.80 (1H, m, H_2_-8a), 1.69–1.62 (3H, m, H-1a, H_2_-6, H_2_-7b), 1.62–1.48 (3H, m, H_2_-1b, H_2_-33), 1.46 (3H, s, H_3_-15), 1.44–1.38 (2H, m, H_2_-7a, H_2_-13b), 1.37–1.24 (6H, m, H_2_-29, H_2_-30, H_2_-32), 1.24–1.19 (2H, m, H_2_-31), 1.17 (3H, s, H_3_-18), 1.13–1.12 (1H, m, H_2_-8b), 0.94 (3H, d, *J* = 7.0 Hz, H_3_-17), 0.75 (3H, d, *J* = 7.0 Hz, H_3_-16); Exchangeable ^1^H signals observed: ^1^H NMR (DMSO-*d*_6_, 400 MHz) *δ* 9.21 (2H, br s, NH_2_-23), 8.47 (2H, br s, NH_2_-27), 7.08 (1H, t, *J* = 5.6 Hz, NH-35); ^13^C NMR (CD_3_OD, 100 MHz) *δ* 219.2 (C-3), 175.6 (C-36), 166.8 (C-21), 144.9 (C-38), 141.1 (C-19), 131.6 (C-39), 128.9 (C-40), 128.0 (C-41), 116.8 (C-20), 75.3 (C-11), 73.2 (C-14), 69.3 (C-37), 59.0 (C-4), 49.1 (C-22, C-24, C-28), 46.7 (C-9), 45.7 (C-13), 45.7 (C-26), 45.4 (C-12), 43.2 (C-5), 40.9 (C-34), 37.9 (C-10), 37.8 (C-6), 35.2 (C-2), 31.4 (C-8), 30.0 (C-30), 29.6 (C-31), 28.2 (C-18), 28.0 (C-33), 27.6 (C-29), 27.3 (C-32), 27.1 (C-7), 25.8 (C-1), 24.0 (C-25), 17.0 (C-16), 15.2 (C-15), 11.8 (C-17); (+)-HRESIMS *m/z* 832.5622 [M+H]^+^ (calcd for C_53_H_74_N_3_O_5_, 832.5623).

#### 2.2.25. *N*^1^-(2-(((3a*R*,4*R*,5*R*,7*S*,8*S*,9*R*,9a*S*,12*R*)-8-Hydroxy-4,7,9,12-tetramethyl-3-oxo-7-vinyldecahydro-4,9a-propanocyclopenta[8]annulen-5-yl)oxy)-2-oxoethyl)-*N*3-(10-(3-phenylpropanamido)decyl)propane-1,3-diaminium 2,2,2-trifluoroacetate (**45**)

Following variant 2 of general procedure C, **30** (85 mg, 0.091 mmol) was reacted with 3-phenylpropanoic acid (**32**) (13 mg, 0.083 mmol), HOBt (41 mg, 0.30 mmol), HBTU (40 mg, 0.10 mmol), and DIPEA (0.14 mL, 0.83 mmol). Purification was achieved by C_8_ reversed-phase column chromatography 0%–100% MeOH/H_2_O (0.05% TFA) to afford the di-TFA salt of **45** as a pale-yellow crystalline solid (58 mg, 72%). R*_f_* = 0.74 (RP-18, 9:1, MeOH:10% *aq*. HCl); ${\left[\alpha \right]}_{D}^{19.0}$ = +56.1 (*c* = 0.101, MeOH); m.p 118–120 °C; IR (ATR) ν_max_ 3389, 3256, 3088, 2929, 2859, 1737, 1672, 1635, 1198, 1128 cm^−1^; ^1^H NMR (CD_3_OD, 400 MHz) *δ* 7.27–7.23 (2H, m, H-43), 7.20–2.17 (1H, m, H-44), 7.20–7.14 (2H, m, H-45), 6.31 (1H, dd, *J* = 17.2, 11.7 Hz, H-19), 5.84 (1H, d, *J* = 8.4 Hz, H-14), 5.19 (1H, dd, *J* = 6.9, 1.5 Hz, H_2_-20a), 5.15 (1H, s, H_2_-20b), 3.94 (2H, ABq, Δδ_AB_ = 0.14, *J*_AB_ = 17.1 Hz, H_2_-22), 3.51 (1H, d, *J* = 6.0 Hz, H-11), 3.20–3.14 (2H, m, H_2_-26), 3.10 (4H, dt, *J* = 6.9, 6.9 Hz, H_2_-24, H_2_-37), 3.00 (2H, t, *J* = 8 Hz, H_2_-28), 2.90 (2H, t, *J* = 7.6 Hz, H_2_-41), 2.46 (2H, t, *J* = 7.7 Hz, H_2_-40), 2.39 (1H, br s, H-4), 2.33–2.27 (1H, m, H-10), 2.24–2.20 (1H, m, H_2_-13a), 2.17–2.09 (2H, m, H_2_-2), 2.09–1.74 (2H, m, H_2_-25), 1.83–1.79 (1H, m, H_2_-8a), 1.74–1.63 (1H, m, H_2_-6), 1.63–1.74 (1H, m, H-1a), 1.63–1.42 (1H, m, H_2_-7b), 1.56–1.52 (1H, m, H_2_-13b), 1.46 (3H, s, H_3_-15), 1.42–1.22 (1H, m, H_2_-30), 1.22–0.00 (2H, m, H_2_-1b), 1.22–1.42 (15H, m, H_2_-7a, H_2_-29, H_2_-31, H_2_-32, H_2_-33, H_2_-34, H_2_-35, H_2_-36), 1.19–1.11 (1H, m, H_2_-8b), 1.16 (H, s, H_3_-18), 0.94 (H, d, *J* = 7.0 Hz, H_3_-17), 0.75 (H, d, *J* = 7.0 Hz, H_3_-16); Exchangeable ^1^H signals observed: ^1^H NMR (DMSO-*d*_6_, 400 MHz) *δ* 9.19 (2H, br s, NH_2_-23), 8.45 (2H, br s, NH_2_-27), 7.75 (1H, t, *J* = 5.6 Hz, NH-38); ^13^C NMR (CD_3_OD, 100 MHz) *δ* 219.2 (C-3), 175.0 (C-39), 166.6 (C-21), 142.1 (C-42), 141.0 (C-19), 129.40 (C-43^a^), 129.38 (C-45^a^), 127.2 (C-44), 116.8 (C-20a), 75.3 (C-11), 73.1 (C-14), 59.0 (C-4), 48.6 (C-22, C-28), 46.7 (C-9), 45.6 (C-13a, C-24, C-26), 45.4 (C-12), 43.1 (C-5), 40.3 (C-37), 38.9 (C-40), 37.9 (C-10), 37.7 (C-6), 35.2 (C-2), 33.0 (C-41), 31.4 (C-8a), 30.4 (C-30^b^), 30.31 (C-31^b^), 30.29 (C-32^b^), 30.27 (C-33^b^), 30.1 (C-34^b^), 28.2 (C-29), 28.0 (C-35^b^), 27.8 (C-18), 27.4 (C-36), 27.1 (C-7a), 25.8 (C-1a), 23.9 (C-25), 17.0 (C-16), 15.2 (C-15), 11.8 (C-17); (+)-HRESIMS *m/z* 744.5303 [M+Na]^+^ (calcd for C_44_H_71_N_3_O_5_Na, 744.5286)). ^a,b^ Interchangeable assignments that could not be distinguished.

#### 2.2.26. *N*^1^-(10-(3,3-diphenylpropanamido)decyl)-*N*3-(2-(((3a*R*,4*R*,5*R*,7*S*,8*S*,9*R*,9a*S*,12*R*)-8-hydroxy-4,7,9,12-tetramethyl-3-oxo-7-vinyldecahydro-4,9a-propanocyclopenta[8]annulen-5-yl)oxy)-2-oxoethyl)propane-1,3-diaminium 2,2,2-trifluoroacetate (**46**)

Following variant 2 of general procedure C, **30** (85 mg, 0.091 mmol) was reacted with 3,3-diphenylpropanoic acid (**33**) (19 mg, 0.083 mmol), HOBt (41 mg, 0.30 mmol), HBTU (40 mg, 0.10 mmol), and DIPEA (0.14 mL, 0.83 mmol). Purification was achieved by C_8_ reversed-phase column chromatography 0%–100% MeOH/H_2_O (0.05% TFA) to afford the di-TFA salt of **46** as a beige crystalline solid (54 mg, 62%). R*_f_* = 0.68 (RP-18, 9:1, MeOH:10% *aq*. HCl); ${\left[\alpha \right]}_{D}^{19.2}$ = +40.1 (*c* = 0.100, MeOH); m.p 133–136 °C; IR (ATR) ν_max_ 3412, 3246, 3087, 2928, 2858, 1736, 1671, 1635, 1198, 1128 cm^−1^; ^1^H NMR (CD_3_OD, 400 MHz) *δ* 7.26–7.23 (8H, m, H-43, H-44), 7.18–7.13 (2H, m, H-45), 6.32 (1H, dd, *J* = 17.1, 11.8 Hz, H-19), 5.84 (1H, d, *J* = 8.4 Hz, H-14), 5.19 (1H, dd, *J* = 6.6, 1.5 Hz, H_2_-20a), 5.16 (1H, s, H_2_-20b), 4.50 (2H, t, *J* = 8.2 Hz, H_2_-41), 3.94 (2H, ABq, Δδ_AB_ = 0.14, *J*_AB_ = 17.1 Hz, H_2_-22), 3.51 (1H, d, *J* = 6.0 Hz, H-11), 3.20–3.13 (2H, m, H_2_-26), 3.11 (2H, dt, *J* = 7.8, 7.8 Hz, H_2_-24), 3.02 (2H, t, *J* = 6.8 Hz, H_2_-28), 2.99 (2H, t, *J* = 6.7 Hz, H_2_-37), 2.89 (2H, d, *J* = 8.2 Hz, H_2_-40), 2.39 (1H, br s, H-4), 2.33–2.28 (1H, m, H-10), 2.26–2.20 (2H, m, H_2_-2), 2.17–2.11 (3H, m, H_2_-13a, H_2_-25), 1.83–1.79 (1H, m, H_2_-8a), 1.74–1.62 (4H, m, H_2_-1a, H_2_-7b, H_2_-36), 1.60–1.52 (1H, m, H-6), 1.50–1.48 (1H, m, H_2_-13b), 1.46 (3H, s, H_3_-15), 1.42–1.20 (14H, m, H_2_-1b, H_2_-7a, H_2_-30, H_2_-31, H_2_-32, H_2_-33, H_2_-34, H_2_-35), 1.19–1.14 (1H, m, H_2_-8b), 1.16 (3H, s, H_3_-18), 1.12–1.05 (2H, m, H_2_-29), 0.93 (3H, d, *J* = 7.0 Hz, H_3_-17), 0.75 (3H, d, *J* = 7.0 Hz, H_3_-16); Exchangeable ^1^H signals observed: ^1^H NMR (DMSO-*d*_6_, 400 MHz) *δ* 9.21 (2H, br s, NH_2_-23), 8.48 (2H, br s, NH_2_-27), 7.79 (1H, t, *J* = 5.6 Hz, NH-38); ^13^C NMR (CD_3_OD, 100 MHz) *δ* 219.2 (C-3), 173.8 (C-39), 166.6 (C-21), 145.1 (C-42), 141.0 (C-19), 129.4 (C-44), 128.9 (C-43), 127.4 (C-45), 116.8 (C-20a), 75.2 (C-11), 73.1 (C-14), 59.0 (C-4), 49.0 (C-22, C-28, C-41), 46.7 (C-9), 45.6 (C-13a, C-24, C-26), 45.4 (C-12), 43.4 (C-40), 43.1 (C-5), 40.2 (C-37), 37.9 (C-10), 37.7 (C-6), 35.2 (C-2), 31.4 (C-8a), 30.4 (C-30^a^), 30.3 (C-31^a^), 30.2 (C-32^a^), 30.1 (C-33^a^), 28.1 (C-29), 28.0 (C-18), 27.7 (C-34^a^), 27.4 (C-35^a^, C-36^a^), 27.1 (C-7a), 25.8 (C-1a), 23.9 (C-25), 17.0 (C-16), 15.1 (C-15), 11.8 (C-17); (+)-HRESIMS *m*/*z* 798.5776 [M+H]^+^ (calcd for C_50_H_76_N_3_O_5_, 798.5779). ^a,b^ Interchangeable assignments that could not be distinguished.

#### 2.2.27. *N*^1^-(2-(((3a*R*,4*R*,5*R*,7*S*,8*S*,9*R*,9a*S*,12*R*)-8-Hydroxy-4,7,9,12-tetramethyl-3-oxo-7-vinyldecahydro-4,9a-propanocyclopenta[8]annulen-5-yl)oxy)-2-oxoethyl)-*N*3-(10-(3,3,3-triphenylpropanamido)decyl)propane-1,3-diaminium 2,2,2-trifluoroacetate (**47**)

Following variant 2 of general procedure C, **30** (85 mg, 0.091 mmol) was reacted with 3,3,3-triphenylpropanoic acid (**34**) (25 mg, 0.083 mmol), HOBt (41 mg, 0.30 mmol), HBTU (40 mg, 0.10 mmol), and DIPEA (0.14 mL, 0.83 mmol). Purification was achieved by C_8_ reversed-phase column chromatography 0%–100% MeOH/H_2_O (0.05% TFA) to afford the di-TFA salt of **47** as a beige crystalline solid (53 mg, 71%). R*_f_* = 0.56 (RP-18, 9:1, MeOH:10% *aq*. HCl); ${\left[\alpha \right]}_{D}^{19.4}$ = +46.1 (*c* = 0.101, MeOH); m.p 152–155 °C; IR (ATR) ν_max_ 3419, 3245, 3088, 2928, 2859, 1736, 1669, 1645, 1197, 1128 cm^−1^; ^1^H NMR (CD_3_OD, 400 MHz) *δ* 7.29–7.21 (12H, m, H-43, H-44), 7.18–7.14 (3H, m, H-45), 6.32 (1H, dd, *J* = 17.1, 11.6 Hz, H-19), 5.85 (1H, d, *J* = 8.4 Hz, H-14), 5.20 (1H, dd, *J* = 6.5, 1.4 Hz, H_2_-20a), 5.16 (1H, s, H_2_-20b), 3.94 (2H, ABq, Δδ_AB_ = 0.14, *J*_AB_ = 17.2 Hz, H_2_-22), 3.60 (2H, s, H_2_-40), 3.51 (1H, d, *J* = 6.0 Hz, H-11), 3.21–3.13 (2H, m, H_2_-26), 3.10 (2H, dt, *J* = 7.8, 7.8 Hz, H_2_-24), 2.99 (2H, dt, *J* = 7.9, 7.9 Hz, H_2_-28), 2.83 (2H, t, *J* = 6.8 Hz, H_2_-37), 2.39 (1H, br s, H-4), 2.32–2.27 (2H, m, H_2_-2), 2.24–2.20 (1H, m, H-10), 2.18–2.09 (3H, m, H_2_-13a, H_2_-25), 1.83–1.79 (1H, m, H_2_-8a), 1.72–1.65 (4H, m, H_2_-1a, H_2_-7b, H_2_-36), 1.60–1.53 (1H, m, H-6), 1.49–1.45 (2H, m, H_2_-1b, H_2_-13b), 1.46 (3H, s, H_3_-15), 1.39–1.19 (11H, m, H_2_-7a, H_2_-29, H_2_-30^a^, H_2_-31^a^, H_2_-32^a^, H_2_-33^a^), 1.18–1.14 (1H, m, H_2_-8b), 1.17 (3H, s, H_3_-18), 1.14–1.06 (4H, m, H_2_-34^a^, H_2_-35^a^), 0.94 (3H, d, *J* = 7.0 Hz, H_3_-17), 0.75 (3H, d, *J* = 7.0 Hz, H_3_-16); Exchangeable ^1^H signals observed: ^1^H NMR (DMSO-*d*_6_, 400 MHz) *δ* 9.22 (2H, br s, NH_2_-23), 8.50 (2H, br s, NH_2_-27), 7.48 (1H, t, *J* = 5.7 Hz, NH-38); ^13^C NMR (CD_3_OD, 100 MHz) *δ* 219.3 (C-3), 172.8 (C-39), 166.6 (C-21), 148.2 (C-42), 141.0 (C-19), 130.5 (C-44), 128.6 (C-43), 127.1 (C-45), 116.8 (C-20), 75.2 (C-11), 73.1 (C-14), 59.0 (C-4), 57.6 (C-41), 49.0 (C-22, C-28, C-40), 46.7 (C-9), 45.6 (C-13, C-24, C-26), 45.4 (C-12), 43.1 (C-5), 40.2 (C-37), 37.9 (C-10), 37.7 (C-6), 35.2 (C-2), 31.4 (C-8), 30.4 (C-30^b^), 30.3 (C-31^b^), 30.2 (C-32^b^), 30.1 (C-33^b^), 30.0 (C-34^b^), 28.2 (C-36), 28.0 (C-29), 27.8 (C-18), 27.5 (C-35^b^), 27.1 (C-7), 25.8 (C-1), 23.9 (C-25), 17.0 (C-16), 15.2 (C-15), 11.8 (C-17); (+)-HRESIMS *m*/*z* 874.6090 [M+H]^+^ (calcd for C_56_H_80_N_3_O_5_, 874.6092).^a,b^ Interchangeable assignments that could not be distinguished.

### 2.3. Antimicrobial Assays

The susceptibility of bacterial strains *Staphylococcus aureus* (ATCC 25923), *Escherichia coli* (ATCC 25922), and *Pseudomonas aeruginosa* (ATCC 27853 or PAO1) to antibiotics and compounds was determined using previously reported protocols [26]. Additional antimicrobial evaluations were determined against methicillin-resistant *S. aureus* (MRSA) (ATCC 43300) and *Cryptococcus neoformans* (ATCC 208821) at the Community for Open Antimicrobial Drug Discovery at The University of Queensland (Australia) using previously reported protocols (see Supplementary File) [26,27].

### 2.4. Cytotoxicity Assays

Cytotoxicity assays were conducted using previously reported protocols (see Supplementary File) [26,27].

### 2.5. Hemolytic Assay

Hemolysis assays were conducted using previously reported protocols (see Supplementary File) [26,27].

### 2.6. Real-Time Growth Cruves

Real-time growth curves were acquired using reported protocols (see Supplementary File) [18].

### 2.7. Measurement of ATP Efflux

Measurement of ATP efflux was undertaken using previously reported protocols (see Supplementary File) [26].

### 2.8. Nitrocefin Hydrolysis Assay

Nitrocefin hydrolysis assay was undertaken using previously reported protocols (see Supplementary File) [28].

## 3. Results and Discussion

### 3.1. Preliminary Set of Analogues

**Scheme 1:**
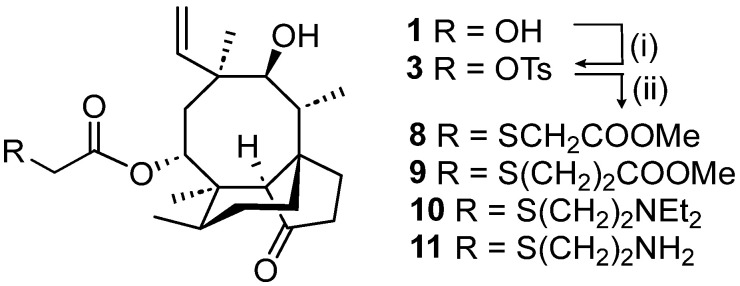
General method for preparation of thiol-substituted pleuromutilins **8**–**11**. Reagents and conditions: (i) *p*-Toluenesulfonylchloride, DMAP, CH_2_Cl_2_, 0 °C, N_2_, 4 h (57% yield); (ii) pre-incubate: KI, MeCN, 70 °C, N_2_, 30 min, then thiol **4**–**7**, DIPEA, 70 °C, N_2_, 2 h (34–60% yields).

**Figure 2:**
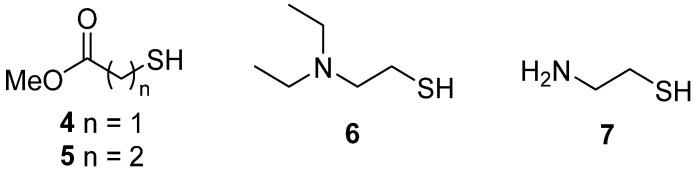
Structures of thiols **4**–**7**.

Prior to preparing a library of C-22 amine-substituted pleuromutilins, we first prepared a small set of thioether analogues that were used as positive controls in subsequent biological assays. Preparation of the derivatives began with the synthesis of pleuromutilin 22-*O*-tosylate (**3**) according to methods described in the literature [25] (Scheme 1). The tosylate was then converted in situ to the more reactive alkyliodide by initial preincubation with KI in anhydrous MeCN for 30 min. Subsequent addition of thiol **4**–**7** (Figure 2) in the presence of *N*,*N*-diisopropylethylamine (DIPEA) afforded the target analogues **8**–**11** in moderate yields of 34–60% (Scheme 1). In this set of analogues, tiamulin (**10**) was used as a positive control in subsequent biological studies, and two other analogues, **8** and **11**, had been previously reported [14,16].

**Figure 3:**
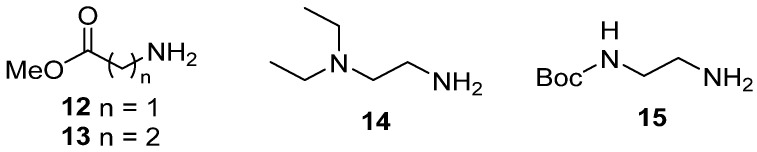
Structures of amines **12**–**15**.

Next, corresponding aza-analogues were prepared by reaction of tosylate **3** with amines **12**–**15** (Figure 3).

**Scheme 2:**
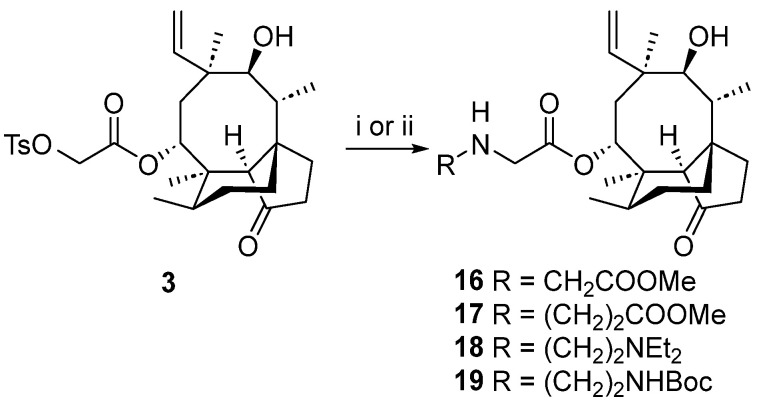
General method for preparation of amine-substituted pleuromutilins **16**–**19.** Reagents and conditions: (i) pre-incubate: KI, MeCN, 70 °C, N_2_, 30 min, then amine **12** or **13** (1.5 equiv.), DIPEA, 70 °C, N_2_, 4 h (16–59% yields); (ii) pre-incubate: KI, MeCN, 70 °C, N_2_, 30 min, then amine **14** or **15** (1.5 equiv.), DIPEA, 70 °C, N_2_, 6 h (19–20% yields).

Preparation of these derivatives proved more challenging, with longer reaction times required (4–6 h) to afford the target derivatives **16**–**19** in yields of 20–54% (Scheme 2). Of this set of four analogues, **18**, an aza-analogue of tiamulin, has been previously reported as a modestly active anti-*S. aureus* compound [16].

**Figure 4:**
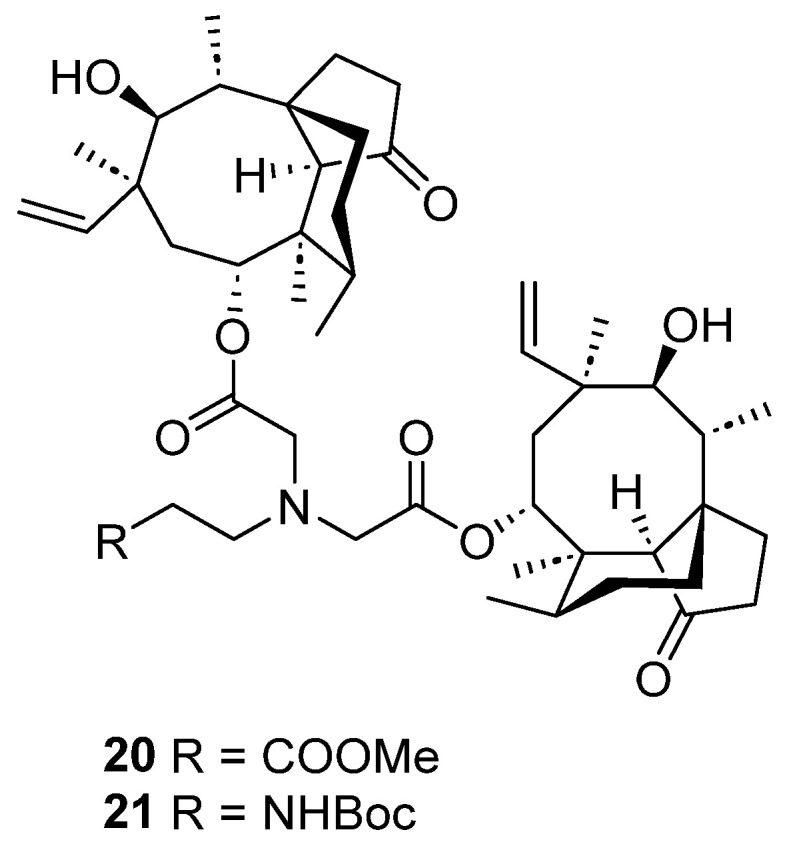
Structures of bis-pleuromutilins **20** and **21**.

The reactions of pleuromutilin tosylate **3** with amines **13** and **15** also afforded bis-pleuromutilins **20** and **21** (Figure 4) as low-yielding (13–16%) minor products. In addition to mass spectrometric evidence, the observation of a 2:1 relative ratio of ^1^H NMR signals for the pleuromutilin component and the pendant amine fragment were diagnostic in determining the structure of these two reaction products. Modification of the reaction conditions in an effort to increase the yield of these bis-pleuromutilin derivatives, by reducing the relative equivalents of amine **13** and **15** to tosylate **3** from 1.5 to 0.5 and by increasing the reaction time to 6 h, led to only minor increments in the yield of **20** and **21** to 19% and 20%, respectively (see Materials and Methods). Interestingly, bis-pleuromutilin analogues were not observed for the reactions of amines **12** or **14** with tosylate **3**.

**Figure 5:**
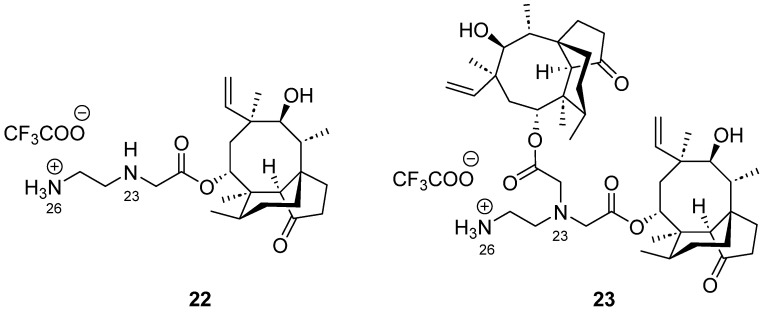
Structure amine-linked pleuromutilin **22** and bis-pleuromutilin **23**.

The Boc-protection groups present in analogues **19** and **21** were subsequently removed by reaction with trifluoroacetic acid (TFA) in CH_2_Cl_2_ to afford the primary amine-containing pleuromutilin analogues **22** and **23** as their mono-TFA salts (Figure 5) in yields of 84% and 91%, respectively. The protonation state of the products was determined by acquiring ^1^H NMR data in DMSO-*d*_6_ solvent, the spectra of which clearly identified N-26 in both compounds to be a positive-charged ammonium ion and N-23 to be a freebase secondary or tertiary amine.

The intrinsic antimicrobial activity of this preliminary set of analogues was evaluated against a range of Gram-positive (*S. aureus* and MRSA) and Gram-negative (*E. coli* and *P. aeruginosa*) bacteria using the thioether analogues **8**–**11** as reference compounds (Table 1). Cytotoxicity towards HEK293 (human kidney epithelial cell line, IC_50_) and hemolytic activity against human red blood cells (HC_10_) were also assessed (Table 1). As was anticipated, the thioether-linked analogues **8**–**11** exhibited potent activity against the Gram-positive bacteria MRSA (MIC ≤ 0.57 µM) and *S. aureus* (MIC 3.125 to 6.25 µM). In contrast the corresponding amine-linked analogues, **17**–**19** were significantly less active, exhibiting poor growth inhibition of MRSA (MIC 8.39 to >69 µM) and *S. aureus* (MIC 16 to 100 µM), in agreement with other studies [16]. The notable exception to this trend was methyl glycinate **16**, which demonstrated equivalent inhibition to the thioether analogues against MRSA (MIC ≤ 0.52 µM) and *S. aureus* (MIC 6.25 µM). Interestingly, the bis-pleuromutilins **20** and **23** exhibited stronger activity than their mono-substituted counterparts **17** and **19** against MRSA (MIC ≤ 0.30 µM) and *S. aureus* (MIC 3.125 to 12.5 µM). Unfortunately, none of the analogues, including those that contained primary amino groups (**11**, **22**, **23**), exhibited activity towards Gram-negative bacteria. Pleasing, however, was the observation that none of the analogues exhibited cytotoxicity or red blood cell hemolytic activity at the highest test concentration of 32 µg/mL. Based upon these preliminary results, we then targeted the synthesis of an expanded set of amine-substituted pleuromutilin analogues that contained longer C-22 extensions containing primary and secondary amines to explore the effect of chain length and number of positive charges on antibacterial activity.

### 3.2. Di-, Tri- and Tetraamine Analogues

**Figure 6:**
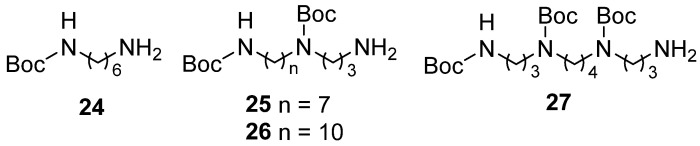
Structures of amines **24**–**27**.

**Scheme 3:**
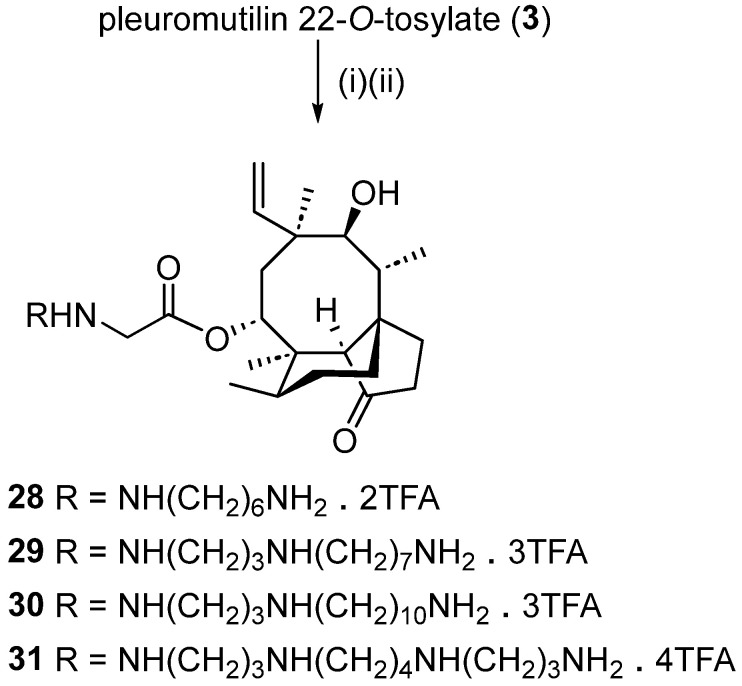
General method for preparation of amine-substituted pleuromutilins **28**–**31**. Reagents and conditions: (i) pre-incubate: KI, MeCN, 70 °C, N_2_, 30 min, then amine **24**–**27**, DIPEA, 70 °C, N_2_, 4 h (20–59%); (ii) TFA/CH_2_Cl_2_ (10% *v*/*v*), rt, 2 h (90–95%).

Longer-chain di-, tri-, and tetraamine-substituted pleuromutilin analogues were prepared using the now standard protocol. Thus, the reaction of Boc-protected amines **24**–**27** [23] (Figure 6) with tosylate **3** afforded Boc-protected intermediates in modest yields of 47–59%, which were subsequently deprotected using TFA in CH_2_Cl_2_ to afford amino pleuromutilin derivatives **28**–**31** (Scheme 3).

The biological evaluation of these four analogues (Table 2) identified the retention of anti-*S. aureus* activities, and in the case of the longer-chain variants **30** and **31**, the growth inhibition of *E. coli* ATCC 25922 was observed (MIC 6.7–12.3 µM). The results for these two analogues were particularly pleasing, validating the premise that incorporating primary and/or secondary amines into a C-22-functionalized pleuromutilin can indeed lead to increased potency towards *E. coli*. Analogues **28**–**30** also exhibited strong activity towards *Cryptococcus neoformans* and were non-cytotoxic towards HEK293 cells and non-hemolytic against human red blood cells.

**Figure 7:**
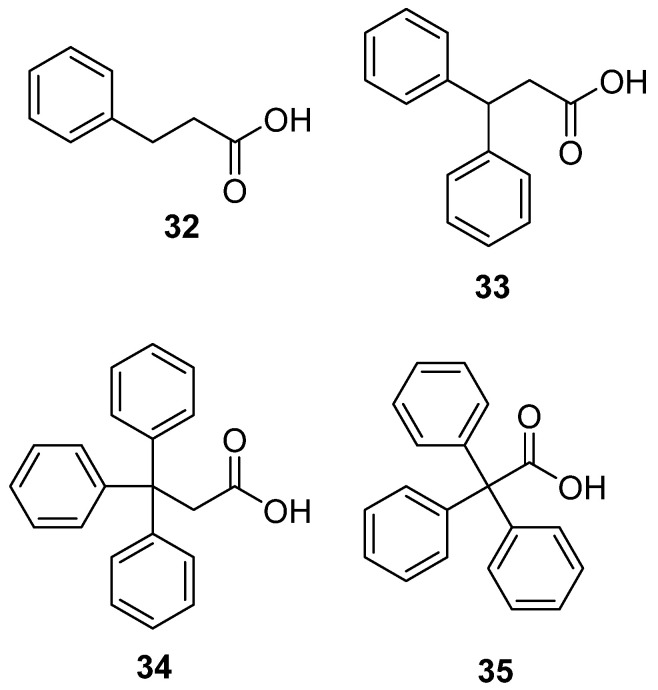
Structures of aromatic head groups **32**–**35**.

We have previously reported that phenyl/diphenyl/triphenylamido-substituted polyamines show a pronounced ability to penetrate bacterial membranes [26]. To explore whether these membrane penetration properties, and the resultant biological activities, could be enhanced by the addition of a lipophilic group to the end of the C-22 extension, a further set of amide-linked pleuromutilin derivatives were prepared using the aromatic head groups 3-phenylpropanoic acid (**32**), 3,3-diphenylpropanoic acid (**33**), 3,3,3-triphenylpropanoic acid (**34**), and 3,3,3-triphenylacetic acid (**35**) (Figure 7).

**Scheme 4:**
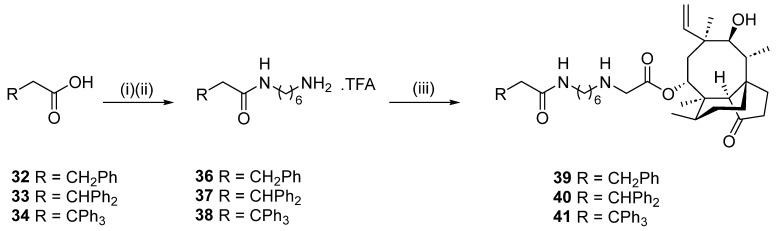
General method for synthesis of target pleuromutilin derivatives **39**–**41**. Reagents and conditions: (i) preincubate: carboxylic acid RCO_2_H (**32**–**34**), EDC·HCl, DMAP, DMF/CH_2_Cl_2_ (1:1), 0 °C, N_2_, 30 min, then add amine **24**, rt, N_2_, 18 h (65–80%); (ii) TFA/CH_2_Cl_2_ (10% *v*/*v*), rt, 2 h (89–99%); (iii) pre-incubate: tosylate **3**, KI, MeCN, 70 °C, N_2_, 30 min, then add amines **36**, **37** or **38**, DIPEA, 70 °C, N_2_, 4 h (40–56%).

This set of analogues was prepared by two routes: (1) reaction of pleuromutilin tosylate **3** with an amine that is already functionalized with lipophilic head groups or (2) attachment of lipophilic head groups to an amine-functionalized pleuromutilin intermediate. As an example of the first route, carboxylic acids **32**–**34** were coupled to *tert*-butyl (6-aminohexyl)carbamate (**24**) with *N*-(3-dimethylaminopropyl)-*N*′-ethylcarbodiimide hydrochloride (EDC·HCl) and 4-dimethylaminopyridine (DMAP) in anhydrous DMF/CH_2_Cl_2_ followed by deprotection with TFA in CH_2_Cl_2_ to afford amino-amides **36**–**38** as their mono-TFA salts (Scheme 4). These amino-amides were then reacted with tosylate **3** according to the established protocol to afford the target compounds **39**–**41** in moderate yields of 40–56%.

**Scheme 5:**
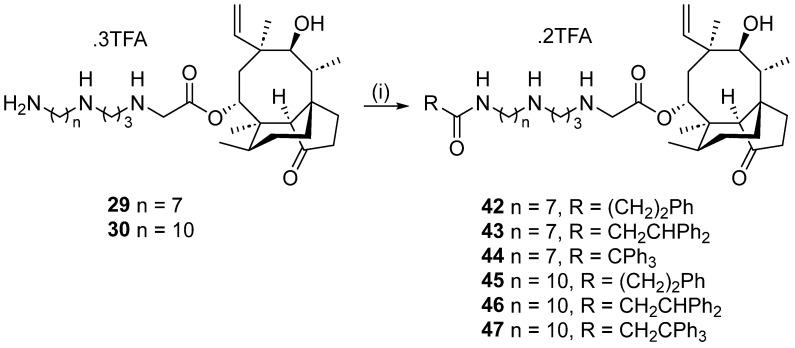
General method for preparation of target pleuromutilin derivatives **42**–**47**. Reagents and conditions: (i) carboxylic acid **32**–**35**, HBTU, HOBt, DIPEA, DMF, rt, N_2_, 1.5 h (46–75% yields).

A further set of pleuromutilin–arylcarboxamide derivatives were prepared using an alternative, late-stage functionalization method. Thus, coupling of carboxylic acids **32**–**35** to amino-functionalized pleuromutilins **29** and **30** utilizing coupling reagents hydroxybenzotriazole (HOBt), 2-(1*H*-benzotriazole-1-yl)-1,1,3,3-tetramethyluronium hexafluorophosphate (HBTU) and DIPEA in anhydrous DMF afforded, after purification via reversed-phase column chromatography, the target compounds **42**–**47** as their di-TFA salts in 46–75% yields (Scheme 5).

Biological evaluation of lipophilic analogues **39**–**47** identified a number of trends, including the following: (i) compounds typically retained Gram-positive antibacterial activity, with notable exceptions being **41** and **42**; (ii) complete loss of activity towards *E. coli*; and (iii) members of this set were either cytotoxic and/or hemolytic (Table 2).

**Figure 8:**
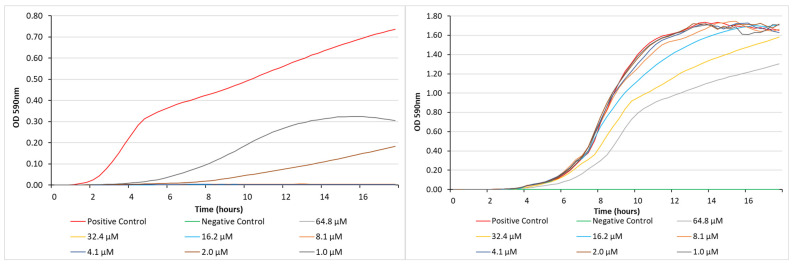
Bacterial growth inhibition exhibited by tiamulin **10** against *S. aureus* ATCC 25923 (**left**) and *P. aeruginosa* PAO1 (**right**) at different concentrations. Positive control was bacteria only and negative control was media only.

**Figure 9:**
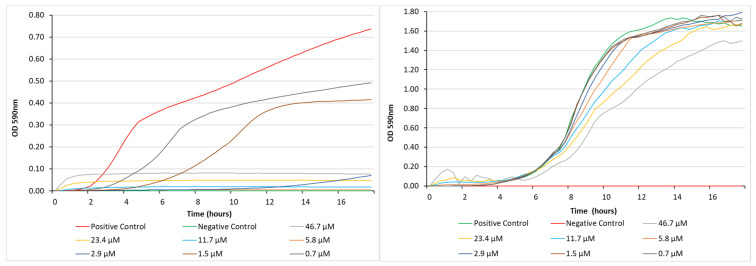
Bacterial growth inhibition exhibited by **40** against *S. aureus* ATCC 25923 (**left**) and *P. aeruginosa* PAO1 (**right**) at different concentrations. Positive control was bacteria only and negative control was media only.

Real-time growth inhibition curves of tiamulin **10** and analogue **40** against *S. aureus* ATCC 25923 and *P. aeruginosa* PAO1 were used to assess the kinetics of their antibacterial activity. Tiamulin **10** completely inhibited *S. aureus* growth at 4.1 µM with bacterial growth detected after 4 h at the lowest test concentration (1.0 µM) (Figure 8). The growth inhibition kinetics of **40** were similar, with complete inhibition observed at 5.8 µM and growth observed after 2 h at 0.7 µM (Figure 9). Both compounds failed to inhibit the growth of *P. aeruginosa* at the highest tested concentration (Figure 8 and Figure 9).

We have previously reported that 3,3-diphenylpropamido-functionalized amines can exhibit antimicrobial activities due to their ability to perturb bacterial outer membranes [26]. The abilities of analogues **28**, **29**, **40** and **41**, the latter as a pleuromutilin-containing negative control, to disrupt the outer membrane of *S. aureus* 25,923 were evaluated using an ATP release biochemical assay. Compounds were tested at a single fixed dose of 100 µg/mL, which is well above the MIC of each of **28**, **29** and **40** against the organism. In all cases, no Gram-positive bacterial membrane disruption was detected (Figure S27). A similar evaluation of the abilities of tiamulin (**10**) and **40** to perturb the outer membrane of *P. aeruginosa* PAO1, evaluated using a nitrocefin colorimetric assay [26,28], also failed to indicate any membrane disruption (Figure S28). We concluded that the diphenylpropanamide-linked pleuromutilin analogue **40**, despite exhibiting a strong growth inhibition of Gram-positive bacteria, had little to no effect on bacterial membrane stability.

Substituted polyamines are also known to enhance the action of antibiotics towards drug resistant bacteria [29]. We evaluated analogues **30** and **31** for their ability to enhance the antibiotic activity of doxycycline against *P. aeruginosa* ATCC 27853. Using a low 2 µg/mL, ineffectual, dose of doxycycline, **30** was found to be a very weak enhancer, leading to a 2-fold increase in effectiveness against the Gram-negative organism. In contrast, the spermine analogue **31** was a moderate enhancer, with an 8-fold increase in effectiveness, when used in combination with the low dose of doxycycline.

## 4. Conclusions

It has been suggested that the presence of ionizable nitrogens in small-molecule Gram-positive only antibiotics can lead to broad-spectrum activity. The present study applied these principles to pleuromutilins by preparing and evaluating a series of C-22 substituted amine- and polyamine-linked analogues. While simple amino esters or diamines **16**–**18**, and **22** were as potent against *S. aureus* and MRSA as their thiol variants, they lacked activity against Gram-negative bacteria. Derivatives with longer triamine and polyamine side chains (**30**, **31**) did exhibit activity against both *S. aureus* and *E. coli*, suggesting that the primary amine either needs to be further from the pleuromutilin core or that several amines are required for optimal Gram-negative activity. Intriguingly, spermine derivative **31** also demonstrated potentiation properties and enhanced the action of doxycycline towards *P. aeruginosa* by 8-fold, presenting an interesting scaffold for future pleuromutilin analogues with broader spectrums of activity.

## Figures and Tables

**Table 1:** Antimicrobial, cytotoxic, and hemolytic activities of analogues **1**, **3**, **8**–**11**, **16**–**18**, **20**, **22**, and **23**.

Compounds	MIC (µM)				Cyto (µM) ^e^	HC_10_ (µM) ^f^
	*S. a* ^a^	MRSA ^b^	*P. a* ^c^	*E. c* ^d^		
**1**	3.125	n.t. ^g^	800	400	n.t. ^g^	n.t. ^g^
**3**	3.125	≤0.47	800	400	>60	>60
**8**	3.125	≤0.54	800	400	>69	>69
**9**	3.125	≤0.52	800	400	>67	>67
**10**	3.125	≤0.51	800	200	>65	>65
**11**	6.25	≤0.57	800	400	>73	>73
**16**	6.25	≤0.56	800	400	>71	>71
**17**	100	>69	800	400	>69	>69
**18**	50	8.39	800	400	>67	>67
**20**	12.5	≤0.30	800	400	>39	>39
**22**	16	9.5	374	>374	>76	>76
**23**	3.125	≤0.25	800	400	>32	>32
**Streptomycin**	21.5		21.5	21.5		
**Chloramphenicol**	1.5–3					
**Vancomycin**		0.7				
**Colistin**			1	2		
**Tamoxifen**					24	
**Melittin**						0.95

MIC data presented as an average of two or three independent experiments; ^a^ *Staphylococcus aureus* ATCC 25923; ^b^ MRSA ATCC 43300; ^c^ *Pseudomonas aeruginosa* ATCC 27853; ^d^ *Escherichia coli* ATCC 25922; ^e^ Concentration of compound at 50% cytotoxicity on HEK293 human embryonic kidney cells; ^f^ Concentration of compound at 10% hemolytic activity on human red blood cells; ^g^ Not tested.

**Table 2:** Antimicrobial, cytotoxic, and hemolytic activities of analogues **28**–**31**, **39**–**47.**

Compounds	MIC (µM)					Cyto (µM) ^f^	HC_10_ (µM) ^g^
	*S. a* ^a^	MRSA ^b^	*P. a* ^c^	*E. c* ^d^	*C. n* ^e^		
**28**	4.0	22.7	800	100	≤0.35	>45	>45
**29**	32	4.5	225	>225	≤0.28	>36	>36
**30**	13.4	6.7	107	6.7	≤0.27	>34	>34
**31**	6.1	n.t. ^h^	196	12.3	n.t. ^h^	n.t. ^h^	n.t. ^h^
**39**	4	n.t. ^h^	>187	>187	>53	>53	>53
**40**	<4	11.7	>187	>187	≤0.36	>47	>47
**41**	>64	>42	>263	>263	10.5	>42	>42
**42**	27.5	13.8	>220	220	>35	>35	>35
**43**	25	6.3	>203	102	2.03	11.2	21.7
**44**	3.0	3.0	>191	95.6	1.91	11.3	2.22
**45**	6.6	3.3	>211	53	≤0.26	>34	>34
**46**	6.1	6.1	>195	>195	1.95	8.6	0.89
**47**	2.8	2.8	>181	181	≤0.26	>29	1.5
**Streptomycin**	21.5		21.5	21.5			
**Chloramphenicol**	1.5–3						
**Vancomycin**		0.7					
**Colistin**			1	2			
**Fluconazole**					26		
**Tamoxifen**						24	
**Melittin**							0.95

MIC data presented as an average of two or three independent experiments; ^a^ *Staphylococcus aureus* ATCC 25923; ^b^ MRSA ATCC 43300; ^c^ *Pseudomonas aeruginosa* ATCC 27853; ^d^ *Escherichia coli* ATCC 25922; ^e^ *Cryptococcus neoformans* (ATCC 208821); ^f^ Concentration of compound at 50% cytotoxicity on HEK293 human embryonic kidney cells; ^g^ Concentration of compound at 10% hemolytic activity on human red blood cells; ^h^ Not tested.

## Data Availability

Data are contained within the article or Supplementary Materials.

## Supplementary Materials

The following supporting information can be downloaded at https://www.mdpi.com/article/10.3390/antibiotics13111018/s1: Biological assay protocols: Figure S1. ^1^H (CDCl_3_, 400 MHz) and ^13^C (CDCl_3_, 100 MHz) NMR spectra for **8**; Figure S2. ^1^H (CDCl_3_, 400 MHz) and ^13^C (CDCl_3_, 100 MHz) NMR spectra for **9**; Figure S3. ^1^H (CDCl_3_, 400 MHz) and ^13^C (CDCl_3_, 100 MHz) NMR spectra for **11**; Figure S4. ^1^H (CDCl_3_, 400 MHz) and ^13^C (CDCl_3_, 100 MHz) NMR spectra for **16**; Figure S5. ^1^H (CDCl_3_, 400 MHz) and ^13^C (CDCl_3_, 100 MHz) NMR spectra for **17**; Figure S6. ^1^H (CDCl_3_, 400 MHz) and ^13^C (CDCl_3_, 100 MHz) NMR spectra for **18**; Figure S7. ^1^H (CDCl_3_, 400 MHz) and ^13^C (CDCl_3_, 100 MHz) NMR spectra for **19**; Figure S8. ^1^H (CDCl_3_, 400 MHz) and ^13^C (CDCl_3_, 100 MHz) NMR spectra for **20**; Figure S9. ^1^H (CD_3_OD, 400 MHz) and ^13^C (CD_3_OD, 100 MHz) NMR spectra for **22**; Figure S10. ^1^H (CD_3_OD, 400 MHz) and ^13^C (CD_3_OD, 100 MHz) NMR spectra for **23**; Figure S11. ^1^H (CD_3_OD, 400 MHz) and ^13^C (CD_3_OD, 100 MHz) NMR spectra for **28**; Figure S12. ^1^H (CD_3_OD, 400 MHz) and ^13^C (CD_3_OD, 100 MHz) NMR spectra for **29**; Figure S13. ^1^H (CD_3_OD, 400 MHz) and ^13^C (CD_3_OD, 100 MHz) NMR spectra for **30**; Figure S14. ^1^H (CD_3_OD, 400 MHz) and ^13^C (CD_3_OD, 100 MHz) NMR spectra for **31**; Figure S15. ^1^H (CD_3_OD, 400 MHz) and ^13^C (CD_3_OD, 100 MHz) NMR spectra for **36**; Figure S16. ^1^H (CD_3_OD, 400 MHz) and ^13^C (CD_3_OD, 100 MHz) NMR spectra for **37**; Figure S17. ^1^H (CD_3_OD, 400 MHz) and ^13^C (CD_3_OD, 100 MHz) NMR spectra for **38**; Figure S18. ^1^H (CDCl_3_, 400 MHz) and ^13^C (CDCl_3_, 100 MHz) NMR spectra for **39**; Figure S19. ^1^H (CDCl_3_, 400 MHz) and ^13^C (CDCl_3_, 100 MHz) NMR spectra for **40**; Figure S20. ^1^H (CDCl_3_, 400 MHz) and ^13^C (CDCl_3_, 100 MHz) NMR spectra for **41**; Figure S21. ^1^H (CD_3_OD, 400 MHz) and ^13^C (CD_3_OD, 100 MHz) NMR spectra for **42**; Figure S22. ^1^H (CD_3_OD, 400 MHz) and ^13^C (CD_3_OD, 100 MHz) NMR spectra for **43**; Figure S23. ^1^H (CD_3_OD, 400 MHz) and ^13^C (CD_3_OD, 100 MHz) NMR spectra for **44**; Figure S24. ^1^H (CD_3_OD, 400 MHz) and ^13^C (CD_3_OD, 100 MHz) NMR spectra for **45**; Figure S25. ^1^H (CD_3_OD, 400 MHz) and ^13^C (CD_3_OD, 100 MHz) NMR spectra for **46**; Figure S26. ^1^H (CD_3_OD, 400 MHz) and ^13^C (CD_3_OD, 100 MHz) NMR spectra for **47**; Figure S27. ATP release in *S. aureus* ATCC 25923 exhibited by selected compounds (**28**, **29**, **40**, and **41**) as determined using an ATP efflux assay. Squalamine (100 μg/mL) was used as the positive control and water as the negative control. Compounds were tested at a fixed concentration of 100 μg/mL and results reported as percentage (%) relative to positive control; Figure S28. The ability of **10** (left) and **40** (right) to act as membrane disruptors in *P. aeruginosa* PAO1 as determined using a nitrocefin hydrolysis assay. Polymixin B (PMB) was the positive control (128 μg/mL) and the negative control was bacteria with nitrocefin.
