# Supplementary material for: Synthesis and Antibacterial Activity of Alkylamine-Linked Pleuromutilin Derivatives
Source: Antibiotics (Basel). 2024 Oct 29;13(11):1018. doi: 10.3390/antibiotics13111018 (PMC11591448; doi:10.3390/antibiotics13111018)
Supplement: Supplementary file 1 [file antibiotics-13-01018-s001.zip › antibiotics-3271690-supplementary.pdf]

Supporting Information

Synthesis and Antibacterial Activity of Alkylamine-linked Pleuromutilin Derivatives

Kerrin Hainsworth [1], Melissa M. Cadelis [1,2], Florent Rouvier [3], Jean Michel Brunel[3] and Brent R. Copp [1,*]

1 School of Chemical Sciences, The University of Auckland, Private Bag 92019, Auckland 1142, New Zealand
2 School of Medical Sciences, The University of Auckland, Private Bag 92019, Auckland 1142, New Zealand
3 Membranes et Cibles Thérapeutiques, INSERM, Aix-Marseille Universite, 27 bd Jean Moulin, 13385 Marseille, France
***** Correspondence: b.copp@auckland.ac.nz

Contents

**Biological assay protocols**	**S3**
**Figure S1** ^{1}H ($CDCl_3$, 400 MHz) and ^{13}C ($CDCl_3$, 100 MHz) NMR spectra for **8**.	**S5**
**Figure S2** ^{1}H ($CDCl_3$, 400 MHz) and ^{13}C ($CDCl_3$, 100 MHz) NMR spectra for **9**.	**S6**
**Figure S3** ^{1}H ($CDCl_3$, 400 MHz) and ^{13}C ($CDCl_3$, 100 MHz) NMR spectra for **11**.	**S7**
**Figure S4** ^{1}H ($CDCl_3$, 400 MHz) and ^{13}C ($CDCl_3$, 100 MHz) NMR spectra for **16**.	**S8**
**Figure S5** ^{1}H ($CDCl_3$, 400 MHz) and ^{13}C ($CDCl_3$, 100 MHz) NMR spectra for **17**.	**S9**
**Figure S6** ^{1}H ($CDCl_3$, 400 MHz) and ^{13}C ($CDCl_3$, 100 MHz) NMR spectra for **18**.	**S10**
**Figure S7** ^{1}H ($CDCl_3$, 400 MHz) and ^{13}C ($CDCl_3$, 100 MHz) NMR spectra for **19**.	**S11**
**Figure S8** ^{1}H ($CDCl_3$, 400 MHz) and ^{13}C ($CDCl_3$, 100 MHz) NMR spectra for **20**.	**S12**
**Figure S9** ^{1}H (CD_3OD, 400 MHz) and ^{13}C (CD_3OD, 100 MHz) NMR spectra for **22**.	**S13**
**Figure S10** ^{1}H (CD_3OD, 400 MHz) and ^{13}C (CD_3OD, 100 MHz) NMR spectra for **23**.	**S14**
**Figure S11** ^{1}H (CD_3OD, 400 MHz) and ^{13}C (CD_3OD, 100 MHz) NMR spectra for **28**.	**S15**
**Figure S12** ^{1}H (CD_3OD, 400 MHz) and ^{13}C (CD_3OD, 100 MHz) NMR spectra for **29**.	**S16**
**Figure S13** ^{1}H (CD_3OD, 400 MHz) and ^{13}C (CD_3OD, 100 MHz) NMR spectra for **30**.	**S17**
**Figure S14** ^{1}H (CD_3OD, 400 MHz) and ^{13}C (CD_3OD, 100 MHz) NMR spectra for **31**.	**S18**
**Figure S15** ^{1}H (CD_3OD, 400 MHz) and ^{13}C (CD_3OD, 100 MHz) NMR spectra for **36**.	**S19**
**Figure S16** ^{1}H (CD_3OD, 400 MHz) and ^{13}C (CD_3OD, 100 MHz) NMR spectra for **37**.	**S20**
**Figure S17** ^{1}H (CD_3OD, 400 MHz) and ^{13}C (CD_3OD, 100 MHz) NMR spectra for **38**.	**S21**
**Figure S18** ^{1}H ($CDCl_3$, 400 MHz) and ^{13}C ($CDCl_3$, 100 MHz) NMR spectra for **39**.	**S22**
**Figure S19** ^{1}H ($CDCl_3$, 400 MHz) and ^{13}C ($CDCl_3$, 100 MHz) NMR spectra for **40**.	**S23**
**Figure S20** ^{1}H ($CDCl_3$, 400 MHz) and ^{13}C ($CDCl_3$, 100 MHz) NMR spectra for **41**.	**S24**
**Figure S21** ^{1}H (CD_3OD, 400 MHz) and ^{13}C (CD_3OD, 100 MHz) NMR spectra for **42**.	**S25**
**Figure S22** ^{1}H (CD_3OD, 400 MHz) and ^{13}C (CD_3OD, 100 MHz) NMR spectra for **43**.	**S26**
**Figure S23** ^{1}H (CD_3OD, 400 MHz) and ^{13}C (CD_3OD, 100 MHz) NMR spectra for **44**.	**S27**

Figure S24 ^{1}H (CD_3OD, 400 MHz) and ^{13}C (CD_3OD, 100 MHz) NMR spectra for **45**. **S28**

Figure S25 ^{1}H (CD_3OD, 400 MHz) and ^{13}C (CD_3OD, 100 MHz) NMR spectra for **46**. **S29**

Figure S26 ^{1}H (CD_3OD, 400 MHz) and ^{13}C (CD_3OD, 100 MHz) NMR spectra for **47**. **S30**

Figure S27 ATP release in *S. aureus* ATCC 25923 exhibited by selected compounds (**28**, **29**, **40** and **41**) as determined using an ATP efflux assay. Squalamine (100 µg/mL) was used as the positive control and water as the negative control. Compounds were tested at a fixed concentration of 100 µg/mL and results reported as percentage (%) relative to positive control. **S31**

Figure S28. The ability of **10** (left) and **40** (right) to act as membrane disruptors in *P. aeruginosa* PAO1 as determined using a nitrocefin hydrolysis assay. Polymixin B (PMB) was the positive control (128 µg/mL) and the negative control was bacteria with nitrocefin. **S31**

Biological assay protocols.

Antimicrobial assays

Antimicrobial evaluation against *Staphylococcus aureus* (MRSA) (ATCC 43300), *Pseudomonas aeruginosa* (ATCC 27853), *Escherichia coli* (ATCC 25922), *Klebsiella pneumoniae* (ATCC 700603), *Acinetobacter baumannii* (ATCC 19606), *Candida albicans* (ATCC 90028), and *Cryptococcus neoformans* (ATCC 208821) was undertaken at the Community for Open Antimicrobial Drug Discovery at The University of Queensland (Australia) according to their standard protocols. For antimicrobial assays, the tested strains were cultured in either Luria broth (LB) (In Vitro Technologies, USB75852), nutrient broth (NB) (Becton Dickson, 234000), or MHB at 37 °C overnight. A sample of culture was then diluted 40-fold in fresh MHB and incubated at 37 °C for 1.5−2 h. The compounds were serially diluted 2-fold across the wells of 96-well plates (Corning 3641, nonbinding surface), with compound concentrations ranging from 0.015 to 64 μg/ mL, plated in duplicate. The resultant mid log phase cultures were diluted to the final concentration of 1×10^6 CFU/mL; then, 50 μL was added to each well of the compound containing plates giving a final compound concentration range of 0.008 to 32 μg/mL and a cell density of 5×10^5 CFU/mL. All plates were then covered and incubated at 37 °C for 18 h. Resazurin was added at 0.001% final concentration to each well and incubated for 2 h before MICs were read by eye.

For the antifungal assay, fungi strains were cultured for 3 days on YPD agar at 30 °C. A yeast suspension of 1×10^6 to 5×10^6 CFU/mL was prepared from five colonies. These stock suspensions were diluted with yeast nitrogen base (YNB) (Becton Dickinson, 233520) broth to a final concentration of 2.5×10^3 CFU/mL. The compounds were serially diluted 2-fold across the wells of 96-well plates (Corning 3641, nonbinding surface), with compound concentrations ranging from 0.015 to 64 μg/mL and final volumes of 50 μL, plated in duplicate. Then, 50 μL of the fungi suspension that was previously prepared in YNB broth to the final concentration of 2.5×10^3 CFU/mL was added to each well of the compound-containing plates, giving a final compound concentration range of 0.008 to 32 μg/mL. Plates were covered and incubated at 35 °C for 36 h without shaking. *C. albicans* MICs were determined by measuring the absorbance at OD_{530}. For *C. neoformans*, resazurin was added at 0.006% final concentration to each well and incubated for a further 3 h before MICs were determined by measuring the absorbance at $OD_{570-600}$.

Colistin and vancomycin were used as positive bacterial inhibitor standards for Gram-negative and Gram-positive bacteria, respectively. Fluconazole was used as a positive fungal inhibitor standard for *C. albicans* and *C. neoformans*. The antibiotics were provided in 4 concentrations, with 2 above and 2 below its MIC value, and plated into the first 8 wells of column 23 of the 384-well NBS plates. The quality control (QC) of the assays was determined by the antimicrobial controls and the Z'-factor (using positive and negative controls). Each plate was deemed to fulfil the quality criteria (pass QC), if the Z'-factor was above 0.4, and the antimicrobial standards showed full range of activity, with full growth inhibition at their highest concentration, and no growth inhibition at their lowest concentration.

Determination of the MICs of antibiotics in the presence of synergizing compounds

Briefly, restoring enhancer concentrations were determined with an inoculum of 5×10^5 CFU in 200 µL of MH broth containing two-fold serial dilutions of each derivative in the presence of doxycycline at 2 µg/mL. The lowest concentration of the polyamine adjuvant that completely inhibited visible growth after incubation for 18 h at 37 °C was determined. These measurements were independently repeated in triplicate.

Cytotoxicity assays

HEK293 cells were counted manually in a Neubauer hemocytometer and plated at a density of 5,000 cells/well into each well of the 384-well plates containing the 25x (2 µL) concentrated compounds. The medium used was Dulbecco's modified eagle medium (DMEM) supplemented with 10% fetal bovine serum (FBS). Cells were incubated together with the compounds for 20 h at 37 °C, 5% CO_2. To measure cytotoxicity, 5 µL (equals 100 µM final) of resazurin was added to each well after incubation, and incubated for further 3 h at 37 °C with 5% CO_2. After final incubation fluorescence intensity was measured as Fex 560/10 nm, em 590/10 nm ($F_{560/590}$) using a Tecan M1000 Pro monochromator plate reader. CC_{50} values (concentration at 50% cytotoxicity) were calculated by normalizing the fluorescence readout, with 74 µg/mL tamoxifen as negative control (0%) and normal cell growth as positive control (100%). The concentration-dependent percentage cytotoxicity was fitted to a dose response function (using Pipeline Pilot) and CC_{50} values determined.

Hemolytic assays

Human whole blood was washed three times with 3 volumes of 0.9% NaCl and then resuspended in same to a concentration of 0.5 x 10^8 cells/mL, as determined by manual cell count in a Neubauer hemocytometer. The washed cells were then added to the 384-well compound-containing plates for a final volume of 50 µL. After a 10 min shake on a plate shaker the plates were then incubated for 1 h at 37 °C. After incubation, the plates were centrifuged at 1,000g for 10 min to pellet cells and debris, 25 µL of the supernatant was then transferred to a polystyrene 384-well assay plate. Hemolysis was determined by measuring the supernatant absorbance at 405 mm (OD_{405}). The absorbance was measured using a Tecan M1000 Pro monochromator plate reader. HC_{10} and HC_{50} (concentration at 10% and 50% hemolysis, respectively) were calculated by curve fitting the inhibition values *vs*. log (concentration) using a sigmoidal dose-response function with variable fitting values for top, bottom and slope.

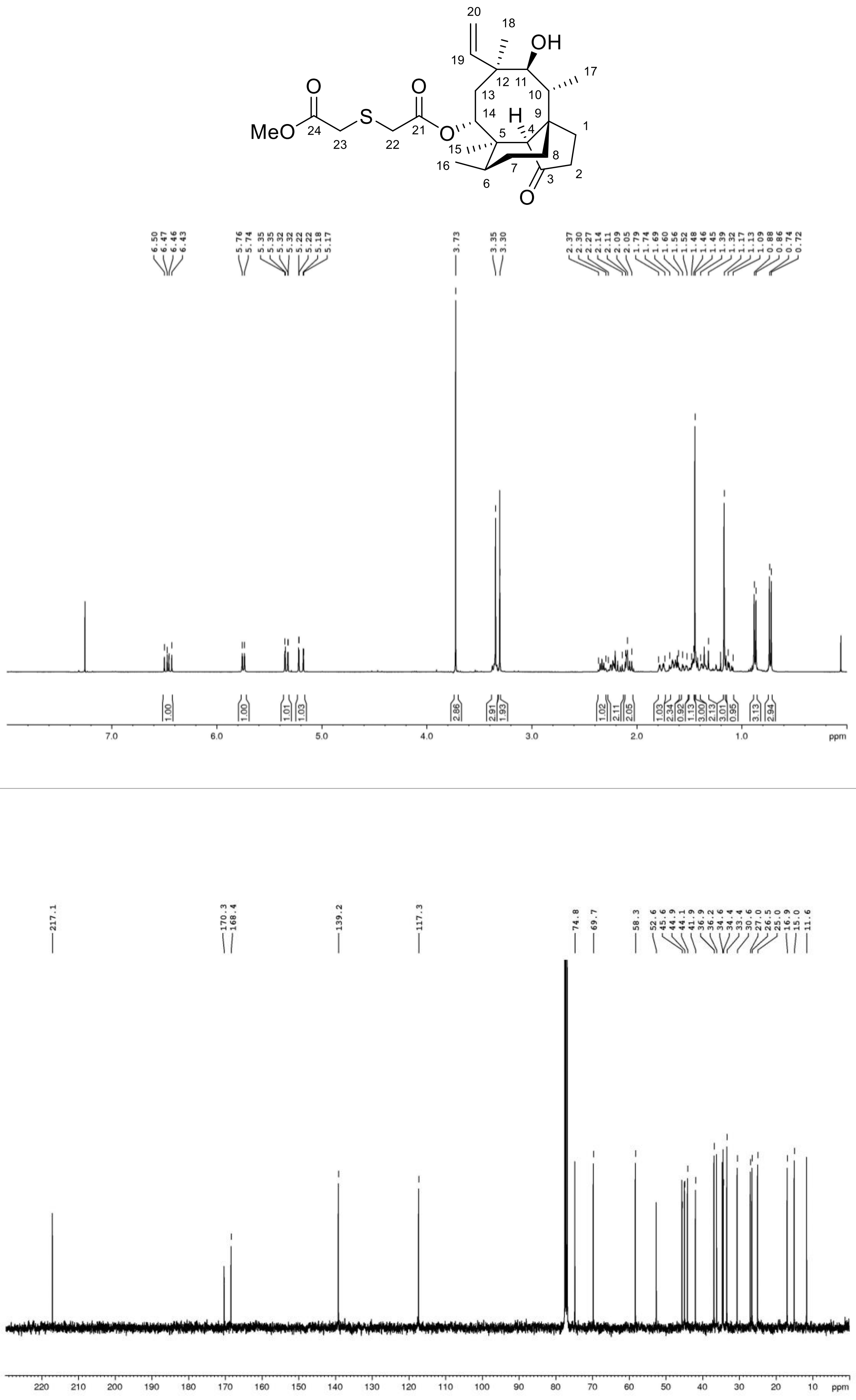

Figure S1 ^{1}H ($CDCl_3$, 400 MHz) and ^{13}C ($CDCl_3$, 100 MHz) NMR spectra for **8**.

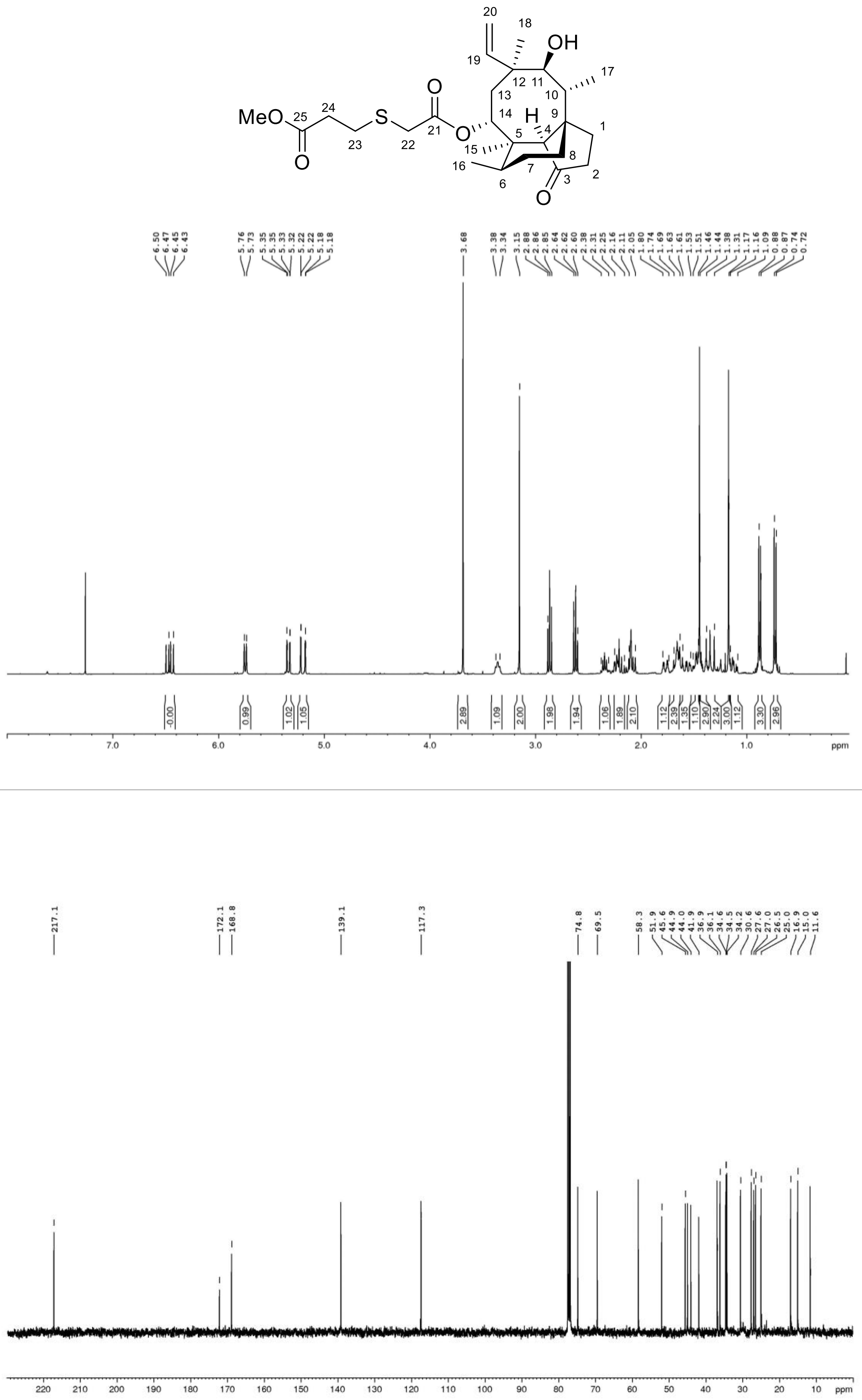

Figure S2 ^{1}H ($CDCl_3$, 400 MHz) and ^{13}C ($CDCl_3$, 100 MHz) NMR spectra for **9**.

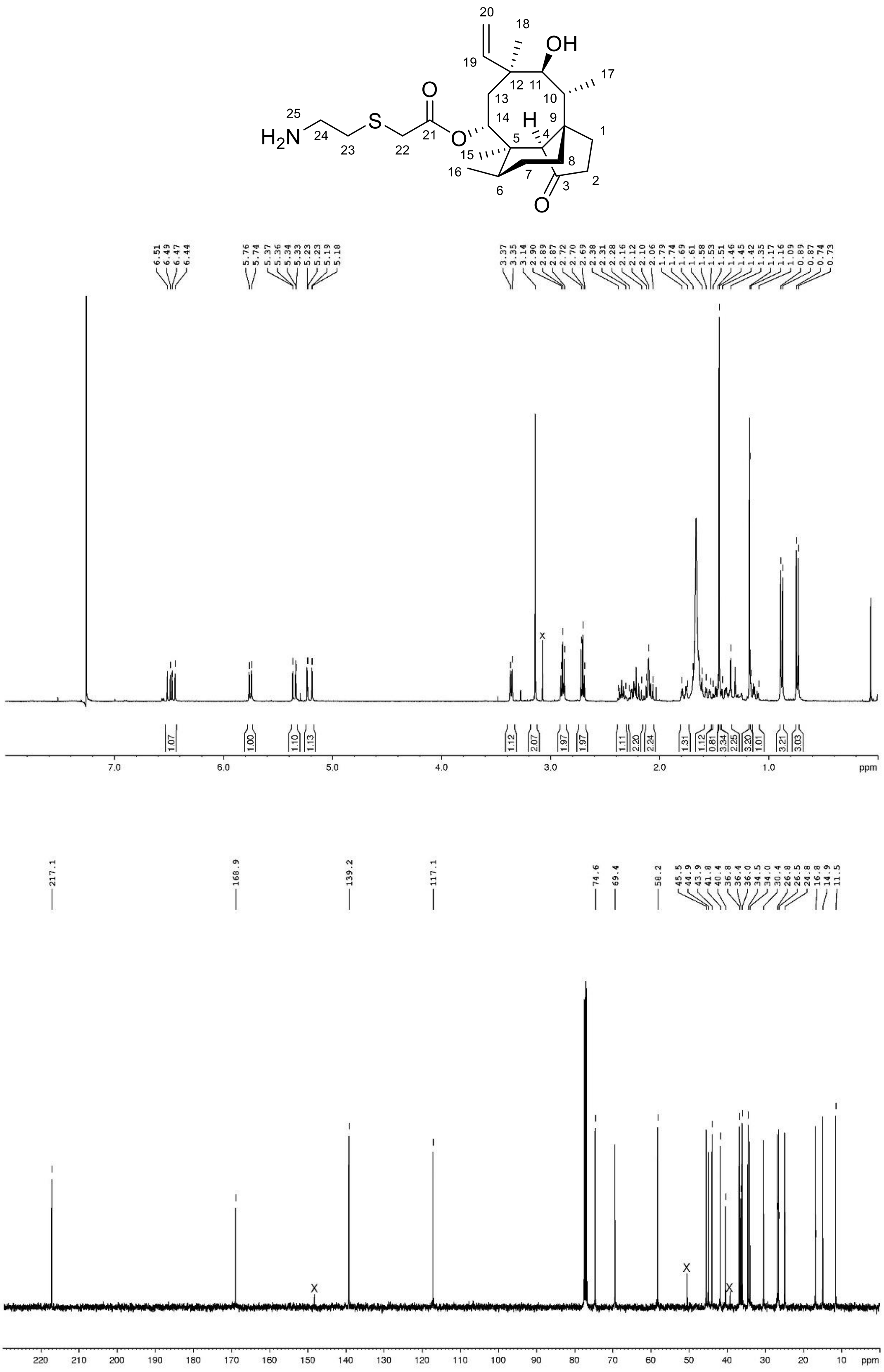

Figure S3 ^{1}H ($CDCl_3$, 400 MHz) and ^{13}C ($CDCl_3$, 100 MHz) NMR spectra for **11**.

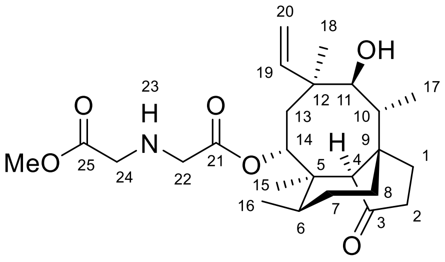

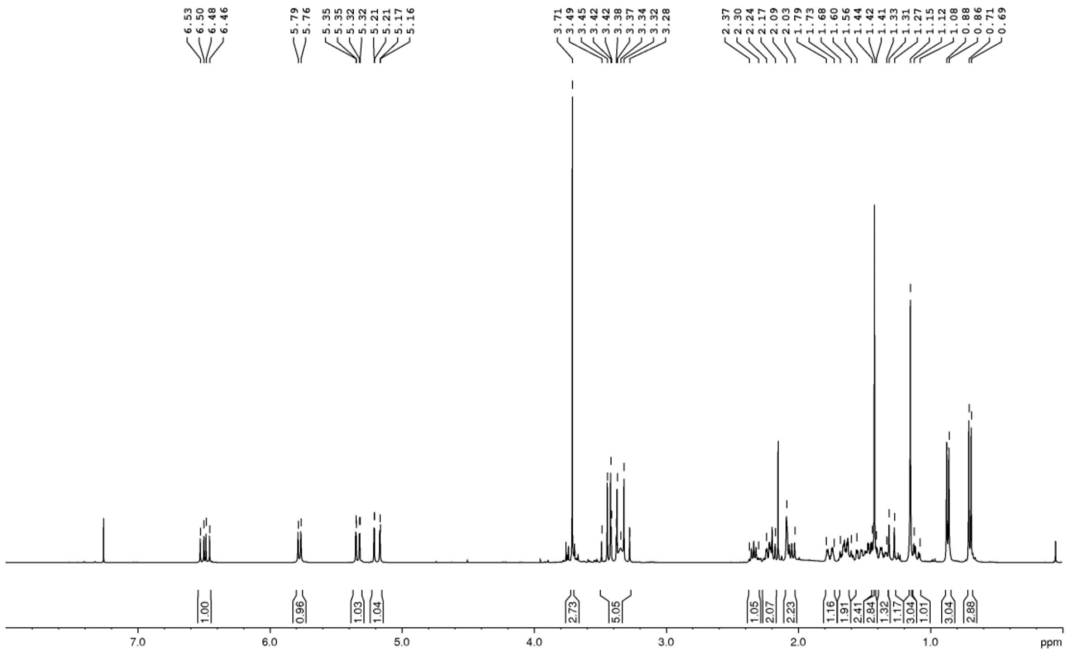

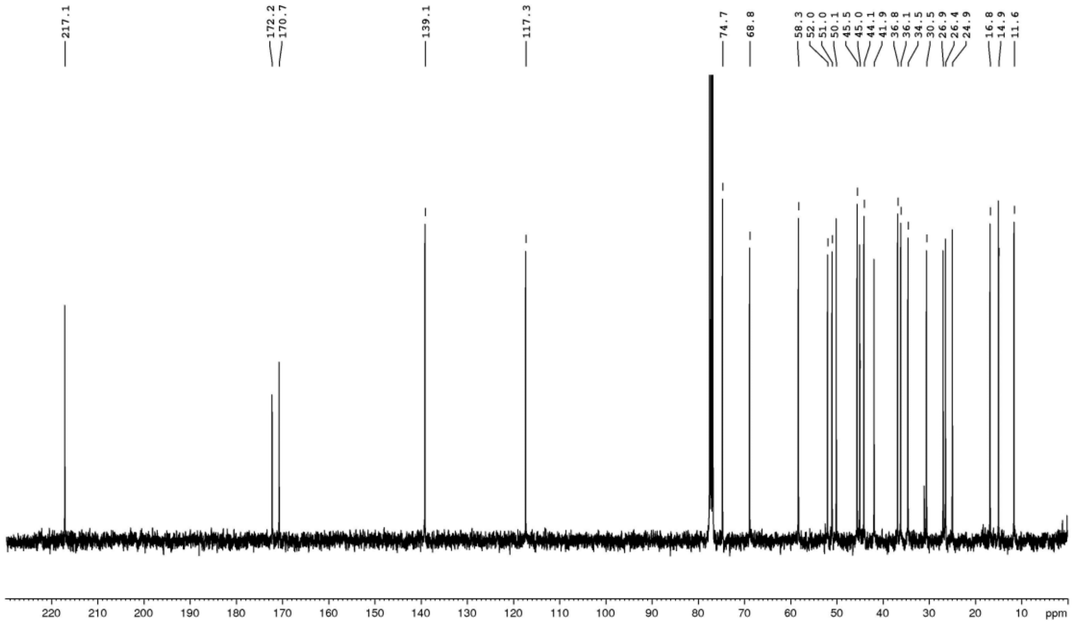

Figure S4 ^{1}H ($CDCl_3$, 400 MHz) and ^{13}C ($CDCl_3$, 100 MHz) NMR spectra for **16**.

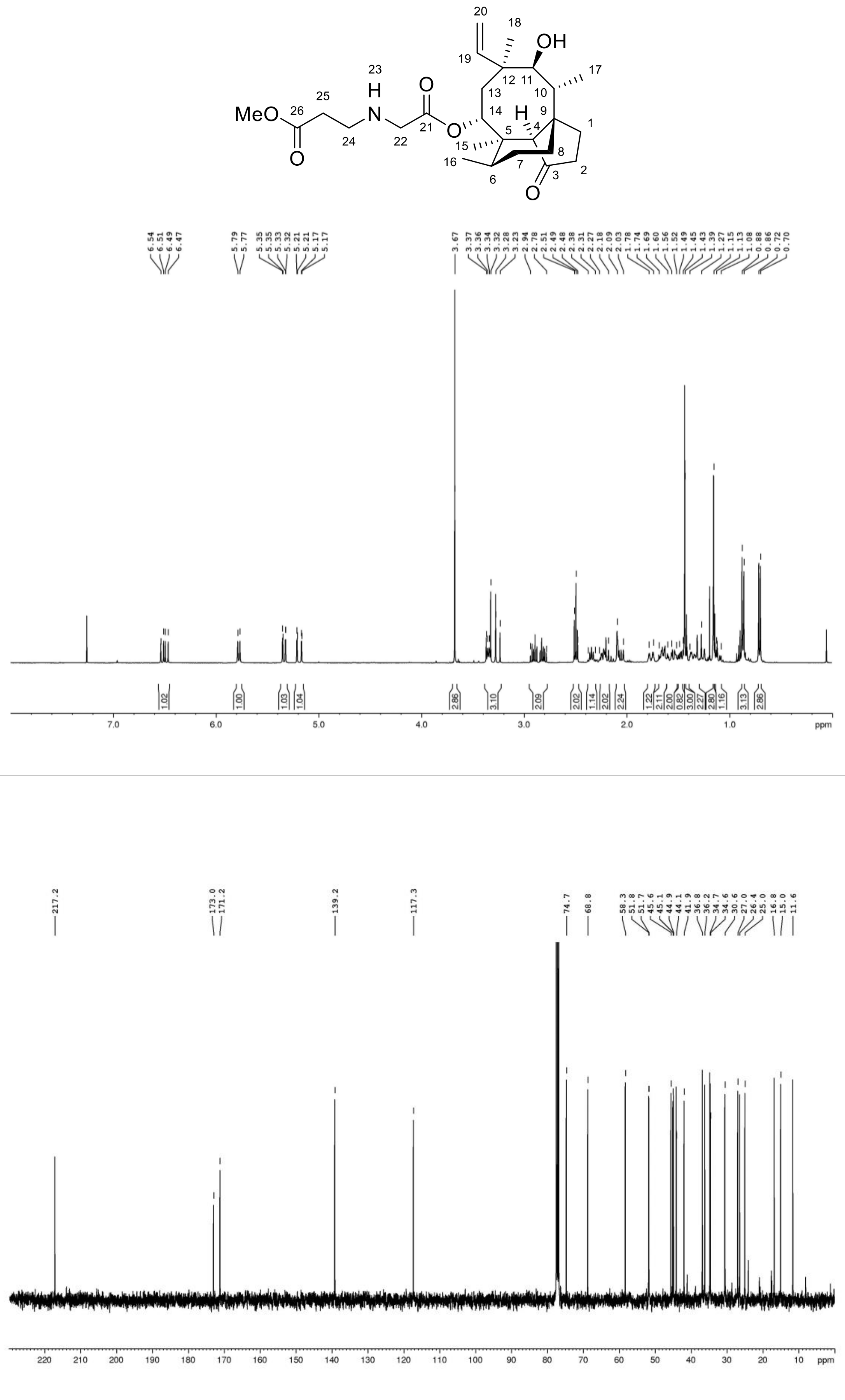

Figure S5 ^{1}H ($CDCl_3$, 400 MHz) and ^{13}C ($CDCl_3$, 100 MHz) NMR spectra for **17**.

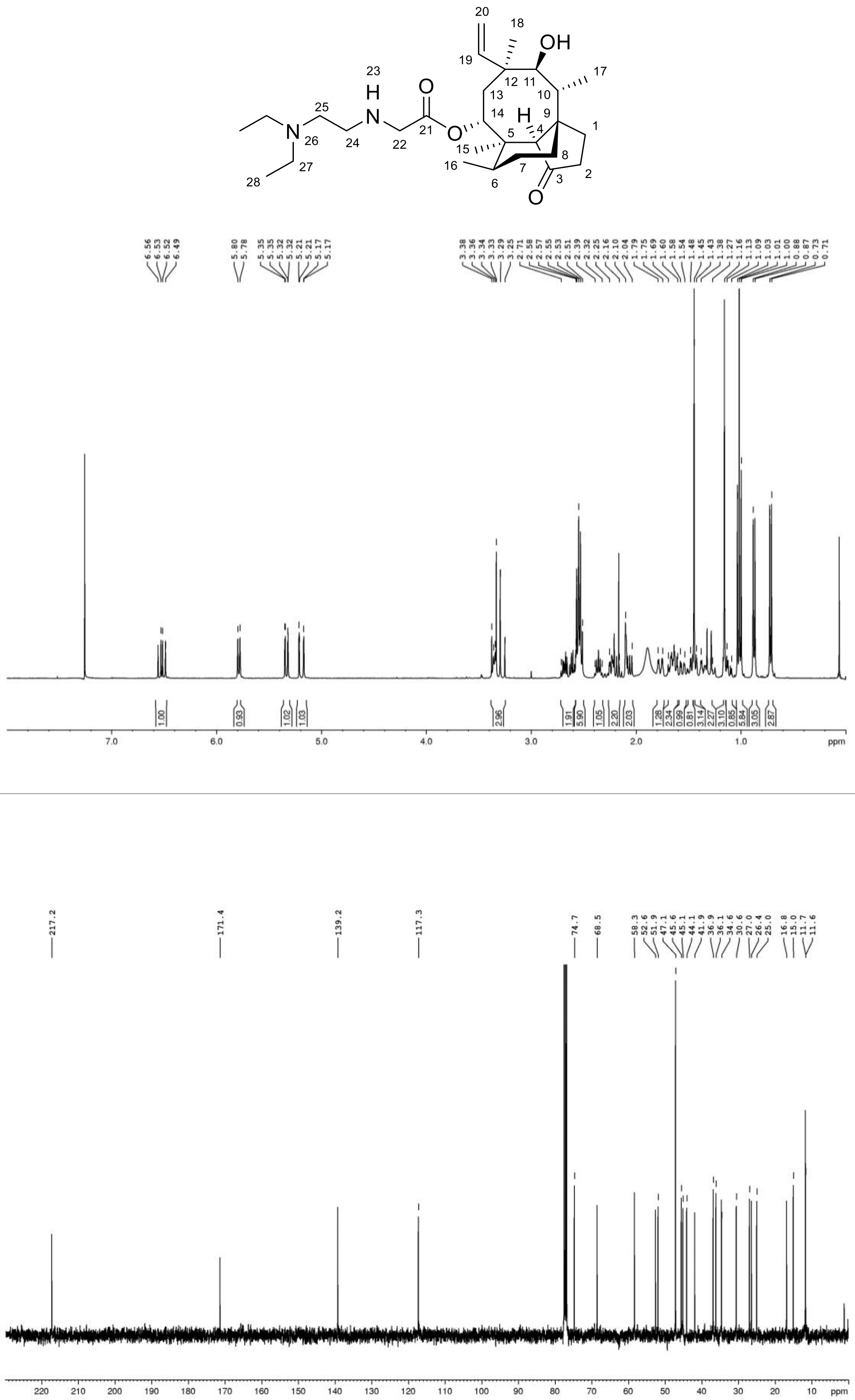

Figure S6 ^{1}H ($CDCl_3$, 400 MHz) and ^{13}C ($CDCl_3$, 100 MHz) NMR spectra for **18**.

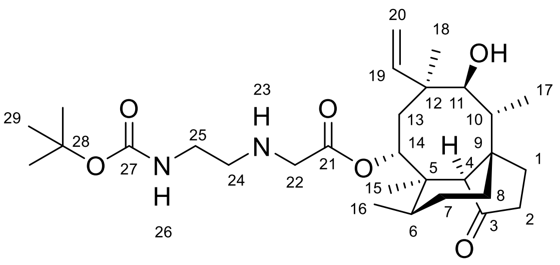

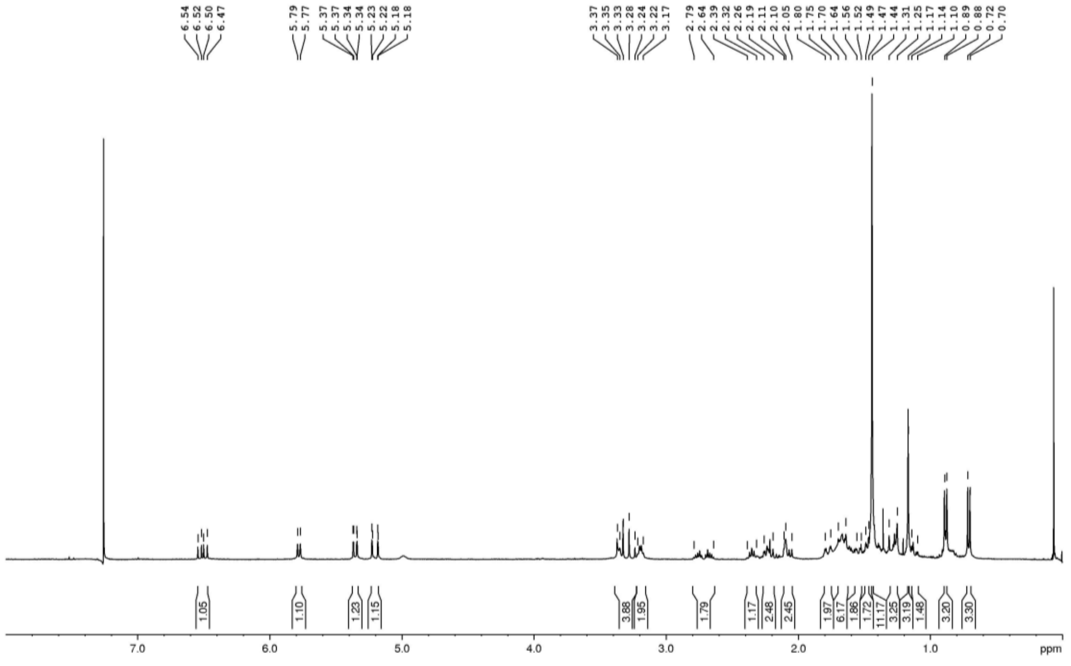

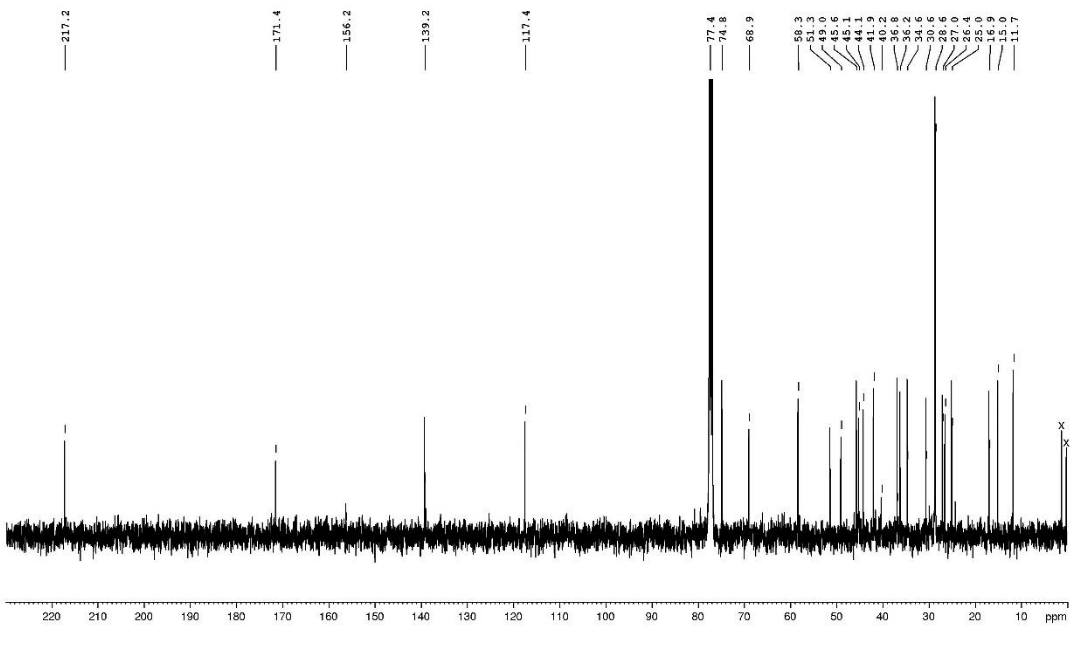

Figure S7 ^{1}H ($CDCl_3$, 400 MHz) and ^{13}C ($CDCl_3$, 100 MHz) NMR spectra for **19**.

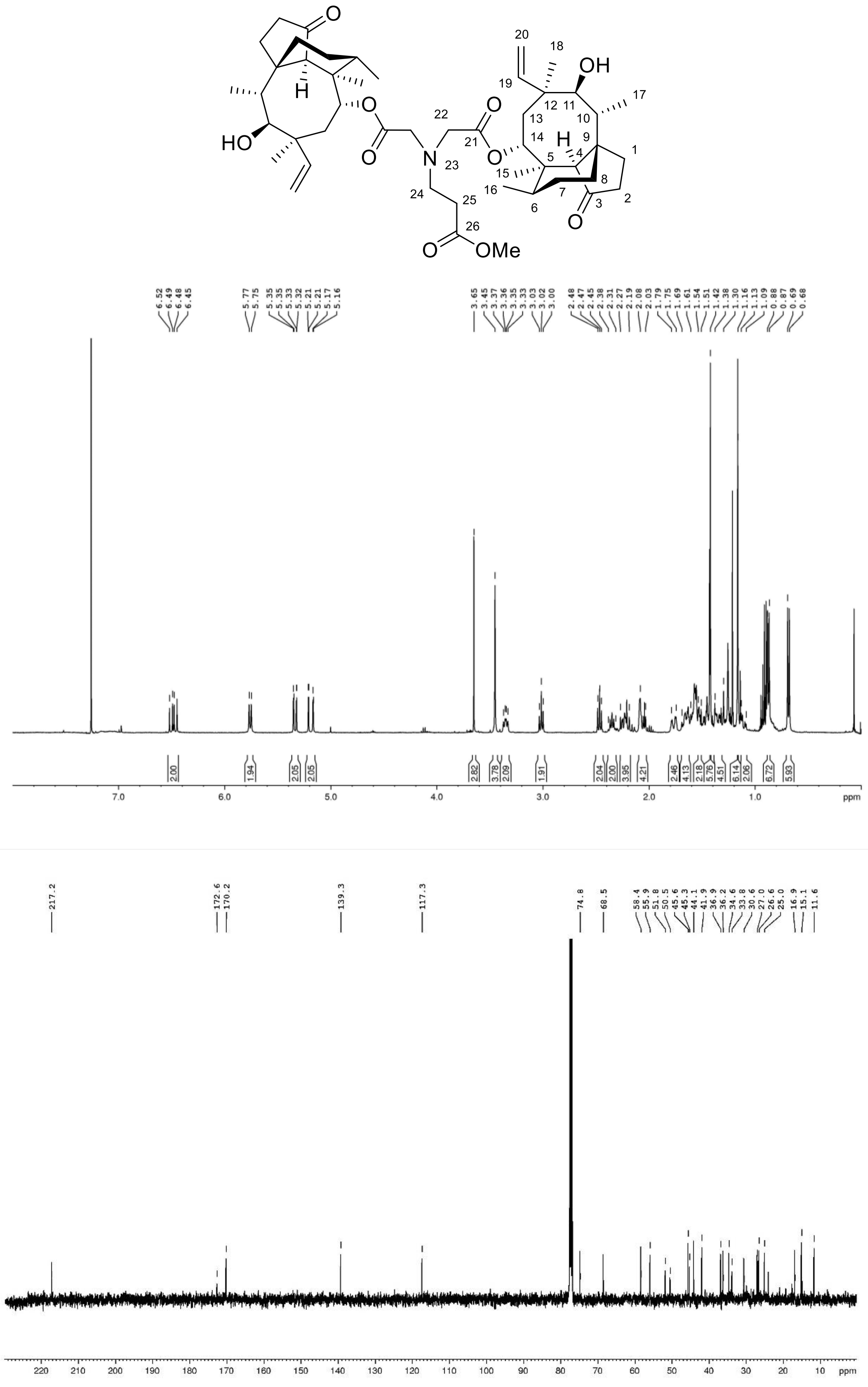

Figure S8 ^{1}H ($CDCl_3$, 400 MHz) and ^{13}C ($CDCl_3$, 100 MHz) NMR spectra for **20**.

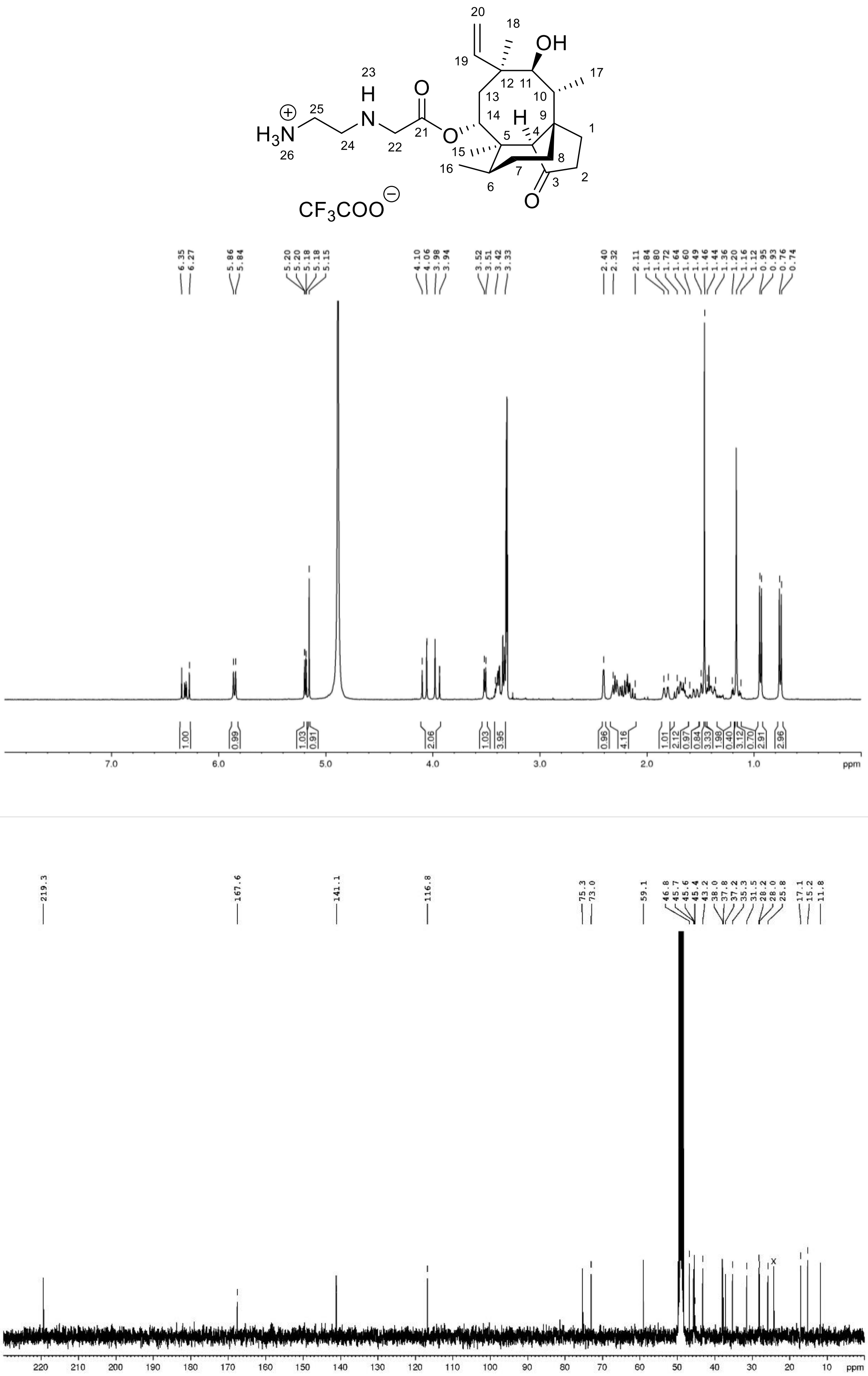

Figure S9 ^{1}H (CD_3OD, 400 MHz) and ^{13}C (CD_3OD, 100 MHz) NMR spectra for **22**.

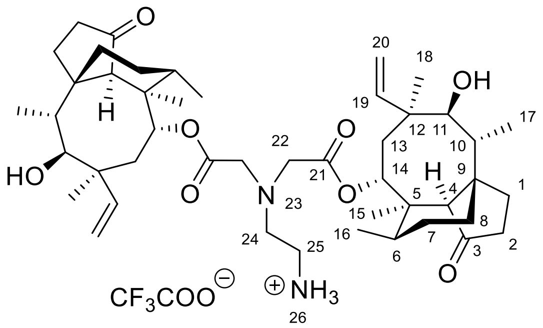

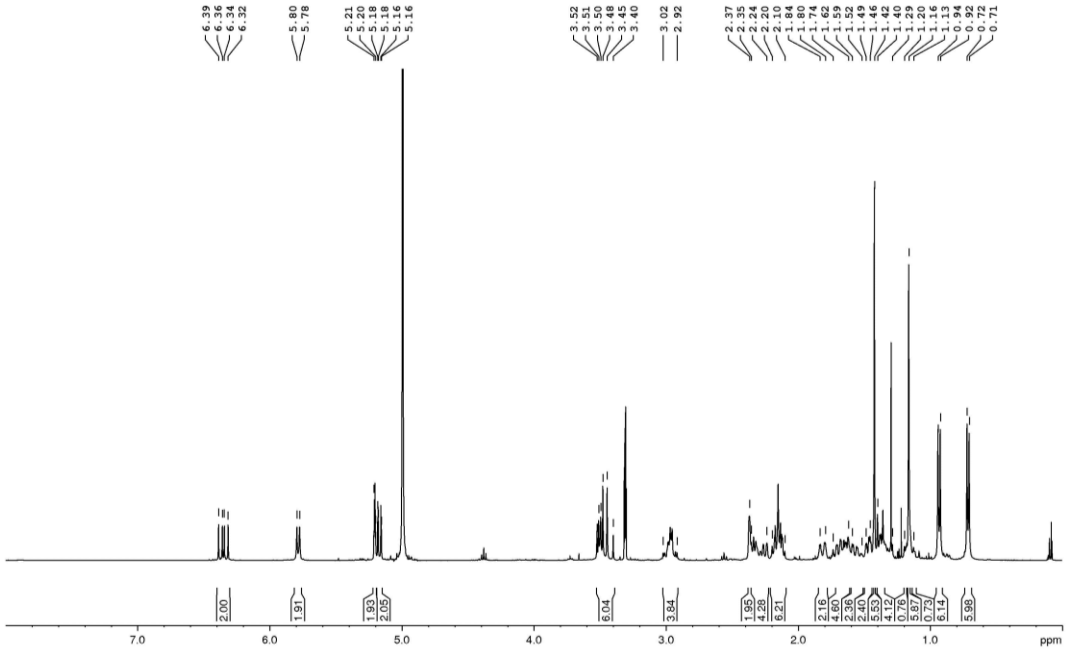

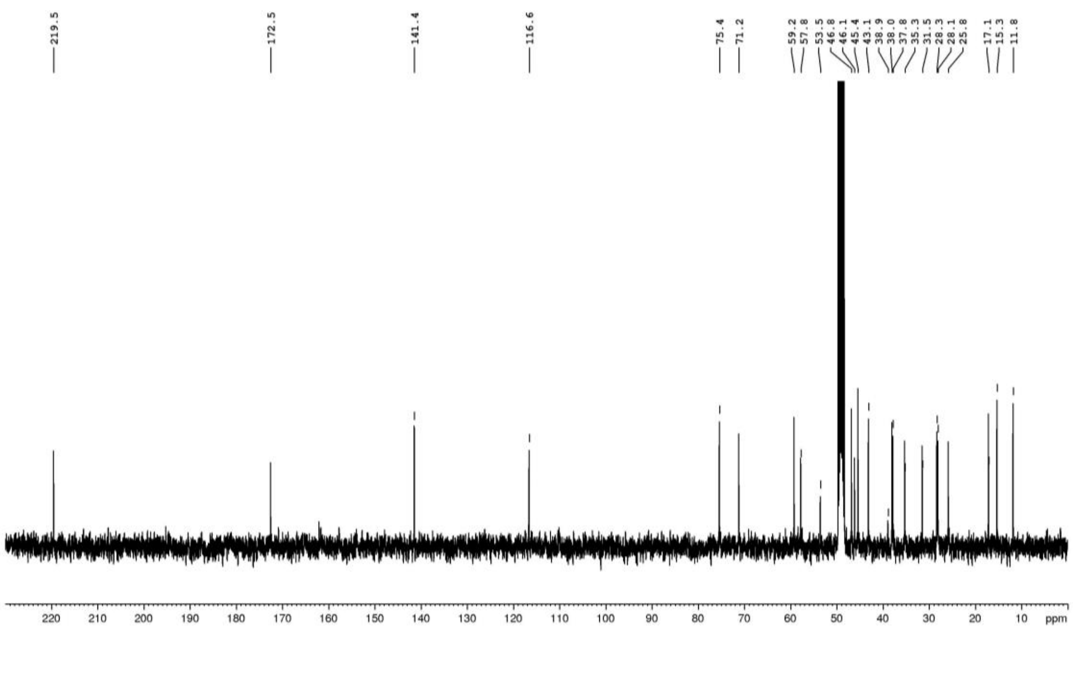

Figure S10 ^{1}H (CD_3OD, 400 MHz) and ^{13}C (CD_3OD, 100 MHz) NMR spectra for **23**.

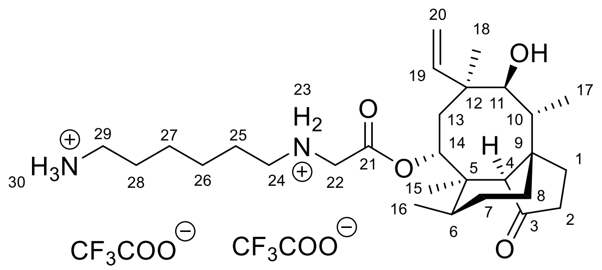

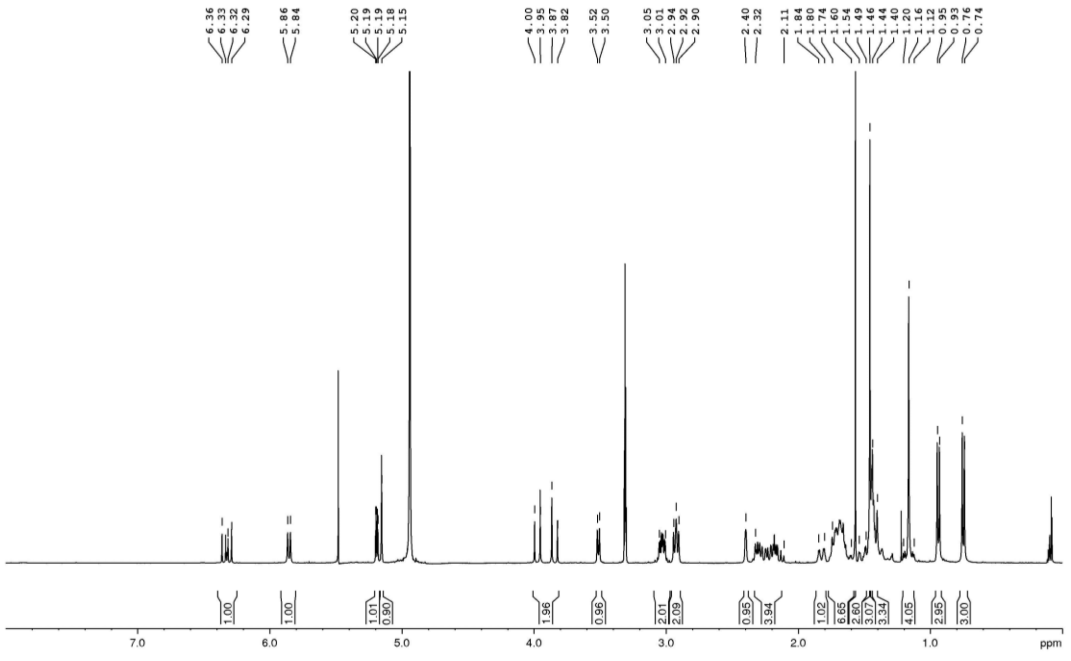

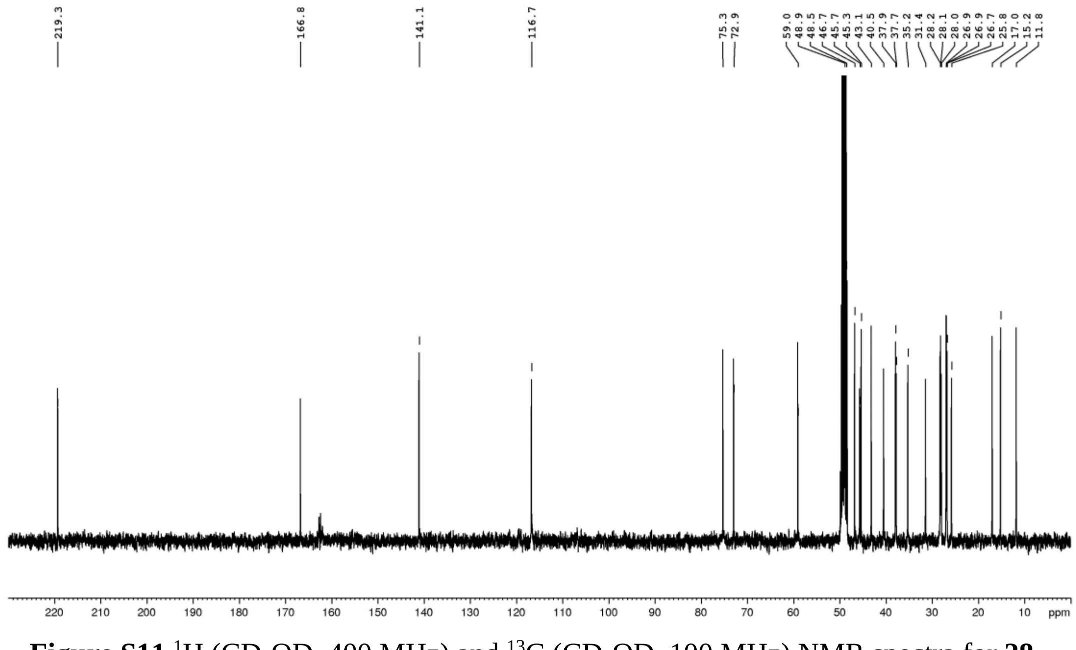

Figure S11 ^{1}H (CD_3OD, 400 MHz) and ^{13}C (CD_3OD, 100 MHz) NMR spectra for **28**.

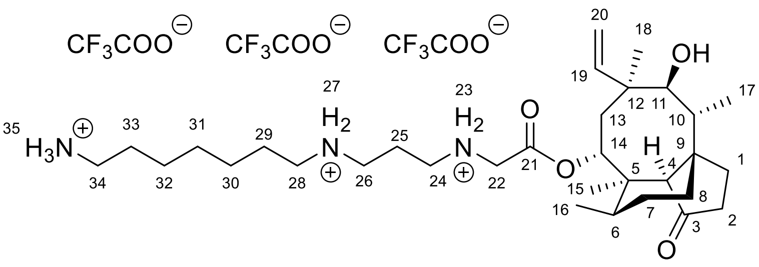

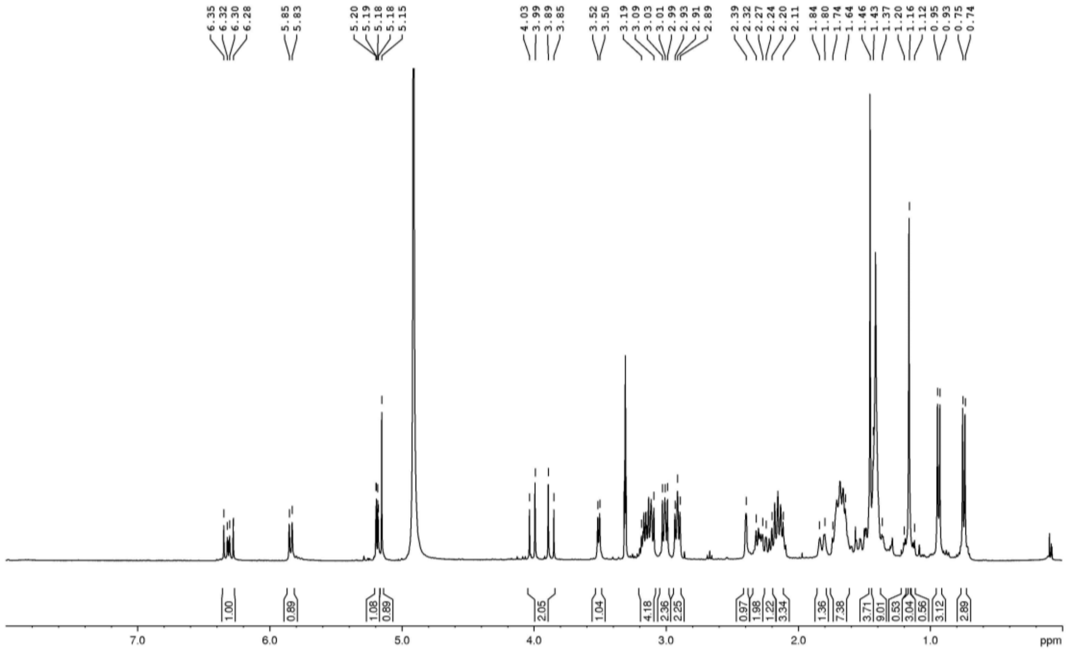

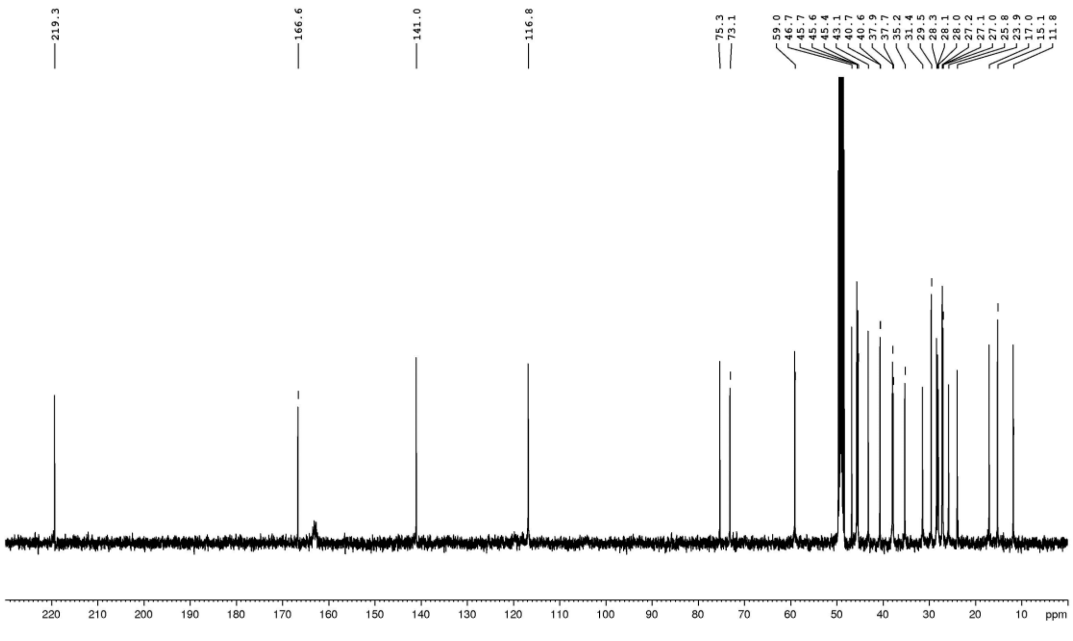

Figure S12 ^{1}H (CD3OD, 400 MHz) and ^{13}C (CD3OD, 100 MHz) NMR spectra for **29**.

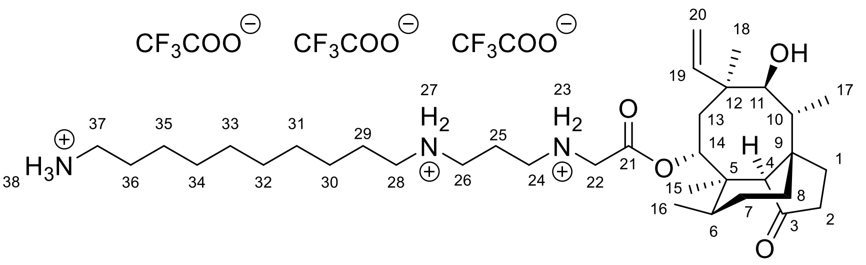

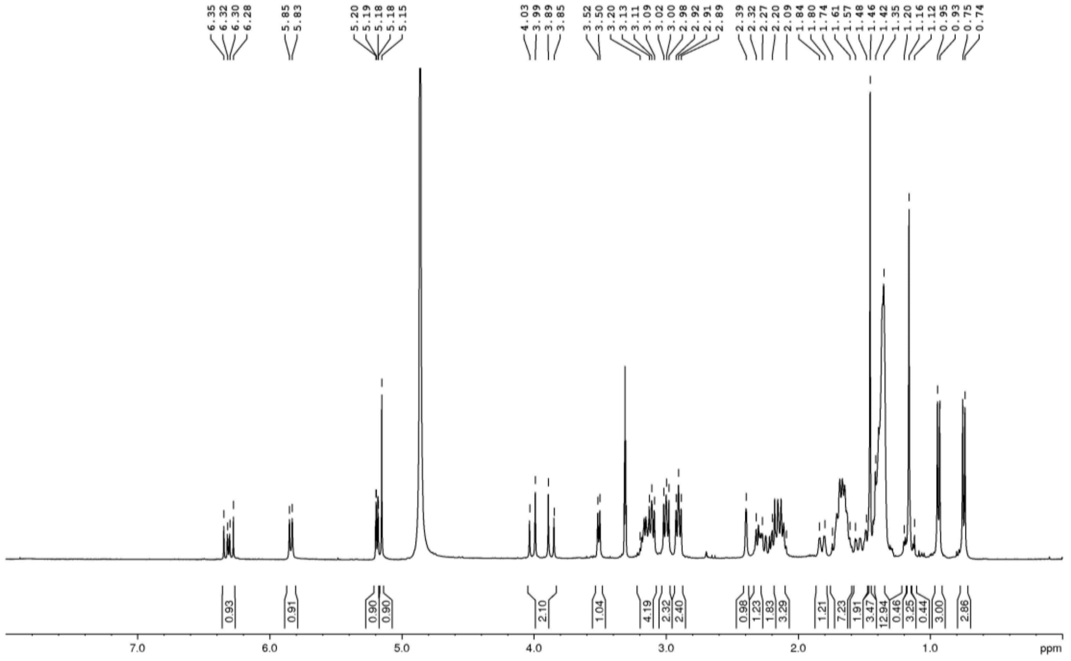

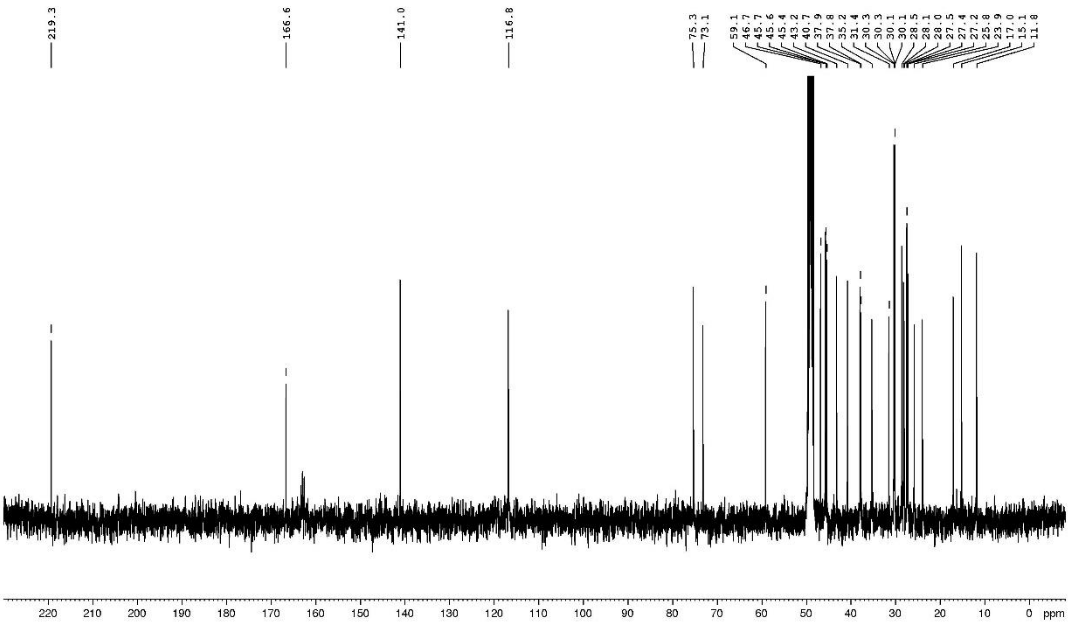

Figure S13 ^{1}H (CD_3OD, 400 MHz) and ^{13}C (CD_3OD, 100 MHz) NMR spectra for **30**.

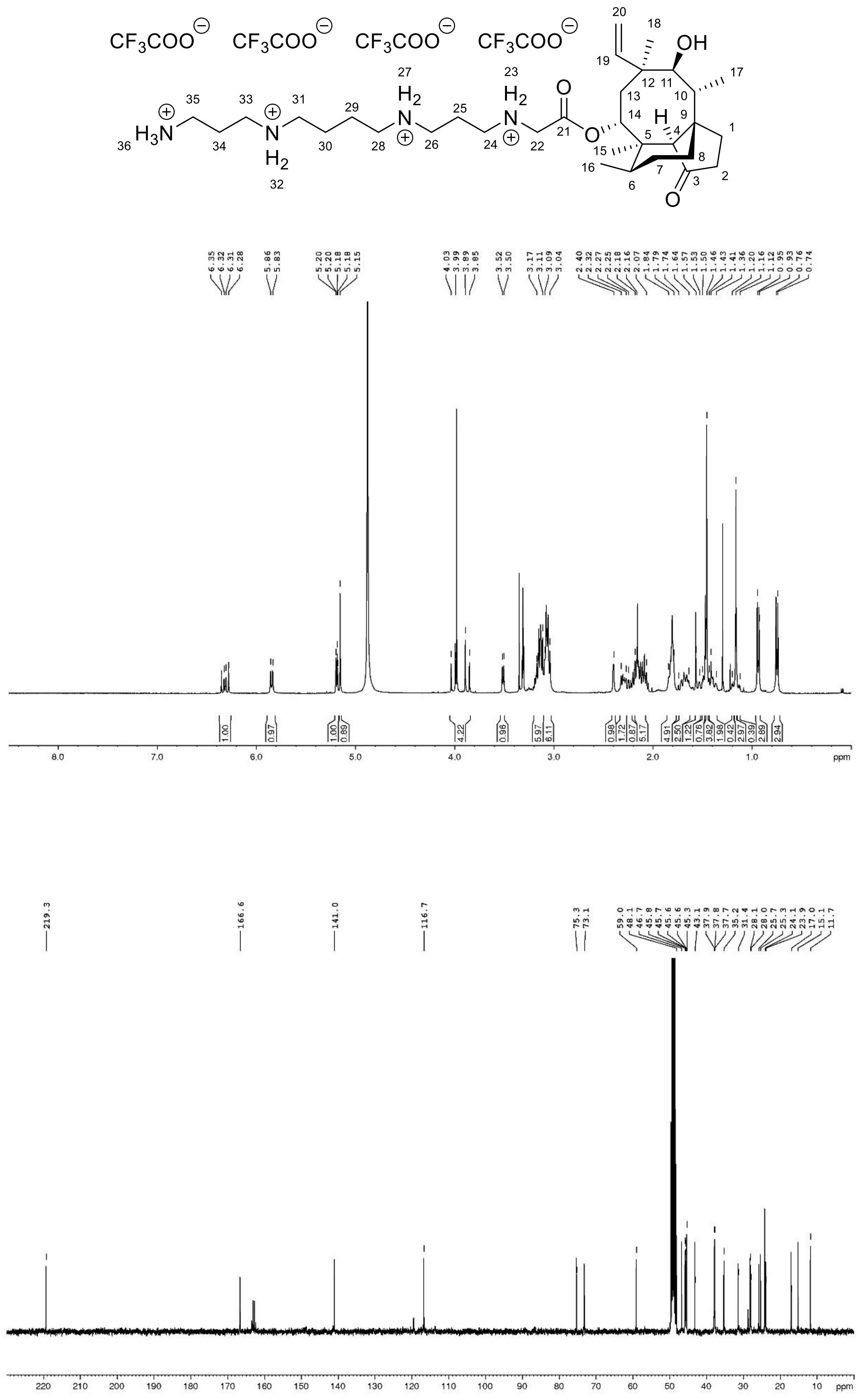

Figure S14 ^{1}H (CD_3OD, 400 MHz) and ^{13}C (CD_3OD, 100 MHz) NMR spectra for **31**.

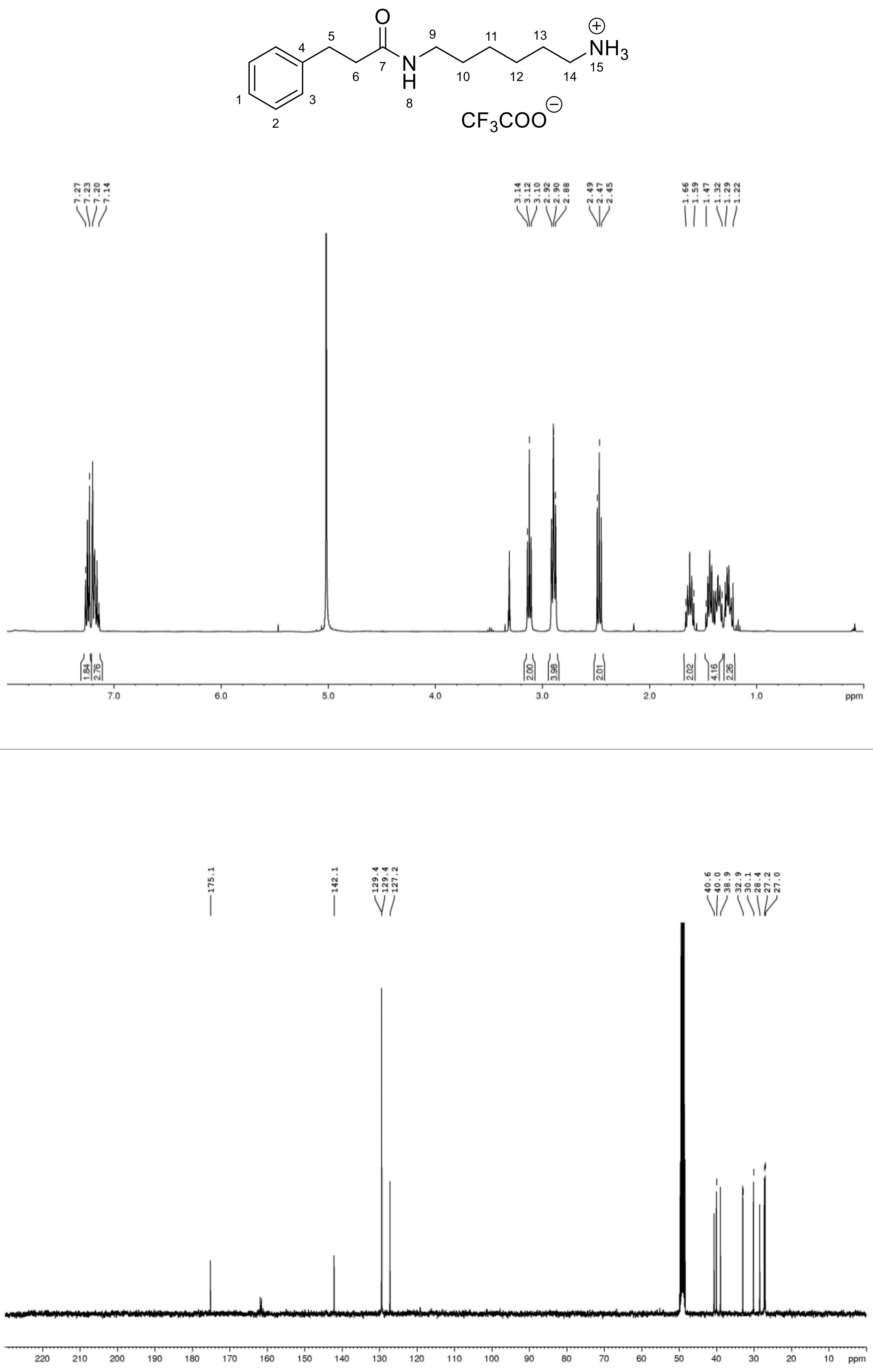

Figure S15 ^{1}H (CD_3OD, 400 MHz) and ^{13}C (CD_3OD, 100 MHz) NMR spectra for **36**.

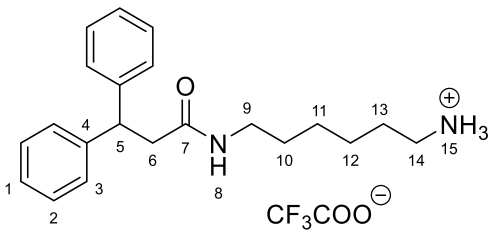

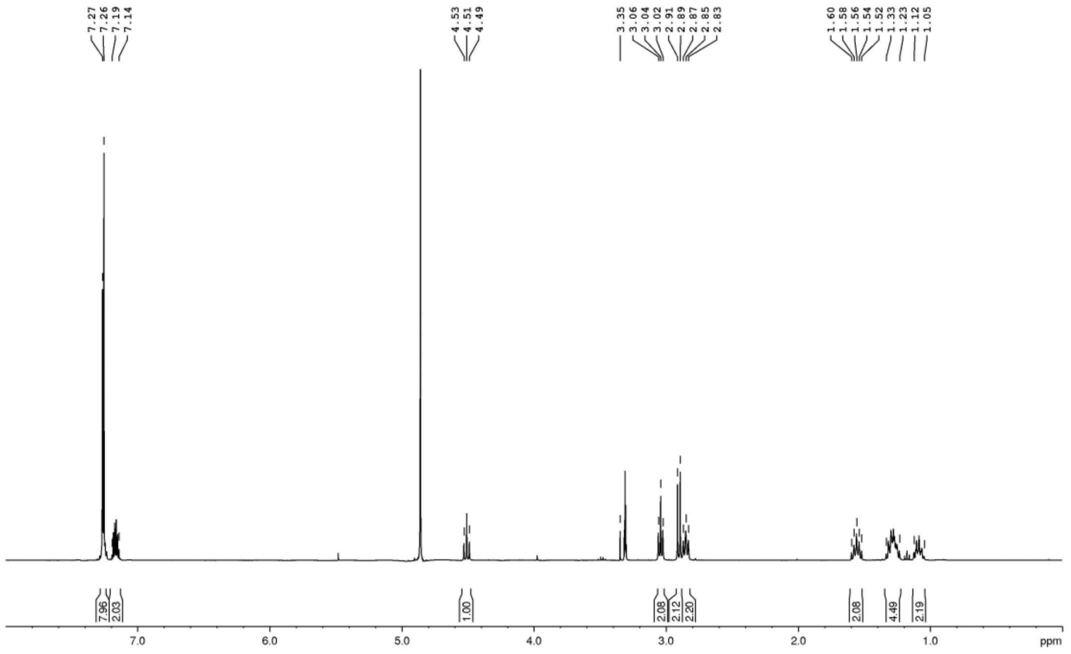

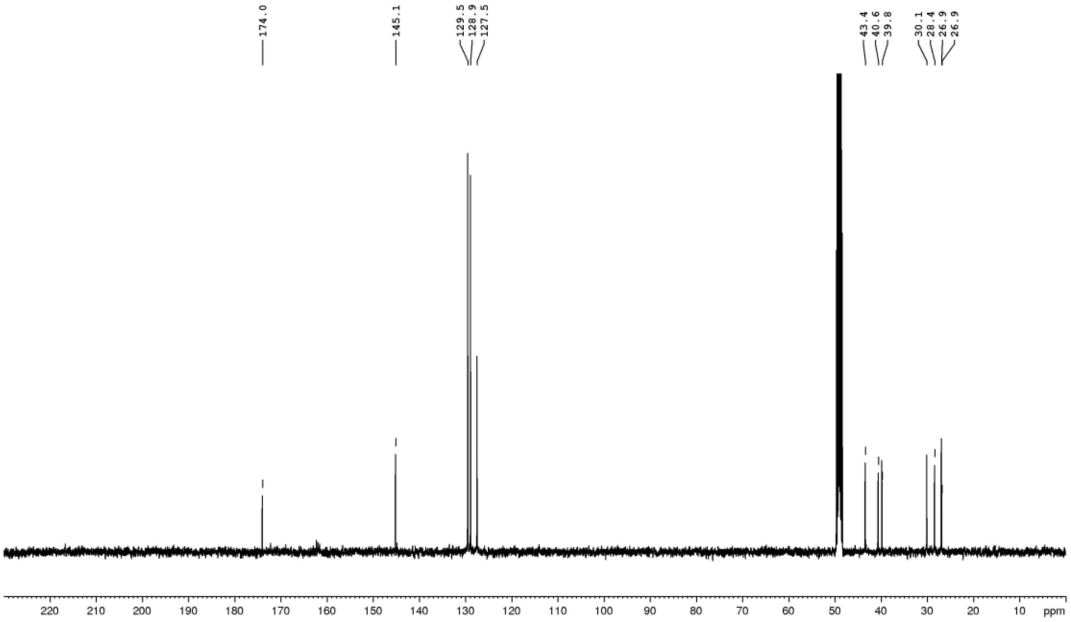

Figure S16 ^{1}H (CD_3OD, 400 MHz) and ^{13}C (CD_3OD, 100 MHz) NMR spectra for **37**.

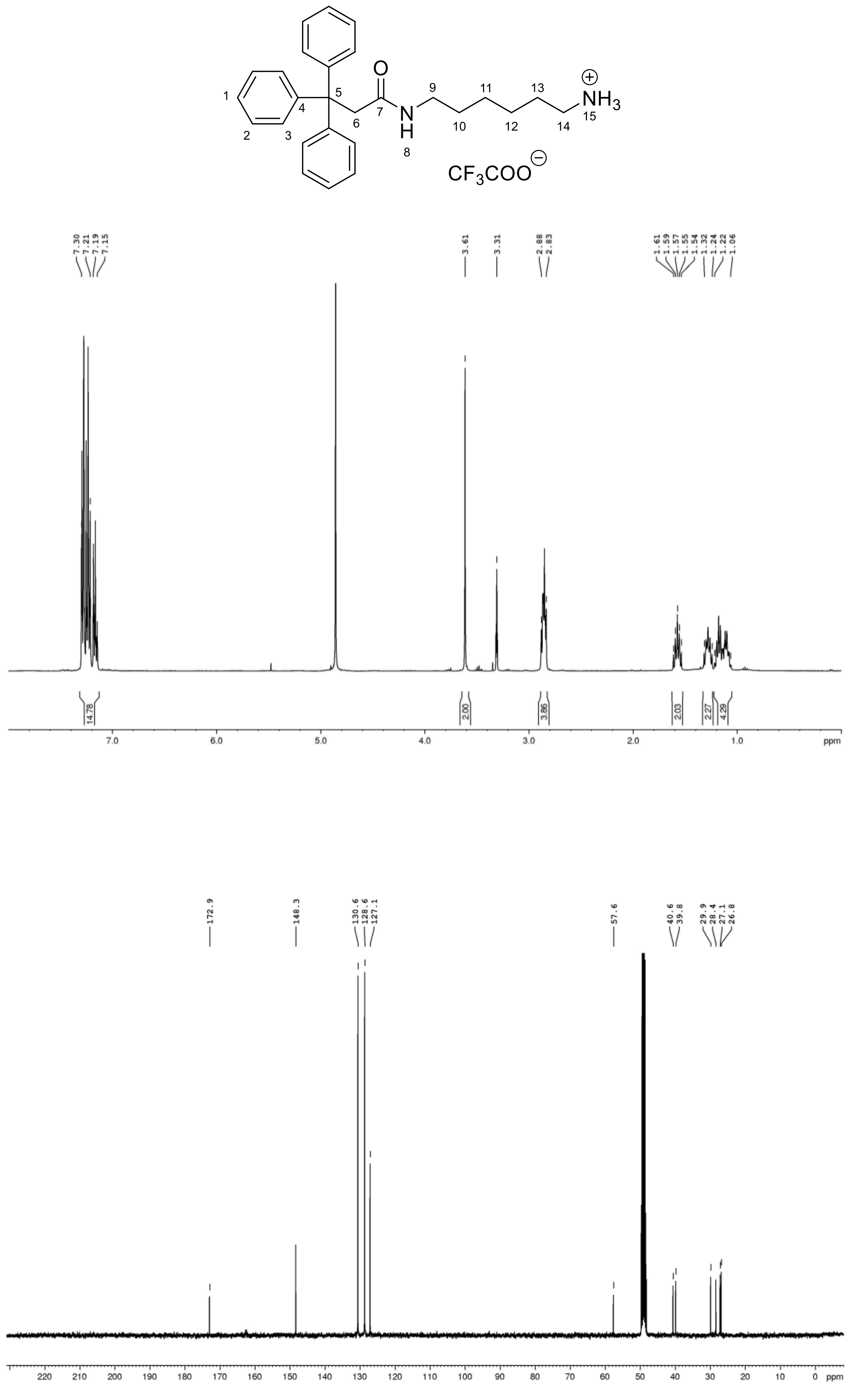

Figure S17 ^{1}H (CD_3OD, 400 MHz) and ^{13}C (CD_3OD, 100 MHz) NMR spectra for **38**.

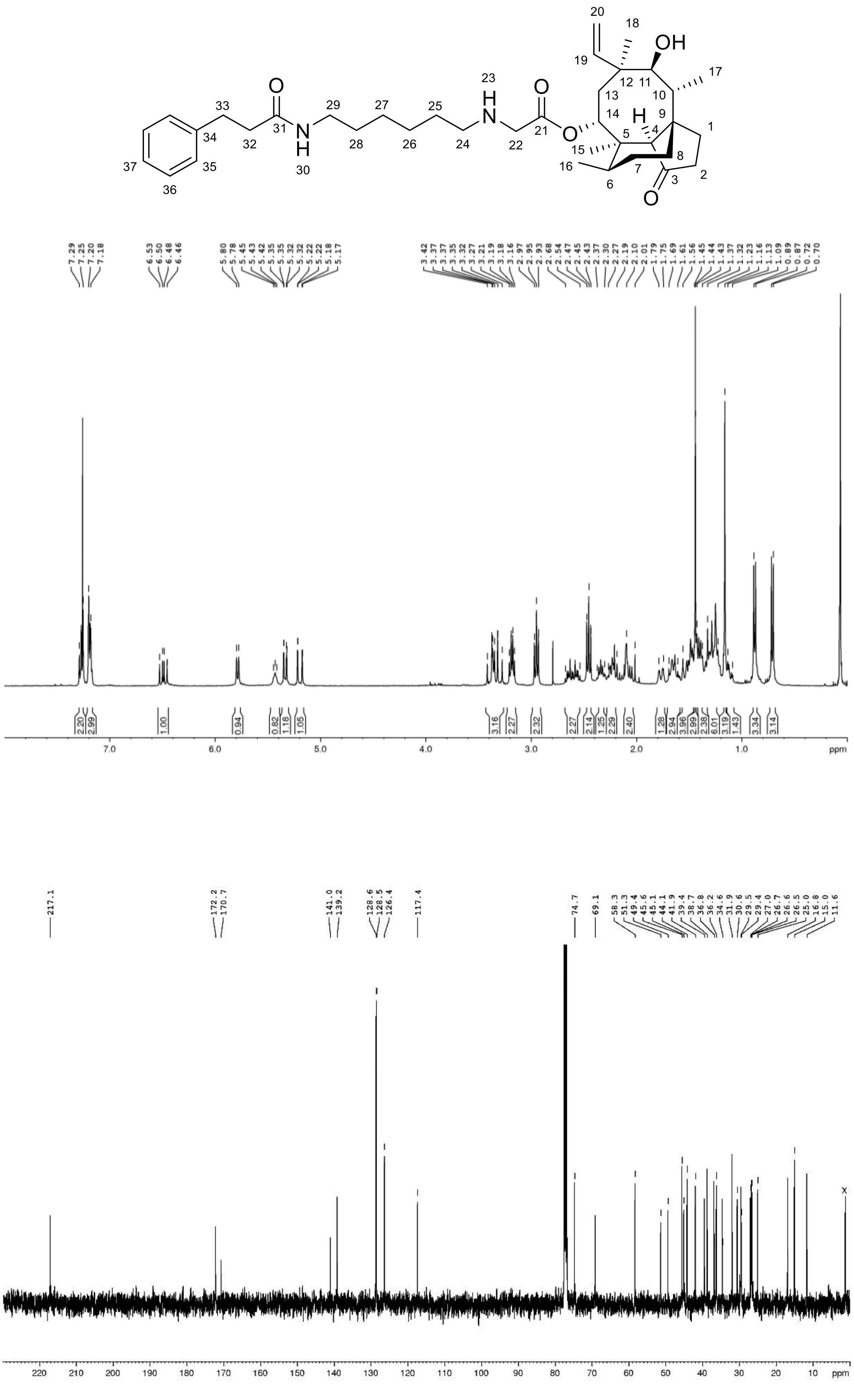

Figure S18 ^{1}H ($CDCl_3$, 400 MHz) and ^{13}C ($CDCl_3$, 100 MHz) NMR spectra for **39**.

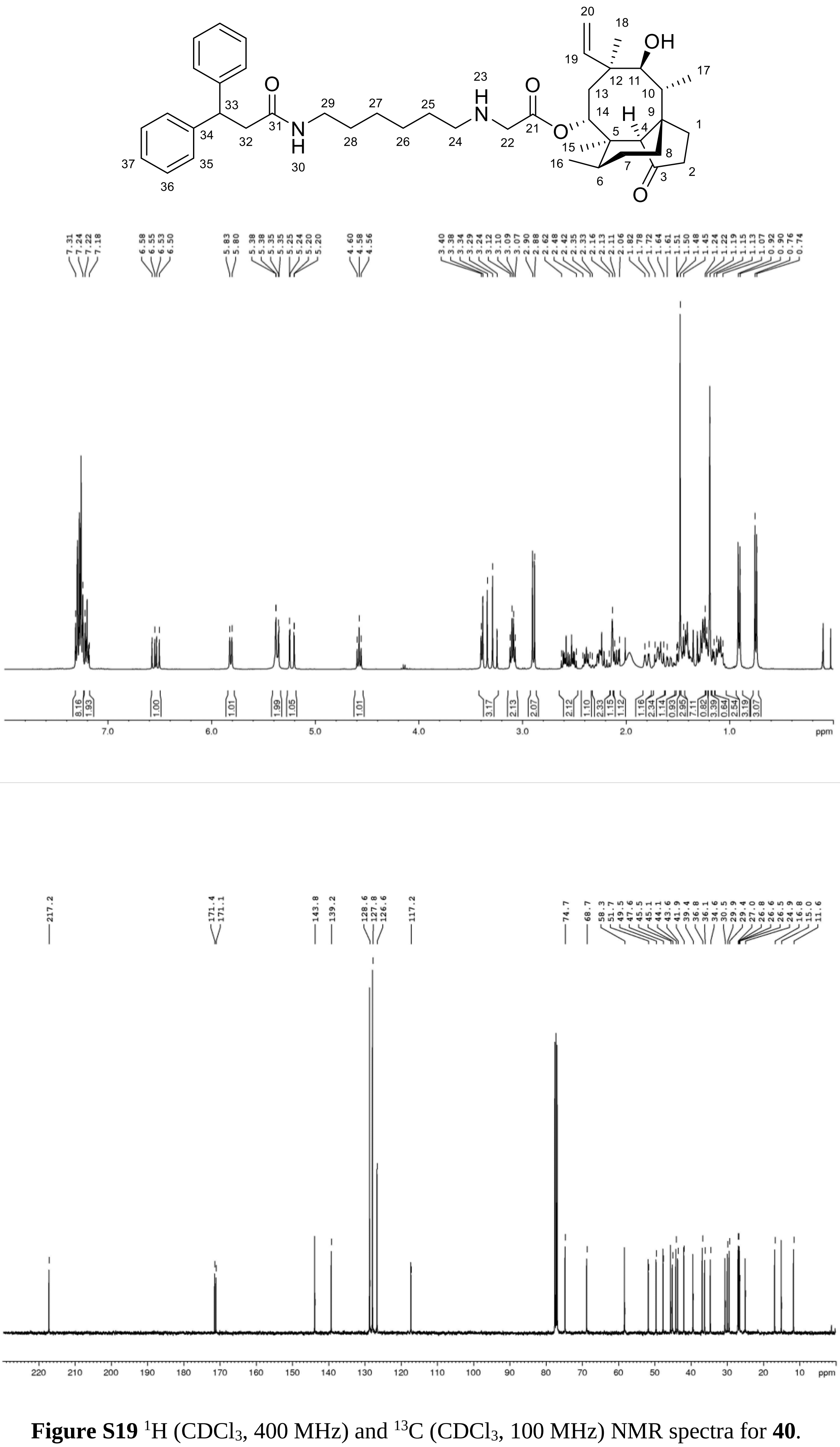

Figure S19 ^{1}H ($CDCl_3$, 400 MHz) and ^{13}C ($CDCl_3$, 100 MHz) NMR spectra for **40**.

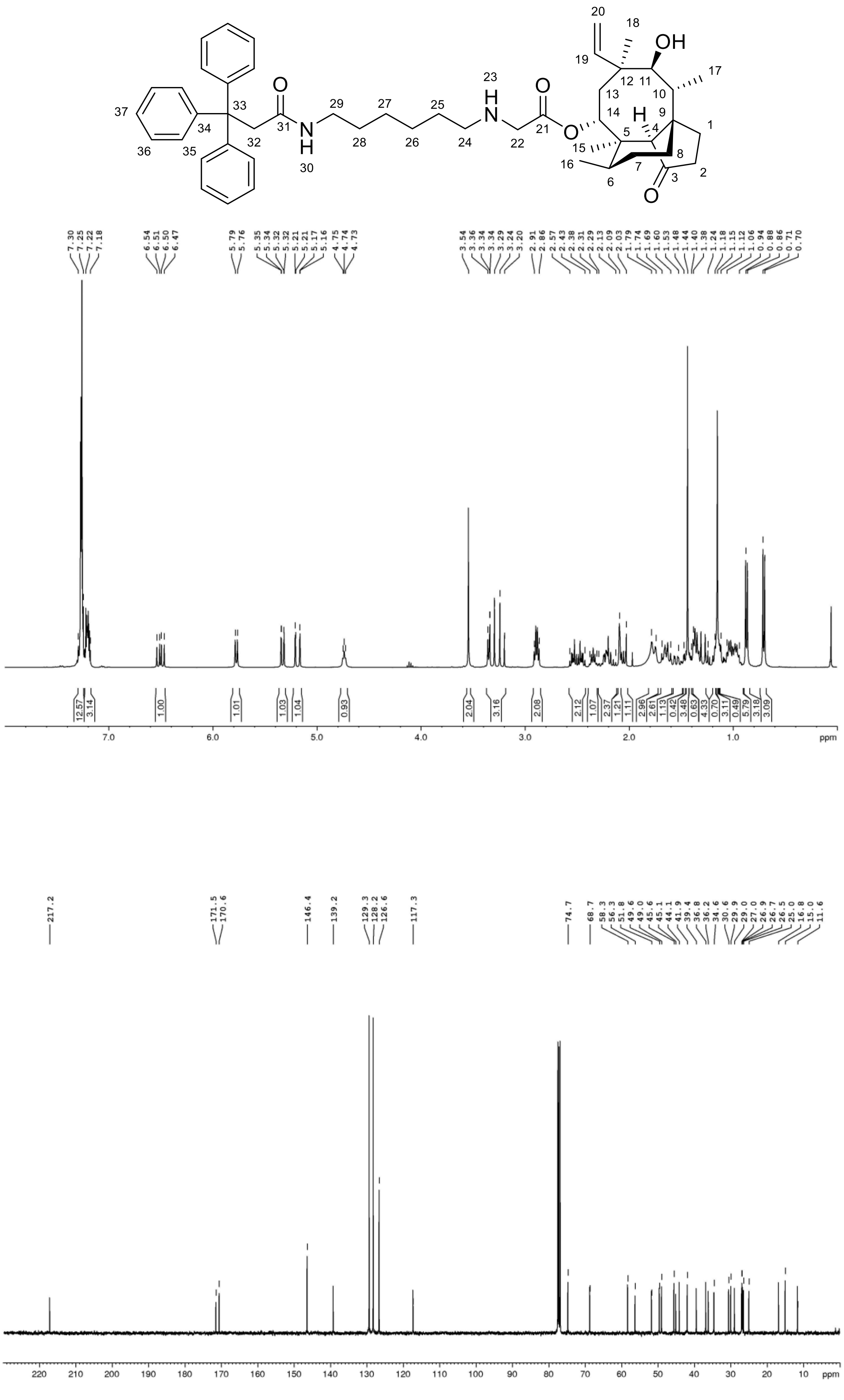

Figure S20 ^{1}H ($CDCl_3$, 400 MHz) and ^{13}C ($CDCl_3$, 100 MHz) NMR spectra for **41**.

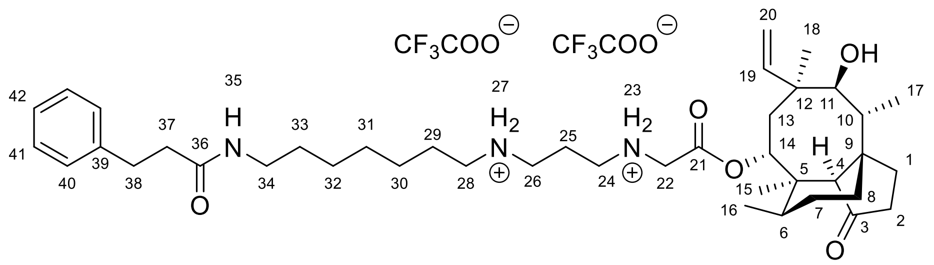

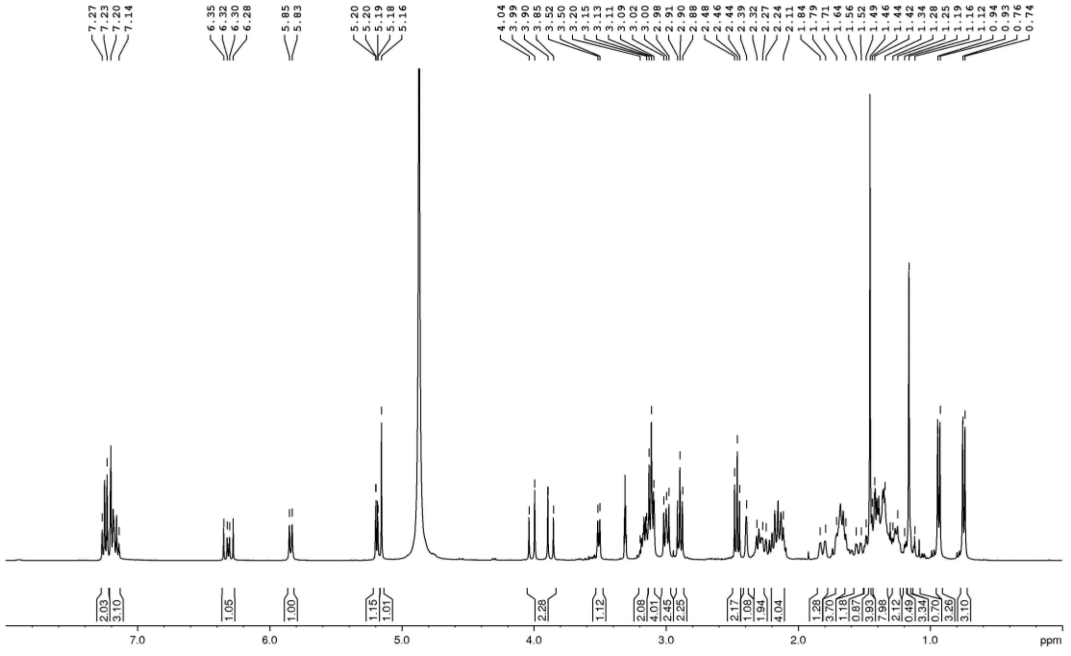

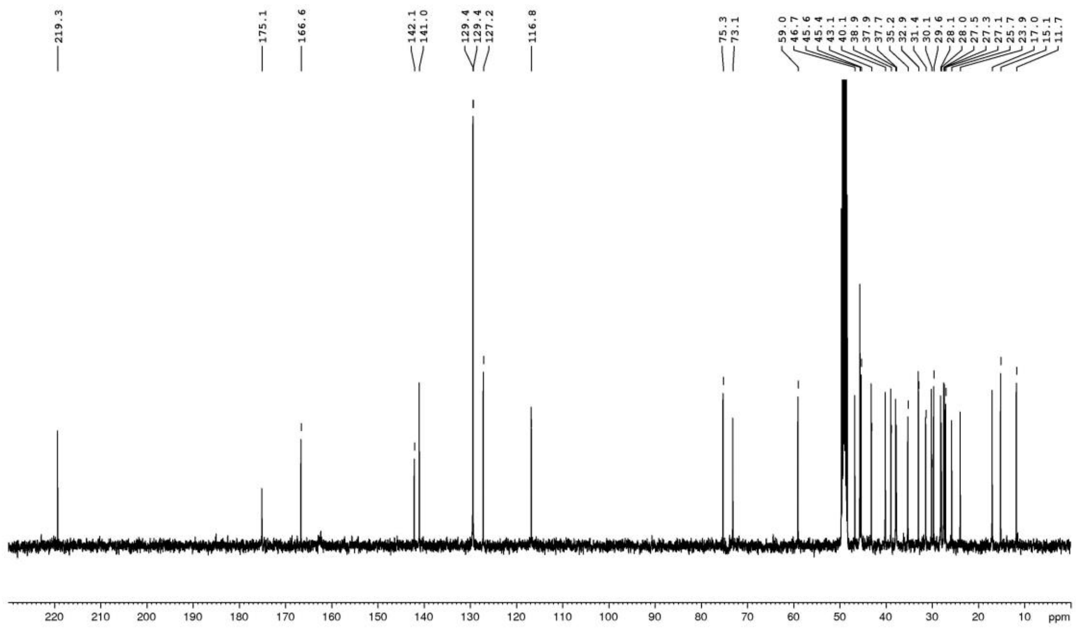

Figure S21 ^{1}H (CD_3OD, 400 MHz) and ^{13}C (CD_3OD, 100 MHz) NMR spectra for **42**.

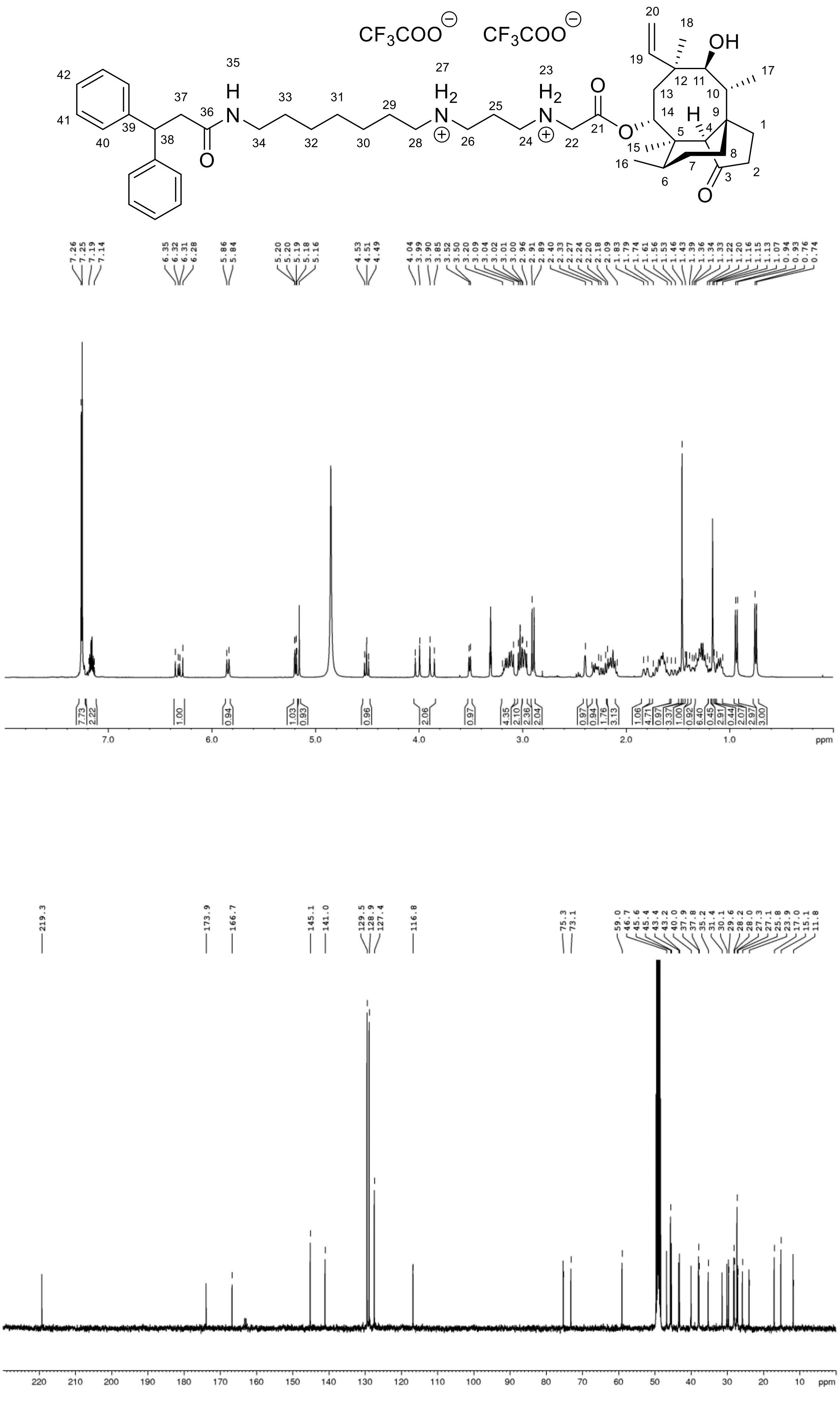

Figure S22 ^{1}H (CD_3OD, 400 MHz) and ^{13}C (CD_3OD, 100 MHz) NMR spectra for **43**.

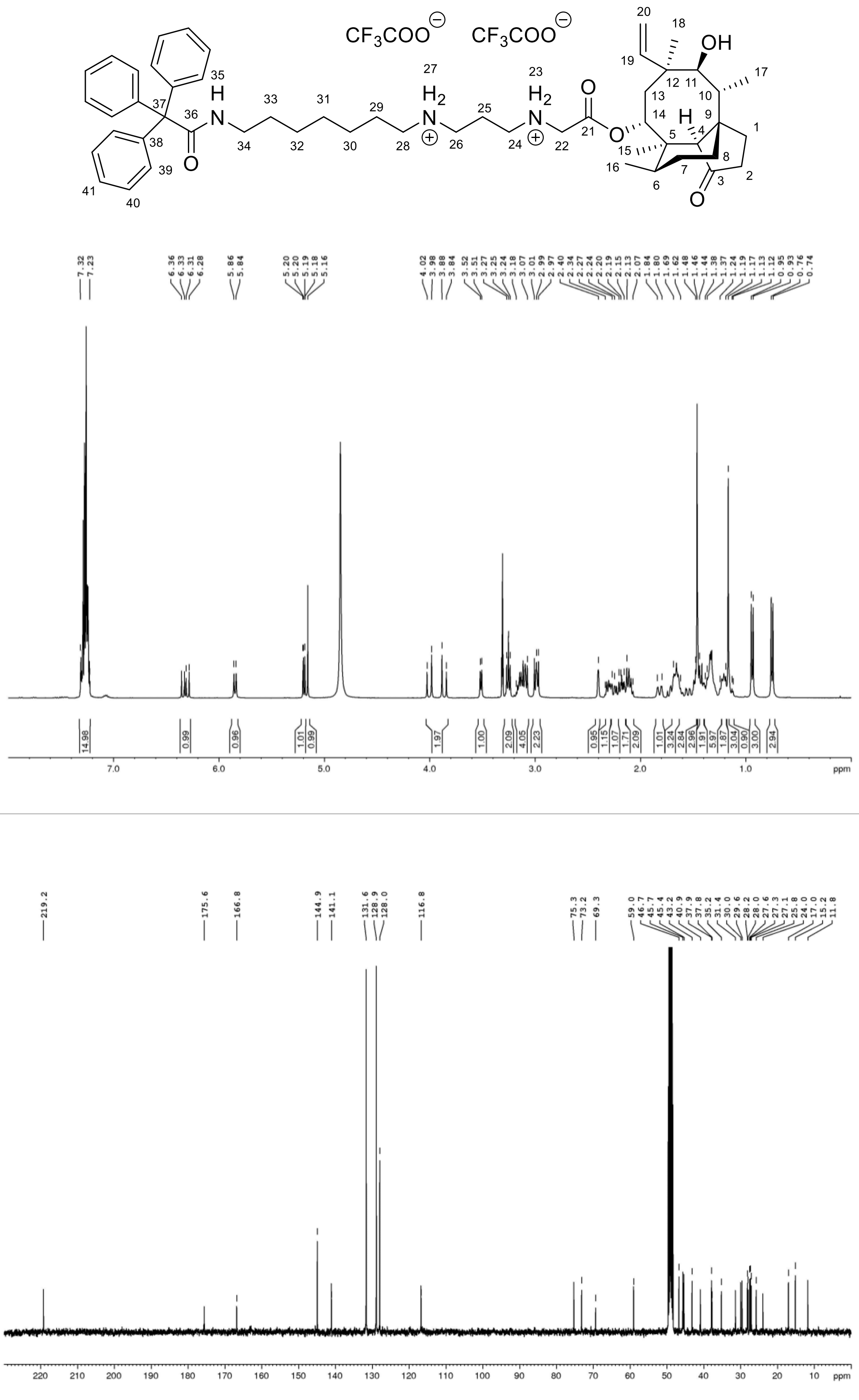

Figure S23 ^{1}H (CD_3OD, 400 MHz) and ^{13}C (CD_3OD, 100 MHz) NMR spectra for **44**.

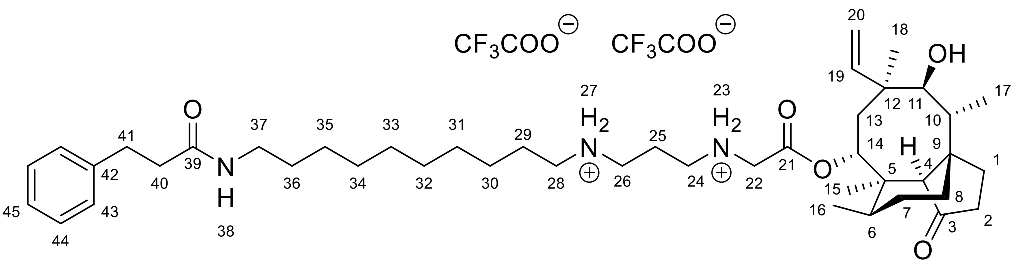

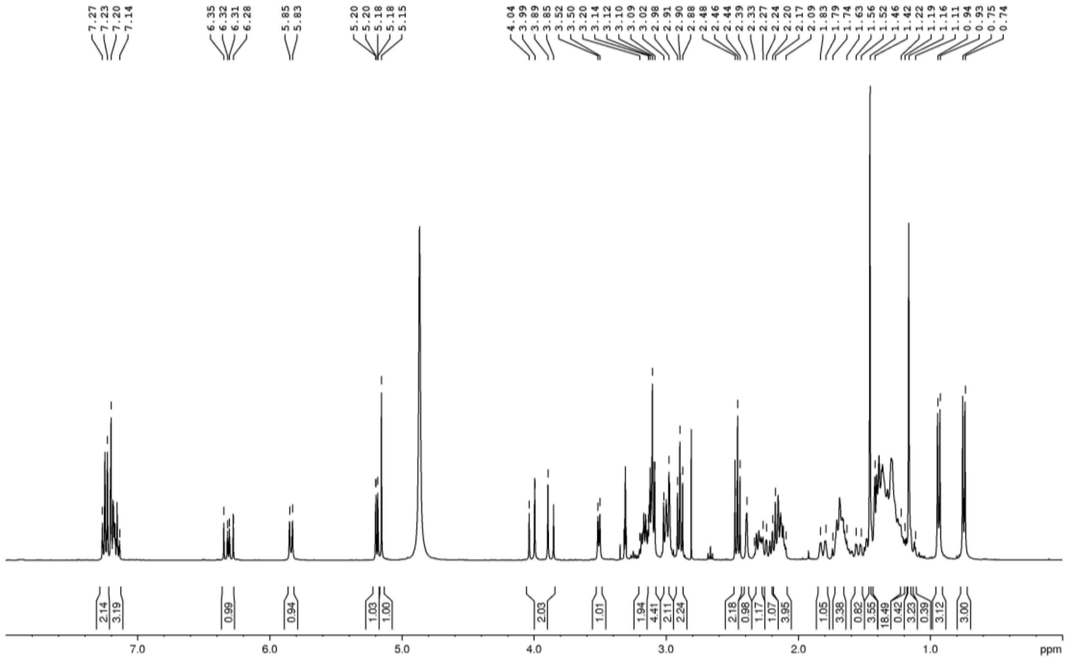

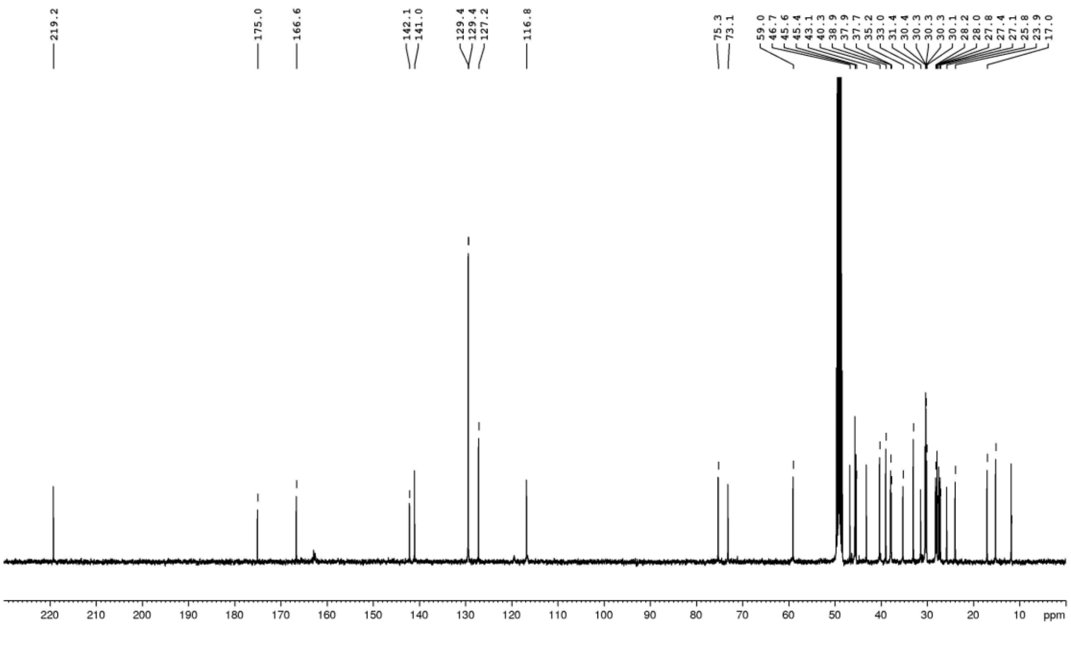

Figure S24 ^{1}H (CD_3OD, 400 MHz) and ^{13}C (CD_3OD, 100 MHz) NMR spectra for **45**.

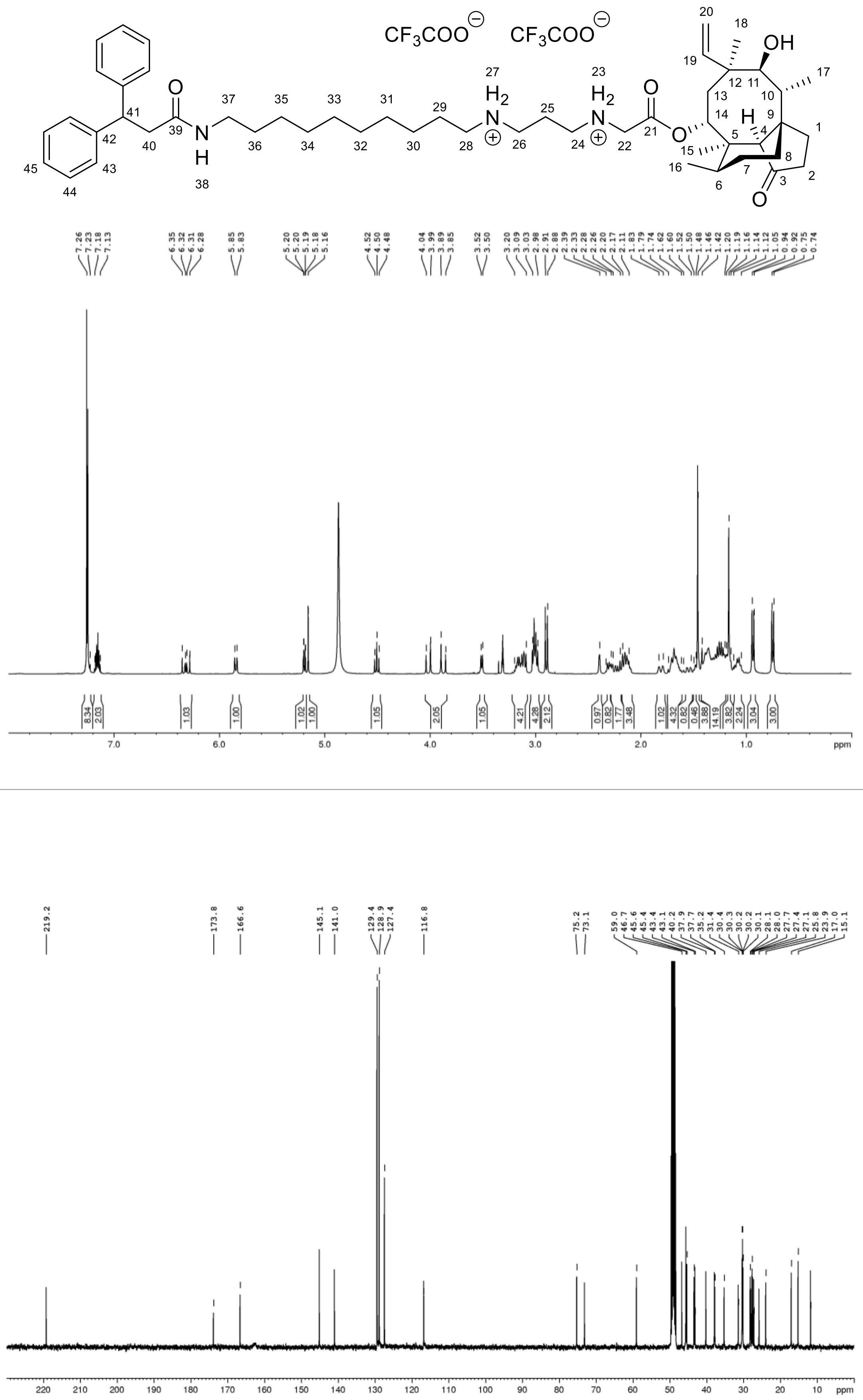

Figure S25 ^{1}H (CD_3OD, 400 MHz) and ^{13}C (CD_3OD, 100 MHz) NMR spectra for **46**.

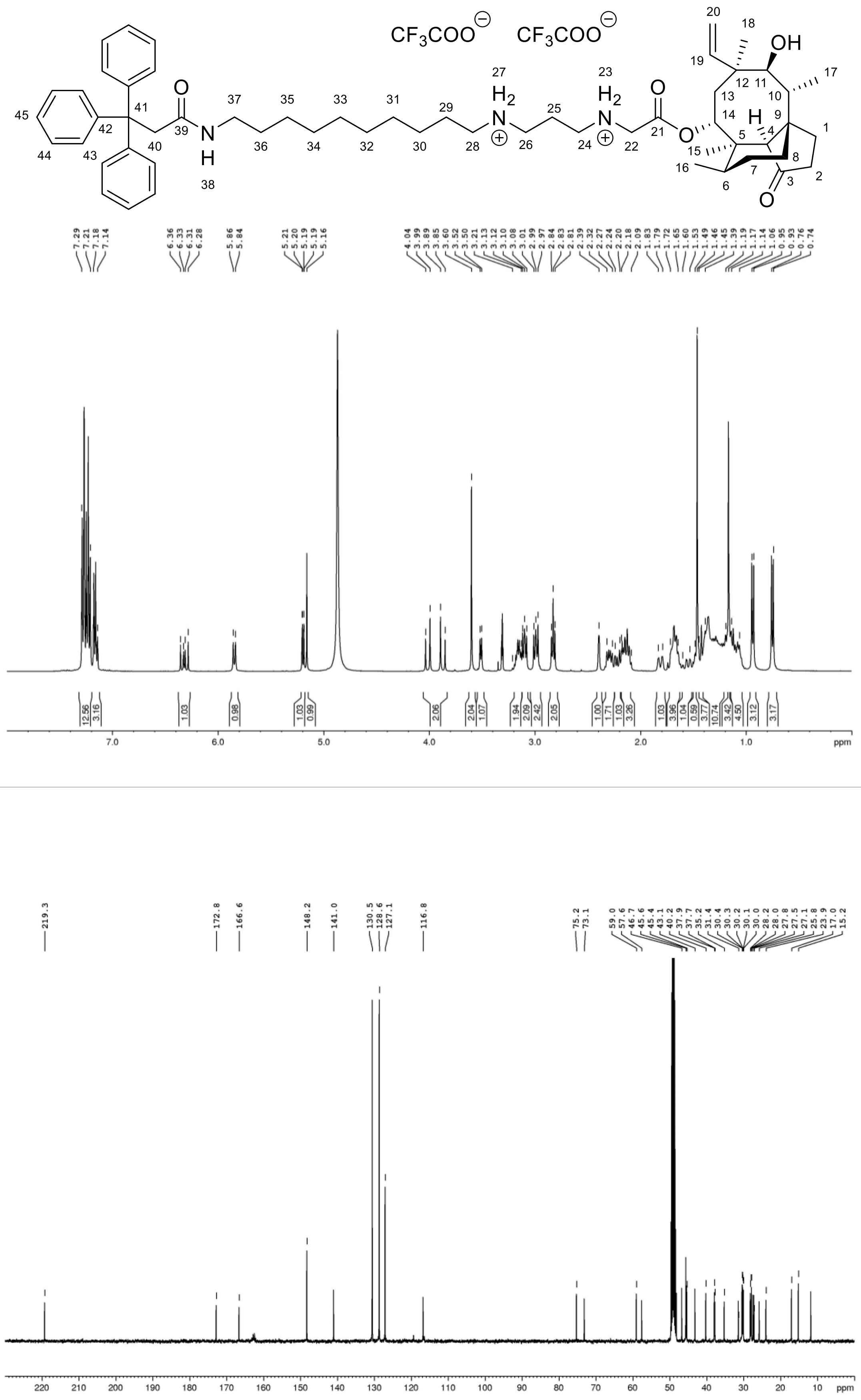

Figure S26 ^{1}H (CD_3OD, 400 MHz) and ^{13}C (CD_3OD, 100 MHz) NMR spectra for **47**.

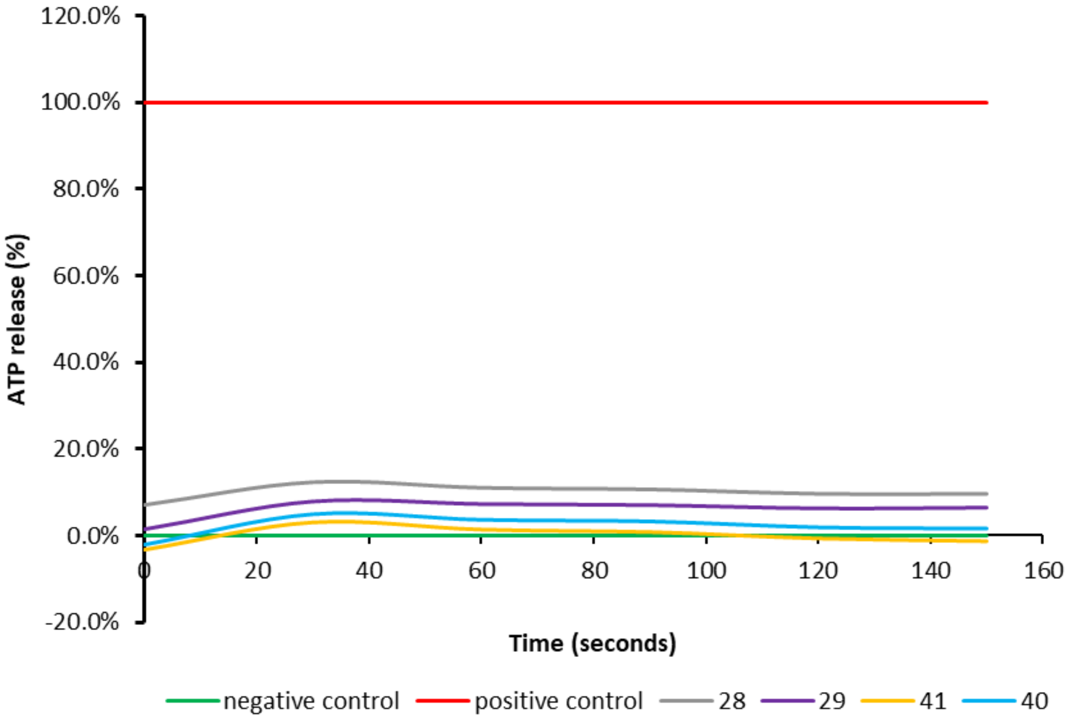

Figure S27. ATP release in *S. aureus* ATCC 25923 exhibited by selected compounds (**28**, **29**, **40** and **41**) as determined using an ATP efflux assay. Squalamine (100 µg/mL) was used as the positive control and water as the negative control. Compounds were tested at a fixed concentration of 100 µg/mL and results reported as percentage (%) relative to positive control.

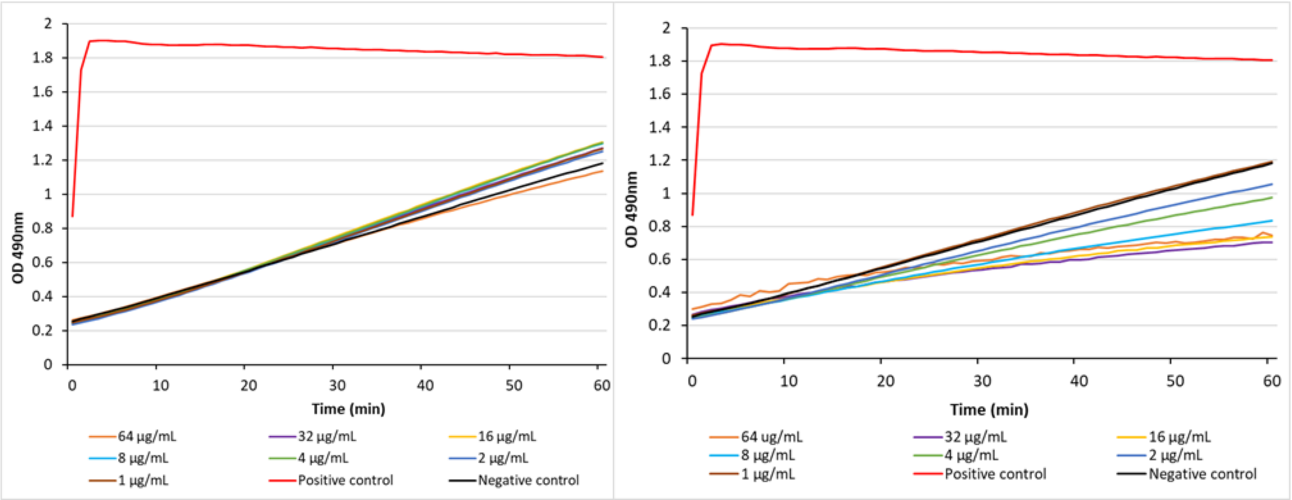

Figure S28. The ability of tiamulin (**10**) (left) and **40** (right) to act as membrane disruptors in *P. aeruginosa* PAO1 as determined using a nitrocefin hydrolysis assay. Polymixin B (PMB) was the positive control (128 µg/mL) and the negative control was bacteria with nitrocefin.
